# The Role of Zn–Hf Site Proximity and Oxygen Vacancies for Methanol Formation Over ZnHfO*_x_* Catalysts Under CO_2_ Hydrogenation Conditions

**DOI:** 10.1002/anie.8850754

**Published:** 2026-06-30

**Authors:** Alexander Oing, Diana Piankova, Jean C. Villa‐Arpi, Muhammad Helmi Risansyauqi, Hector Prats, Felix Donat, Paula M. Abdala, Aleix Comas‐Vives, Christoph R. Müller

**Affiliations:** ^1^ Department of Mechanical and Process Engineering ETH Zürich Zürich Switzerland; ^2^ Institute of Materials Chemistry TU Wien Vienna Austria; ^3^ Departament De Ciència de Materials i Química Física & Institut De Química Teòrica i Computacional (IQTCUB) Universitat De Barcelona Barcelona Spain

**Keywords:** CO_2_ hydrogenation, hafnia, heterogeneous catalysis, operando spectroscopy, zinc oxide

## Abstract

Mixed metal oxides, such as ZnZrO*_x_*, have attracted considerable interest as CO_2_ hydrogenation to methanol catalysts due to their high methanol selectivity (> 70%) and catalytic stability at elevated reaction temperatures (> 300°C). In this work, we introduce a novel ZnHfO*_x_* catalyst that exceeds the intrinsic methanol formation rate of the reference ZnZrO*_x_* at a Zn content of 20 mol% (*r*_MeOH,20ZnZrOx_ = 0.59 mol_MeOH_(mol_cat_h)^−1^, *r*_MeOH,20ZnHfOx_ = 0.68 mol_MeOH_(mol_cat_h)^−1^). Remarkably and in contrast to ZnZrO*_x_*, the ZnHfO*_x_*‐based catalysts exhibit a high methanol selectivity (> 70%) up to Zn contents of 99.5 mol% despite the segregation of ZnO. Operando spectroscopy, in combination with computational analysis, identifies the Zn–V_O_–Hf motif as the active site for methanol formation that proceeds via the formate‐methoxy pathway. Such active sites are not only present in solid solution‐type ZnHfO*_x_* catalysts (≤ 35 mol% Zn), but also in the form of isolated HfO*_x_* clusters on segregated ZnO surfaces (for high Zn contents of > 35 mol%), explaining the high selectivity (and activity) over a wide range of Zn contents.

## Graphical abstract

ZnHfO*_x_* catalysts enable highly selective hydrogenation of CO_2_ to methanol over a wide range of Zn contents, as the Zn‐V_O_‐Hf active motif (V_O_ = oxygen vacancy) exists not only in solid solution‐type ZnHfO*_x_* for low Zn contents (≤ 35 mol%), but also as HfO*_x_* cluster/single atom decorated ZnO surfaces for Zn contents > 95 mol%. The formation of the active site over a wide range of Zn contents is facilitated through strong interactions between the oxides ZnO and HfO_2_.

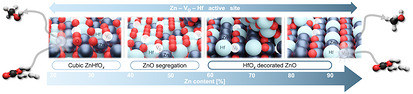

## 1 Introduction

The increasing anthropogenic CO_2_ emissions and the resulting consequences for the global climate require technological solutions to capture and store CO_2_ and/or close the carbon cycle. One key chemical in a sustainable carbon cycle is methanol [1, 2, 3], which can be produced sustainably by reacting captured CO_2_ with renewable H_2_ (produced, e.g., through water electrolysis). Methanol can directly serve as a fuel or be converted further into, for example, olefines via established processes [1, 4]. While methanol production from a synthesis gas (a mixture of CO and H_2_) is a routinely employed process, synthesis via the hydrogenation of CO_2_ remains a challenging catalytic reaction. The CO_2_ hydrogenation kinetics are sluggish for temperatures < 250°C, whereas increasing the reaction temperature favors thermodynamically the competing reverse water‐gas shift (RWGS) reaction, leading to a high selectivity towards CO instead of methanol [5, 6].

Further, the current benchmark catalyst for the hydrogenation of CO_2_ to methanol, namely, Cu/ZnO/Al_2_O_3_, deactivates under industrially relevant conditions (*p* = 30–60 bar, *T* = 200°C–250°C, and gas hourly space velocity (GHSV) = 900–1300 h^−1^), largely due to the water‐mediated sintering of ZnO and Cu oxidation [7, 8, 9]. Attempts to mitigate some of the shortcomings of Cu/ZnO/Al_2_O_3_ have focused on bimetallic catalysts [10, 11, 12, 13, 14, 15] and metal oxide‐based catalysts [16, 17, 18, 19]. Metal oxide‐based catalysts are a particularly promising solution to combat steam‐induced deactivation. In_2_O_3_ was the first reported purely metal oxide‐based catalyst for the hydrogenation of CO_2_ to methanol, yielding a high intrinsic activity and stable performance when supported on ZrO_2_ (CO_2_ conversion of 3%–5%, methanol selectivity of up to 85% for 1000 h at 50 bar, 280°C, and 24 L(g_cat_h)^−1^) [18, 20]. More recently, mixed metal oxides relying on more abundant metals than indium have become the focus of research [21, 22]. Within this family of materials, ZnZrO*_x_*‐based mixed oxides are very promising due to their high methanol selectivity (> 80%) at elevated temperatures (> 300°C) and high stability [23]. Indeed, ZnZrO*_x_* has been explored on a pilot plant‐scale, yielding an annual methanol production of 1200–1500 tons (CO_2_ conversion of 94.6% at 61 bar, operating temperature 300°C–315°C, and space velocity of 8000 h^−1^) [24].

Concerning the structure of ZnZrO*_x_* catalysts (typically prepared by co‐precipitation), initial reports pointed to a solid solution in which Zn is incorporated into the structure of tetragonal ZrO_2_ [23]. The substitution of Zr^4+^ with Zn^2+^ cations in the ZrO_2_ host lattice induces structural defects, that is, oxygen vacancies (V_O_), that have been identified to be a part of the Zn–V_O_–Zr active motif for CO_2_ hydrogenation to methanol, which proceeds through the formate‐methoxy reaction pathway [25, 26, 27]. Although it has been established in the meantime that co‐precipitated ZnZrO*_x_* does not constitute a perfect solid solution, but rather a heterogeneous mixture of Zn‐doped tetragonal ZrO_2_ and sub‐nanometer sized ZnO clusters [28, 29], it is generally accepted that the high dispersion of Zn within the catalyst is crucial to obtain a high methanol selectivity. Indeed, it was observed that when the Zn/(Zn+Zr) ratio exceeds approximately 30 mol%, bulk, hexagonal ZnO segregated, which was paralleled by an increased formation of CO through the competing RWGS reaction, thus lowering the methanol selectivity of the catalyst [23, 30, 31]. Hence, the optimum Zn content in ZnZrO*_x_*‐based catalysts with regard to maximizing methanol formation rate is commonly reported at ∼20 mol% [24, 32, 33, 34].

Several studies have attempted to further increase the methanol yield of ZnZrO*_x_*‐based catalysts, including the use of alternative synthesis methods such as flame spray pyrolysis [35] or the addition of metal promoters. The synthesis of ZnZrO*_x_* by flame spray pyrolysis yielded catalysts with a relatively high specific surface area (up to 90 m^2^ g^−1^) while maintaining well‐dispersed Zn species [35]. The addition of metal promoters such as Ga [36, 37], Cu [37, 38], or Pd [39, 40, 41] facilitates H_2_ activation, yielding an increase in methanol formation rates by up to 100% compared to unmodified ZnZrO*_x_*. Considering these advances in the ZnZrO*_x_* system, future material modifications might lead only to relatively small additional improvements.

Here, we introduce a novel catalyst formulation for the hydrogenation of CO_2_ to methanol that is based on ZnHfO*_x_*. Hf was selected due to its chemical and structural similarity to Zr [42, 43]. Both ZrO_2_ and HfO_2_ crystallize mainly in monoclinic, tetragonal, or cubic phases [44], although also orthorhombic crystal structures have been reported for both systems [45, 46, 47, 48]. In addition, Hf accounts for ∼1.5–14 wt.% of natural Zr ores and is very difficult to separate from Zr [49]. Consequently, commercial ZrO_2_ contains up to 2 wt.% Hf, and even when purchased as a highly purified chemical (e.g., reagent grade), Zr compounds contain trace amounts of Hf at ppm level [50]. Despite its chemical similarity to ZrO_2_, HfO_2_ remains largely underexplored in heterogeneous thermal catalysis [51].

The most active catalyst formulation identified in this work can be described as Zn‐doped HfO_2_ (ZnHfO*_x_*), which shows a high methanol selectivity (> 80%), surpassing the intrinsic methanol formation rate of the reference ZnZrO_x_ catalyst at 20 mol% Zn content (*r*_MeOH,20ZnZrOx_ = 0.59 mol_MeOH_(mol_cat_h)^−1^, *r*_MeOH,20ZnHfOx_ =  0.68 mol_MeOH_(mol_cat_h)^−1^ at 20 bar, 320°C, 24 L(g_cat_h)^−1^). Remarkably, unlike ZnZrO*_x_*, ZnHfO*_x_* catalysts maintain a high methanol selectivity (≥ 70%) at Zn contents of up to 99.5 mol%, at which most of the catalyst is in fact comprised of bulk ZnO. This is a very important finding, both from a fundamental point of view, but also economically, as the cost of Zn is 70% and > 99% lower than that of Zr and Hf, respectively [52]. Concerning the reaction pathway, operando diffuse reflectance infrared Fourier transform spectroscopy (DRIFTS) points to the formate‐methoxy reaction pathway, while operando UV‐Vis spectroscopy, high resolution electron microscopy, and further complementary surface chemistry analysis indicate Zn–V_O_–Hf to be the active motif. Considering the high selectivity of the catalysts at very high Zn contents (i.e., 99.5 mol% Zn), such Zn–V_O_–Hf‐type motifs also form on ZnO, as its surface is found to be decorated with HfO*_x_* clusters.

## 2 Results and Discussion

### 2.1 Structural Characterization

ZnHfO*_x_* catalysts of varying Zn:Hf ratios were synthesized by co‐precipitating ZnCl_2_ and HfCl_4_ at pH 7, followed by calcination in air at 580°C, if not indicated otherwise. Two distinctly different material systems formed after calcination: (i) catalysts without the formation of bulk, hexagonal ZnO for Zn contents < 40 mol% (described first), and (ii) catalysts that exhibited the presence of hexagonal bulk ZnO crystallites for Zn contents > 40 mol% (described second).

### 2.2 ZnHfO*_x_* Catalysts Containing 10–40 mol% Zn

**FIGURE 1:**
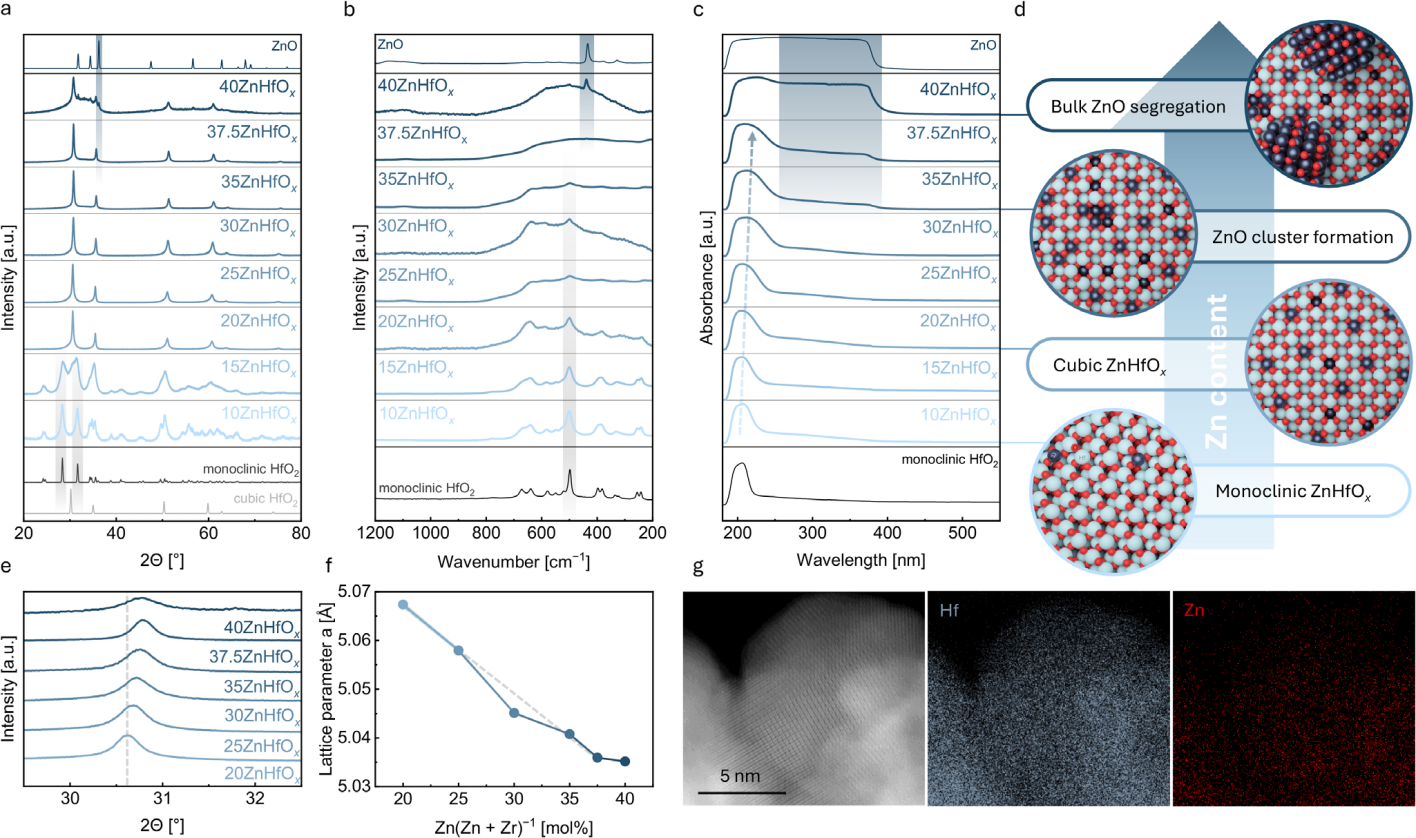
Structural characterization of as‐prepared ZnHfO*_x_* catalysts. (a) X‐ray diffraction patterns of ZnHfO*_x_* for Zn contents ranging from 10 to 40 mol%. The most prominent reflections due to monoclinic HfO_2_ are highlighted in grey, while the most prominent reflection due to ZnO is highlighted in blue. (b) Raman spectra of ZnHfO*_x_* for Zn contents ranging from 10 to 40 mol%. The most prominent Raman band due to monoclinic HfO_2_ is highlighted in grey, while the band due to ZnO is highlighted in blue. (c) UV‐Vis spectra of ZnHfO*_x_* with Zn contents ranging from 10 to 40 mol%. Dashed arrow indicates the bathochromic shift of the absorption due to Zn doping of HfO_2_. The region shaded in blue is due to absorption by (bulk) ZnO. (d) Illustration of the structural change of ZnHfO*_x_* with increasing Zn content. Dark blue spheres represent Zn, light blue spheres Hf, and red spheres O atoms. (e) Peak shift of the (111) reflection of cubic HfO_2_ with increasing Zn contents (due to Zn doping of HfO_2_). Dashed, grey line indicates the peak position of the (111) reflection of 20ZnHfO*_x_*. (f) Lattice parameter of cubic ZnHfO*_x_* as a function of the fraction of Zn contents. (g) High‐angle annular dark‐field (HAADF)—Scanning transmission electron microscopy (STEM) image, and STEM—Energy dispersive X‐ray (EDX) spectroscopy maps of 30ZnHfO*_x_*_._

XRD analysis revealed that undoped HfO_2_ and 10ZnHfO*_x_* crystallize in a monoclinic phase. Increasing the Zn content (15ZnHfO*_x_* to 40ZnHfO*_x_*) induces a phase transition to cubic ZnHfO*_x_* solid solutions (Figure 1a). The materials containing 20–40 mol% Zn (20ZnHfO*_x_* to 40ZnHfO*_x_*) exhibit predominantly a cubic phase with only very weak reflections due to small amounts of the monoclinic phase (Figure S1). Rietveld refinement showed a gradual decrease in the unit cell size of the cubic phase with increasing Zn content (for Zn fractions in the range of 20–40 mol%, Figure 1f) consistent with the substitution of larger Hf^4+^ cations with smaller Zn^2+^ cations into the structure (Figure S2). The lattice parameter did not decrease appreciably when increasing the Zn content from 37.5 to 40 mol%, which coincided with the appearance of reflections due to hexagonal ZnO (Figure S1). Thus, the apparent limit for the incorporation of Zn into cubic HfO_2_ was ∼ 37.5 mol%, a value that is higher compared to ZnZrO*_x_* (∼ 30 mol% Zn) (Figure S3) [28]. The stabilization of the cubic phase through Zn doping is in line with previous DFT calculations [53]. 40ZnHfO*_x_* exhibits also broad features across the XRD pattern, indicating the presence of an amorphous phase. These features are also present in 35ZnHfO*_x_* and 37.5ZnHfO*_x_*, yet less pronounced (Figure S1).

The local structure of ZnHfO*_x_* was probed further by Raman spectroscopy (Figure 1b) that shows the presence of bands due to monoclinic HfO_2_ for 10ZnHfO*_x_* and 15ZnHfO*_x_* in line with XRD observations. The most pronounced Raman band due to monoclinic HfO_2_, that is, the symmetric Hf‐O vibration [54] at 500 cm^−1^, was observed also for materials with Zn contents of up to 30 mol%, indicating the presence of small monoclinic, Zn‐doped HfO_2_ domains, likely at the surface of ZnHfO_x_. Moreover, the Raman bands due to Hf‐O vibrations (Figure 1b) continuously broadened and became less pronounced with increasing Zn contents, due to a distortion of the crystal lattice arising from the incorporation of Zn. For Zn contents > 35 mol%, Raman bands due to Hf‐O vibrations of the monoclinic HfO_2_ phase were not observed. Previous studies have reported a broad Raman vibration at ∼ 620 cm^−1^ for cubic HfO_2_ [55], yet, this Raman band was not observed here; however, ZnHfO*_x_* with Zn contents in the range 20–30 mol% showed a broad feature in the range 250–800 cm^−1^, which might be indicative of the presence of cubic HfO_2_ at the surface (Figure S4). The fact that the individual bands due to Hf‐O vibrations merge into one, broad feature for Zn contents > 30 mol% is indicative of a highly defective and distorted surface, which is also in line with the increasing content of an amorphous phase for Zn contents > 30 mol% as observed by XRD (Figure S1). For a Zn content of 37.5 mol%, a Raman band characteristic for hexagonal ZnO appeared at 438 cm^−1^, albeit at very low intensity, and it was present only in some of the recorded spectra, confirming the segregation of hexagonal ZnO for Zn contents ≥ 37.5 mol%, as also observed by XRD in Figure 1a.

To probe ZnO segregation in detail, UV‐Vis spectroscopy, owing to its high sensitivity towards crystalline ZnO, was employed [29]. Monoclinic HfO_2_ has a characteristic light absorption of wavelengths < 250 nm (electronic transition from the O *2p* valence band to the Hf *5d* conduction band) [56]. Hexagonal ZnO absorbs light at wavelengths < 400 nm through the excitation of electrons from the upper O 2*p* valence band and hybridized Zn 3*d* states to the conduction band of Zn 4*s* states [57]. For Zn contents ≤ 30 mol%, UV‐Vis spectroscopy showed no characteristic ZnO absorption bands (Figure 1c). However, starting at Zn contents of 35 mol%, a small absorption band due to ZnO appeared, indicating the segregation and formation of nanocrystalline ZnO domains that were neither detected by XRD nor by Raman spectroscopy (both XRD and Raman spectroscopy show signatures of ZnO for Zn contents ≥ 37.5 mol%). Furthermore, a bathochromic shift of the absorbance band of HfO_2_ for increasing Zn contents was observed, indicating that the electronic structure of HfO_2_ was altered by Zn doping. The UV‐Vis spectra of ZnHfO*_x_* were analyzed in a Tauc plot to determine the bandgap energy *E*_g_ of the materials and to provide evidence for the effect of Zn doping on the electronic structure of HfO_2_ (Figure S5). The bandgap of undoped, monoclinic HfO_2_ was determined as 5.64 eV, which agrees well with theoretical and experimental values reported in the literature [58, 59]. With Zn doping, the energy of the bandgap decreased, reaching ∼ 5.08 eV for Zn contents in the range 15–20 mol% and 4.65 eV for 37.5ZnHfO*_x_*. This change in bandgap energy is further evidence for the substitutional doping of HfO_2_ by Zn [60, 61]. A reduction in the bandgap energy in metal oxides has been linked to the formation of oxygen vacancies upon the substitutional doping of metal oxides [62, 63, 64].

**FIGURE 2:**
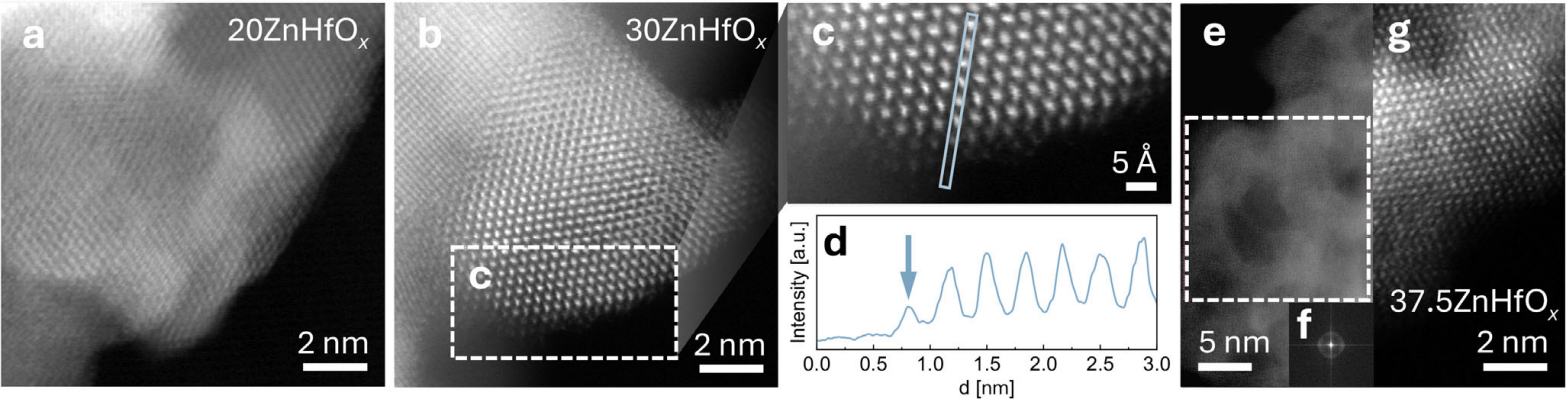
High resolution STEM analysis of ZnHfO_x_ catalysts. HAADF‐STEM images of (a) 20ZnHfO*_x_*, (b) 30ZnHfO*_x_*, and (e) 37.5ZnHfO*_x_*. (c) Enlarged view of the region marked in (b). (d) Line profiles of the HAADF signal intensity across the surface region in (c) (from bottom to top). (e) and (g) HAADF‐STEM images of 37.5ZnHfO*_x_* and (f) fast Fourier transform (FFT) pattern derived from the area marked by a white rectangle.

To obtain insight into the spatially resolved atomic‐scale structure of the series of ZnHfO*_x_* catalysts, scanning transmission electron microscopy (STEM) was employed (Figure 2). While energy‐dispersive x‐ray spectroscopy (EDX) STEM mapping showed an overall homogeneous spatial distribution of Zn and Hf in 20ZnHfO*_x_* and 30ZnHfO*_x_* (Figures S6 and S7), HAADF intensity profiles revealed a decrease in signal intensity toward the surface on 30ZnHfO*_x_* (Figure 2c,d). Given that HAADF‐STEM intensity scales with the atomic number Z as ∝ Z^1.6–1.9^ and considering the difference between the atomic numbers of Hf (Z = 72) and Zn (Z = 30) [65], this decrease in signal intensity apart from abrupt mass‐thickness effect can also originate from a Zn enrichment at the surface (disentangling these effects can be done only via image simulations). However, XRD indicated the formation of a single solid solution phase for 30ZnHfO*_x_*, and such surface enrichment of Zn was not seen in 20ZnHfO*_x_* (Figure S8). The possible surface enrichment of Zn could be a state that precedes the formation and segregation of ZnO cluster/particles for higher Zn contents (a similar surface enrichment of Zn has been reported for ZnZrO*_x_* [23, 35]). For higher Zn contents, that is ≥ 37.5 mol%, STEM analysis revealed the presence of both crystalline (Figure 2g) and amorphous particles (Figure 2e,f). The presence of an amorphous ZnHfO*_x_* phase in 37.5ZnHfO*_x_* and in 40ZnHfO*_x_* is consistent with XRD analysis (Figure S1). In contrast to 20ZnHfO*_x_* and 30ZnHfO*_x_*, STEM‐EDX mapping of 37.5ZnHfO*_x_* allows to identify regions that correspond to a ZnO phase in addition to regions of homogeneously distributed Zn and Hf (Figure S9), which confirms the segregation of ZnO in 37.5ZnHfO*_x_*, supporting XRD, Raman, and UV‐Vis spectroscopy analyses.

### 2.3 ZnHfO*_x_* Catalysts Containing > 40 mol% Zn

**FIGURE 3:**
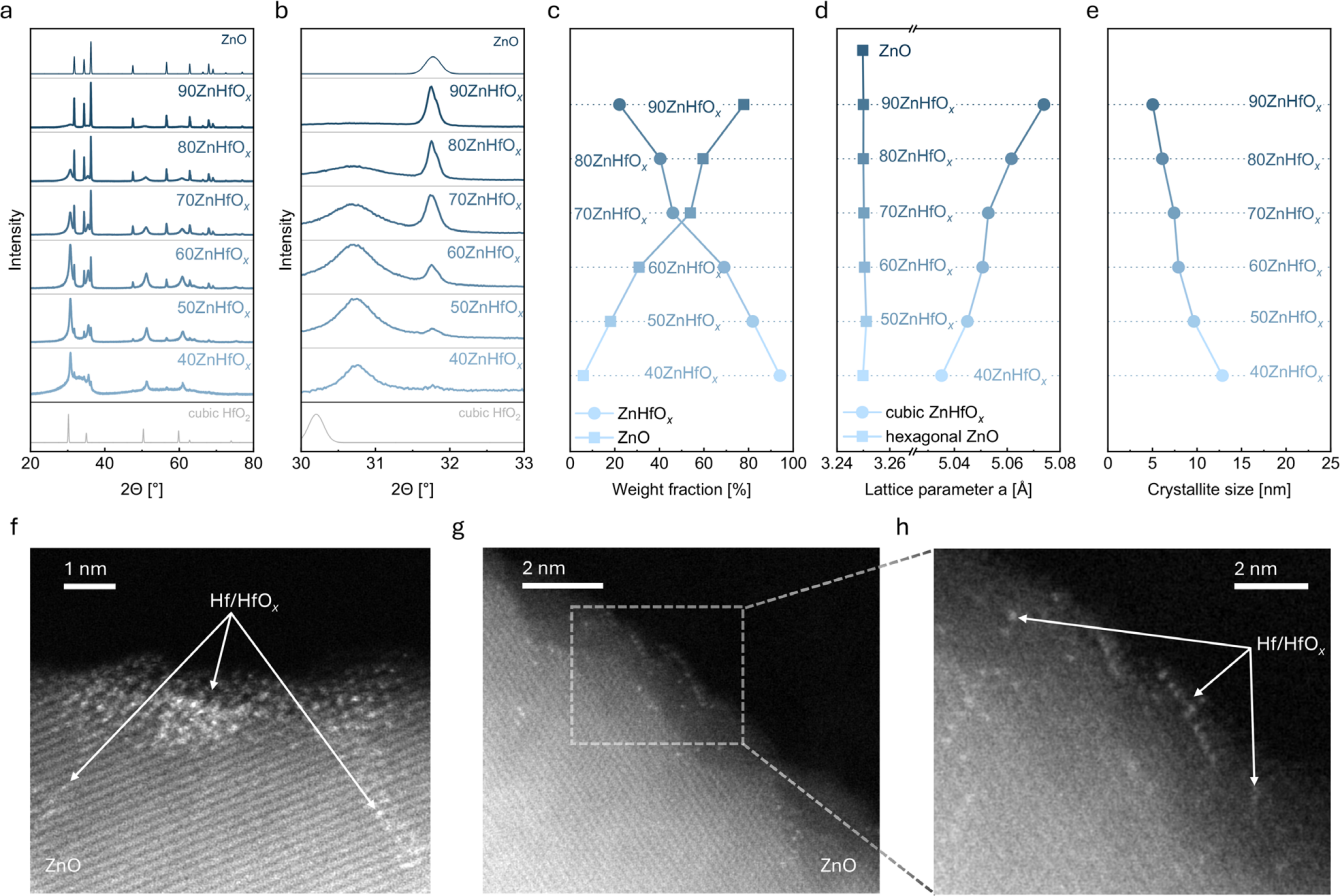
XRD analysis of as‐prepared ZnHfO*_x_* catalysts with Zn contents ranging from 40 to 90 mol%. (a) XRD patterns of ZnHfO*_x_* catalysts with Zn contents ranging from 40 to 90 mol%. (b) Enlargement of the XRD region 2*θ* = 30°–33° of the XRD patterns shown in (a). (c) Weight fractions of cubic ZnHfO*_x_* and segregated, hexagonal ZnO determined by Rietveld refinement. (d) Lattice parameter a of cubic ZnHfO*_x_* and segregated, hexagonal ZnO determined by Rietveld refinement. (e) Average crystallite size of cubic ZnHfO*_x_* determined using the Scherrer equation. (e–f) High resolution HAADF‐STEM images of a 98ZnHfO*_x_* surface evidencing the presence of HfO*_x_* clusters and single Hf atoms on the ZnO surface.

For Zn contents > 40 mol%, reflections in the XRD patterns due to cubic ZnHfO*_x_* gradually decreased in intensity with increasing Zn contents, while the intensity of the reflections due to hexagonal ZnO increased (Figures 3a,b, and S10). Using Rietveld refinement (Figure S11), we observed that the weight fraction of hexagonal ZnO increased from ∼ 6 wt.% in 40ZnHfO*_x_* to ∼ 78 wt.% in 90ZnHfO*_x_* (Figure 3c). Independent of the Zn content, the lattice parameter a of the (segregated) hexagonal ZnO phase did not change noticeably with Zn content, suggesting that Hf was not incorporated in significant amounts into the bulk ZnO structure (Figure 3d). However, the lattice parameter of cubic ZnHfO*_x_* increased monotonically for Zn contents > 40 mol%, indicating that there was less Zn incorporated into the ZnHfO*_x_* structure compared to catalysts without ZnO segregation (Zn contents < 37.5 mol%). In addition, the reflections due to cubic ZnHfO*_x_* broadened continuously with increasing Zn content, indicative of the formation of increasingly smaller crystallites. Using the Scherrer equation, the average size of the doped ZnHfO*_x_* crystallites decreased from ∼ 14 nm in 40ZnHfO*_x_* to ∼ 5 nm in 90ZnHfO*_x_* (Figure 3e).

The segregation of hexagonal ZnO for Zn contents > 40 mol% was also supported by Raman and UV‐Vis spectroscopy, showing characteristic ZnO bands (Figures S10 and S12). Importantly, in all ZnHfO*_x_* catalysts that contained segregated ZnO, the characteristic ZnO vibration in the Raman spectra was, on average, shifted to higher wavenumbers compared to pure ZnO (Figure S13). In the most pronounced case (50ZnHfO*_x_*), the ZnO vibration shifted on average to ∼ 438 cm^−1^ compared to 343 cm^−1^ for pure ZnO. A Raman shift to higher wavenumbers has been attributed to lattice strain induced by doping of ZnO [66], hence, hinting at some incorporation of Hf into ZnO. Analogously, the ZnO bandgap increased slightly from 2.5 eV for pure ZnO to 2.7–2.9 eV for segregated ZnO in the catalysts with Zn contents 50–90 mol% (Figure S14), providing another indication for Hf doping of ZnO. An increase in the bandgap of Hf‐doped ZnO when compared to ZnO has been reported before [67]. However, the absence of a distinguishable change in the lattice parameter of ZnO in the ZnHfO*_x_* catalyst series (as determined by Rietveld analysis) and the rather small change in bandgap energy suggest that the doping of ZnO with Hf only occurs in the surface region of the ZnO domains.

STEM imaging combined with EDX‐STEM mapping of Zn‐rich materials (90ZnHfO*_x_* and 98ZnHfO*_x_*) revealed the presence of two distinct phases, that is, large ZnO crystals and smaller, nano‐sized HfO_2_‐based crystallites (Figures S15 and S16). Resolving further the surface of the ZnO crystallites of 98ZnHfO*_x_* by HAADF‐STEM showed small HfO*_x_* clusters (and/or single Hf atoms) on the surface of ZnO (due to the high atomic number of Hf compared to Zn, Hf is clearly distinguishable) (Figure 3e,f). HfO*_x_* clusters could also be resolved on the ZnO surfaces of 90ZnHfO*_x_* (Figure S18).

Summarizing, the co‐precipitated ZnHfO*_x_* catalyst series crystallized as Zn‐doped, cubic HfO_2_ (yet containing also monoclinic HfO_2_ in small amounts) for Zn contents ranging from 20 to 35 mol%. For Zn contents > 35 mol%, a ZnHfO*_x_* solid solution and segregated hexagonal ZnO were present. While Hf was not incorporated in significant amounts into the bulk structure of segregated ZnO, small HfO*_x_* clusters were present on the surface of ZnO, and some Hf was likely incorporated into the surface layers of ZnO.

### 2.4 Catalytic Performance of ZnHfO*_x_* Catalysts

**FIGURE 4:**
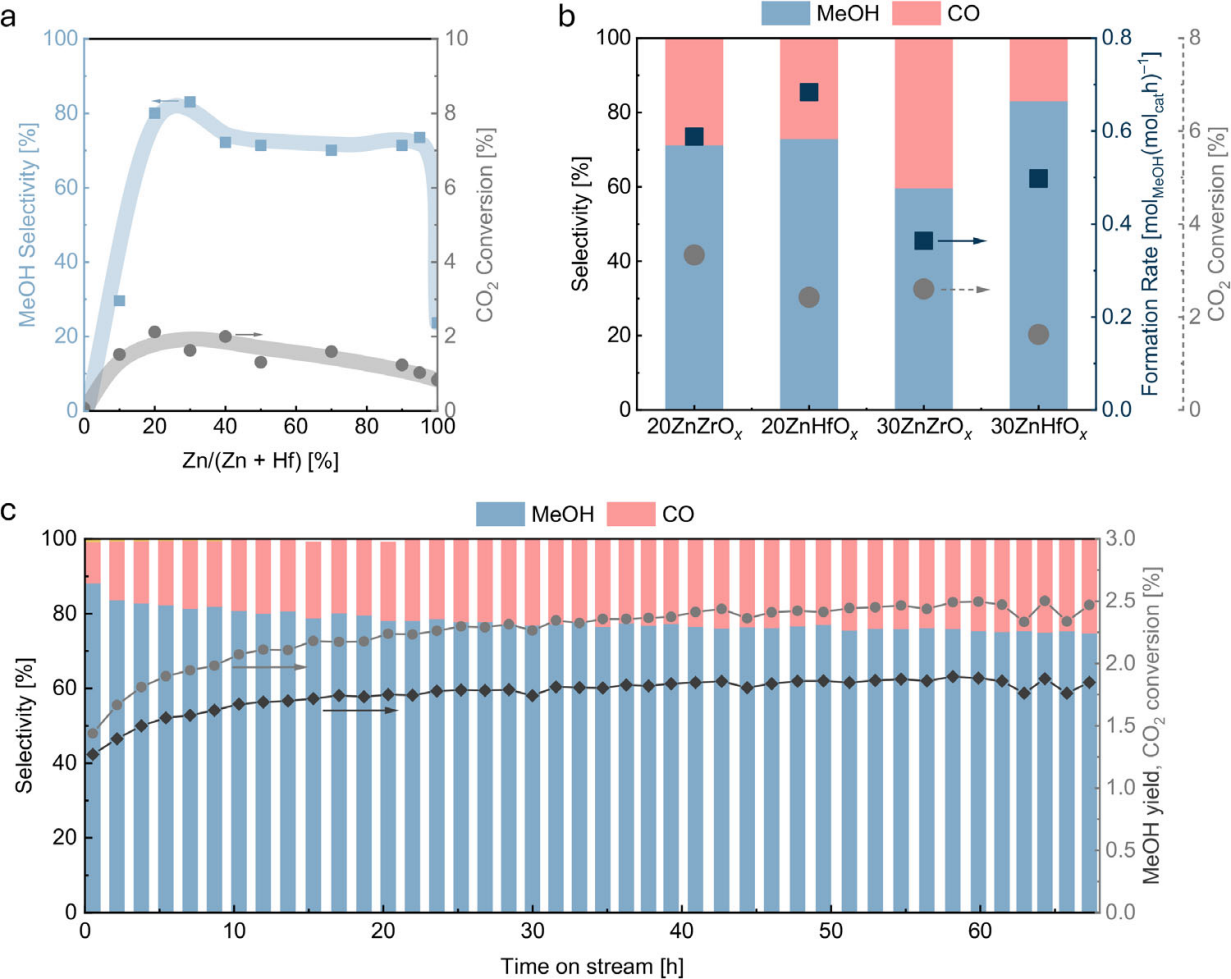
Catalytic performance of ZnHfO*_x_* catalysts. (a) Methanol selectivity and CO_2_ conversion of ZnHfO*_x_* for Zn contents ranging from 0 to 100 mol%. The performance of each catalyst is compared after approximately 9 h of time on stream at 20 bar, 320°C, H_2_:CO_2_:N_2_ = 3:1:1, and 24 L(g_cat_h)^−1^. The colored bands guide the eye for the trends of methanol selectivity (light blue) and CO_2_ conversion (light grey). (b) Comparison of the catalytic performance of selected ZnZrO*_x_* and ZnHfO*_x_* catalysts at 20 bar, 320°C, H_2_:CO_2_:N_2_ = 3:1:1, and 24 L(g_cat_h)^−1^. (c) Evolution of product selectivities and CO_2_ conversion of 30ZnHfO*_x_* over 70 h of time on stream at 20 bar, 320°C, H_2_:CO_2_:N_2_ = 3:1:1, and 24 L(g_cat_h)^−1^.

The catalytic performance of the series of ZnHfO_x_‐based catalysts was evaluated in a fixed‐bed reactor at 20 bar, 320°C, H_2_:CO_2_:N_2_ = 3:1:1, and a GHSV of 24 L(g_cat_h)^−1^ for Zn contents ranging from 0 to 100 mol% (Figure 4a). Pure HfO_2_ showed no activity for the hydrogenation of CO_2_ to methanol, whereas pure ZnO formed methanol with a selectivity of 23% at a CO_2_ conversion of 0.83%. A significantly higher methanol selectivity and CO_2_ conversion were observed for the ZnHfO*_x_* catalysts, with CO always being the main side‐product of the reaction. The formation of other side‐products was negligible, with the exception of 40ZnHfO*_x_*, which produced small amounts of dimethyl ether (DME) with a selectivity of 2 %, and 70ZnHfO*_x_* that produced methane with a selectivity of 1% (Figure S19). The methanol selectivity reached a maximum at 83 % for 30ZnHfO_x_, and, remarkably, consistently exceeded > 70 % for Zn contents of up to 99.5 mol% (Figure S20) despite the segregation of hexagonal ZnO in such materials, which in its pure form produces largely CO (also ZnZrO_x_‐based catalysts produce predominantly CO for high Zn contents) (Figure 4a) [23, 30, 31]. Decreasing the temperature to 280°C increased the methanol selectivity to > 80 % for the catalysts with a Zn content in the range 20–95 mol% (Figure S21). At 20 bar and 320°C, the maximum CO_2_ conversion was 2.2 % for 20ZnHfO*_x_*, decreased slightly to 1.6% for 70ZnHfO*_x_*, and still reaching 1.2% for 95ZnHfO*_x_* (the thermodynamic equilibrium conversion at the given conditions is ∼ 26%, Figure S22). 30ZnHfO*_x_* and 50ZnHfO*_x_* exhibited slightly lower CO_2_ conversions compared to the overall trend of the ZnHfO*_x_* series, which may be attributed to slightly lower specific surface areas for these two catalysts (Table S1). The reproducibility of the performance results for selected catalysts (20ZnHfO*_x_* and 90ZnHfO*_x_*) is presented in the Supporting Information (Figure S23).

When tested under CO_2_ hydrogenation conditions at 20 bar and 320°C, all catalysts activated with time on stream, that is, the CO_2_ conversion increased at slightly decreasing methanol selectivity (Figure S19). For example, the overall methanol yield of 30ZnHfO*_x_* increased by more than 370 % over the first 9 h of reaction and increased by only an additional 3 % over the following 3 h. When comparing the catalytic performance of 20ZnHfO*_x_* to the reference 20ZnZrO*_x_* at identical reaction conditions, both catalysts exhibited a similar methanol selectivity, whereas the CO_2_ conversion of 20ZnHfO*_x_* was 28 % lower (Figure 4b). However, Hf has approximately twice the molar mass of Zr; hence, when normalizing methanol yield by mole of catalyst instead of gram catalyst, 20ZnHfO*_x_* possessed a 15% higher intrinsic methanol formation rate of 0.68 mol_MeOH_(mol_cat_h)^−1^ compared to 20ZnZrO*_x_* at 0.59 mol_MeOH_(mol_cat_h)^−1^. At higher Zn contents, 30ZnHfO*_x_* (0.5 mol_MeOH_(mol_cat_h)^−1^) outperformed 30ZnZrO*_x_* (0.36 mol_MeOH_(mol_cat_h)^−1^) by an even larger margin (36%) since the ZnHfO_x_ series (unlike the ZnZrO*_x_* series [23]) shows a consistently high methanol selectivity for a broad range of Zn contents (20–99.5 mol%) (Figures 4a and S20). The same trends were observed when normalizing the methanol formation rates to the surface area of the catalysts (Figure S24). Next, 20ZnHfO*_x_*, that is, the best performing catalyst based on methanol formation rate, was exposed to 80 h of time on stream to probe both its activation dynamics and long‐term stability (Figure 4c). The catalyst was fully activated after approximately 50 h on stream, reached a CO_2_ conversion of 2.5%, and showed an excellent stability by maintaining its methanol formation during the subsequent 30 h on stream. Similarly, 90ZnHfO*_x_* also activated during the first 25 h of time on stream, followed by a stable methanol production for the remaining 20 h of the experiment, demonstrating a high stability (Figure S25). 20ZnHfO*_x_* was also evaluated at 50 bar and 320°C, which doubled the CO_2_ conversion (4.3%) compared to 20 bar, while maintaining a methanol selectivity of approximately 80% (Figure S26).

To rationalize the high methanol selectivity of ZnHfO*_x_* for Zn contents > 40% that contain a considerable amount of ZnO crystallites, a physical mixture of ZnO and (monoclinic) HfO_2_ (ZnHfO*_x_*_‐PM_, calcined at 580°C) with Zn contents of 20, 30, and 90 mol% was compared to the respective ZnHfO_x_ catalyst prepared by co‐precipitation. As expected, the XRD patterns of all ZnHfO*_x_*_‐PM_ catalysts (Figure S27) show pronounced reflections due to hexagonal ZnO, confirming the presence of bulk ZnO in all physically mixed catalysts. The co‐precipitated ZnHfO*_x_* catalyst outperformed the physically mixed counterparts in terms of CO_2_ conversion and methanol selectivity for all investigated Zn contents, yielding methanol formation rates that were 46%–60% higher (Figure S28). Further, the ZnHfO*_x_* catalysts were also compared to ZnZrO*_x_*_‐PM_ catalysts prepared by physically mixing ZnO with ZrO_2_, followed by calcination at 580°C (Figure S30). At a given Zn content, all ZnHfO*_x_* and ZnHfO*_x_*_‐PM_ catalysts exhibited higher methanol selectivities than ZnZrO*_x_*_‐PM_ catalysts, resulting in up to 300% higher intrinsic methanol formation rates (Figure S28). It is worth noting that even at a Zn content of 90 mol%, 90ZnHfO*_x_*_‐PM_ reached a methanol selectivity of almost 60%, while the methanol selectivity of 90ZnZrO*_x_*_‐PM_ was 33%, which was close to that of pure ZnO (23%).

In an additional control experiment, a layered bed of ZnO and HfO_2_ with a molar ratio of ZnO:HfO_2_ = 90:10 was used to assess whether a physical contact between ZnO and HfO_2_ was critical to achieve high methanol selectivity; HfO_2_ was placed downstream of ZnO, and the pure oxides were physically separated by a plug of quartz wool to eliminate any interface/direct contact between them. The CO_2_ conversion and methanol selectivity recorded in the layered bed experiment were identical to the values obtained for pure ZnO, highlighting the importance of an interface between ZnO and HfO_2_ to attain a high methanol selectivity (Figure S31). In a last control experiment, a physical mixture of ZnO:HfO_2_ = 90:10, yet uncalcined prior to the catalytic test, exhibited a continuous increase in its methanol selectivity with time on stream, however, reaching only ∼ 40% methanol selectivity after 6 h of time on stream, compared to a stable methanol selectivity of almost 60% for the pre‐calcined (580°C) 90ZnHfO*_x_*_‐PM_ (Figure S32). When comparing the UV‐Vis spectra of the pre‐calcined and uncalcined ZnHfO*_x_*_‐PM_ catalysts, it is evident that the intensity of the absorbance band due to ZnO (wavelength < 400 nm) decreased after calcination (Figure S33), indicating a decrease in bulk ZnO absorbance through, for example, a surface dispersion of HfO*_x_* clusters onto the ZnO surface due to oxide‐oxide interactions. Moreover, Tauc plots revealed a small increase (0.1–0.2 eV) in the ZnO bandgap for 20ZnHfO*_x_*_‐PM_ and 30ZnHfO*_x_*_‐PM_ when compared to the uncalcined catalysts, pointing also to a migration and partial incorporation of HfO*_x_* clusters at the surface of ZnO (Figure S34). Indeed, high‐resolution HAADF‐STEM images of 90ZnHfO*_x_*_‐PM_ revealed a strong interaction between the individual ZnO and HfO_2_ particles and confirmed the migration of HfO*_x_* clusters onto the surface of ZnO crystals (Figure S35). However, for 90ZnZrO*_x_*_‐PM_, ADF‐STEM analysis did not indicate any migration of Zr/ZrO*_x_* onto ZnO (Figure S36), while exhibiting also weaker interaction between the ZnO and ZrO_2_ particles (Figure S37), explaining in turn the lower methanol selectivity of physically mixed ZnO and ZrO_2_.

### 2.5 Activation of the ZnHfO*_x_* Catalysts

Turning to the activation behavior with time on stream of the ZnHfO*_x_*‐based catalysts, catalysts with Zn contents < 40 mol% were found to activate to a larger degree than catalysts that contained a very high Zn content, for example, 90ZnHfO*_x_* or 95ZnHfO*_x_* (Figure S19). It is also noted that pure ZnO (Figure S38) and ZnHfO*_x_*_‐PM_ (calcined) exhibited no or only very little activation, even for low Zn contents (Figure S39). These observations indicate that activation was due to structural changes in the ZnHfO*_x_* solid solution‐type phase or changes at the interface of ZnO and ZnHfO*_x_*.

A control experiment established that the activated state of ZnHfO*_x_* was preserved when cooling down to room temperature and exposure to air (Figure S40). This observation allowed us to probe changes in the catalyst structure during activation by performing ex situ measurements (Figures S41–S43). XRD of the activated and as‐prepared (i.e., calcined) catalysts showed no distinct changes in the diffraction patterns; also, Rietveld refinement of the diffractogram of the activated catalysts yielded lattice parameters that were identical to the as‐prepared materials, underlining that the bulk structure of the catalysts did not change during activation. In addition, N_2_ physisorption analysis of 20ZnHfO*_x_* revealed no changes in the catalyst's surface area when comparing its as‐prepared and activated states (Figure S44). Consequently, activation must be due to changes at the surface of the catalysts.

Raman spectroscopy analysis revealed strong fluorescence of activated 20ZnHfO*_x_* and 30ZnHfO*_x_*, while fluorescence was largely absent in the respective as‐prepared catalysts (Figure S45). One potential origin for fluorescence in Raman spectroscopy of metal oxides is the presence of structural surface defects such as oxygen vacancies [68], which have been put forward as the active sites for CO_2_ activation in metal oxide‐based CO_2_ hydrogenation catalysts [17, 25, 27]. An increase in the surface density of oxygen vacancies would hence explain the increase in catalyst activity (while maintaining its selectivity) under reaction conditions for catalyst compositions that are rich in the ZnHfO*_x_* solid solution phase.

To provide evidence for oxygen vacancy formation‐triggered activation of ZnHfO*_x_*‐based catalysts under reaction conditions, operando UV‐Vis spectroscopy experiments were conducted (Figure S46). Here, as‐prepared 20ZnHfO*_x_* was exposed to a gas mixture of H_2_:CO_2_:N_2_ = 3:1:1 (24 L(g_cat_h)^−1^) at 20 bar and 320°C for ∼ 24 h. While the main wavelength regions of HfO_2_ (180–250 nm) and ZnO (200–400 nm) did not exhibit any significant changes over 24 h of time on stream, 20ZnHfO*_x_* revealed an increasing absorbance of wavelengths > 400 nm; this observation was paralleled by increasing methanol concentrations in the off‐gas as detected by mass spectrometry (MS). After 22 h on stream, the concentration of methanol in the off‐gas stabilized and concomitantly, the absorbance at wavelengths > 400 nm. A very similar trend was also observed for 30ZnHfO*_x_* (Figure S47). The increase in absorbance in the region > 400 nm has been attributed to the formation of oxygen vacancies (e.g., in In_2_O_3_‐based CO_2_ hydrogenation catalysts [69]) since oxygen vacancies can introduce new energy states (trap states) in the bandgap, allowing for the absorption of photons with an energy below the bandgap energy of the defect‐less state (*E*_g_ = 5.64 eV) [70, 71]. These results align with the observations made by Raman spectroscopy (Figure S45), correlating the activation of ZnHfO*_x_* with the generation of oxygen vacancies in the ZnHfO*_x_* solid solution phase under CO_2_ hydrogenation conditions.

**FIGURE 5:**
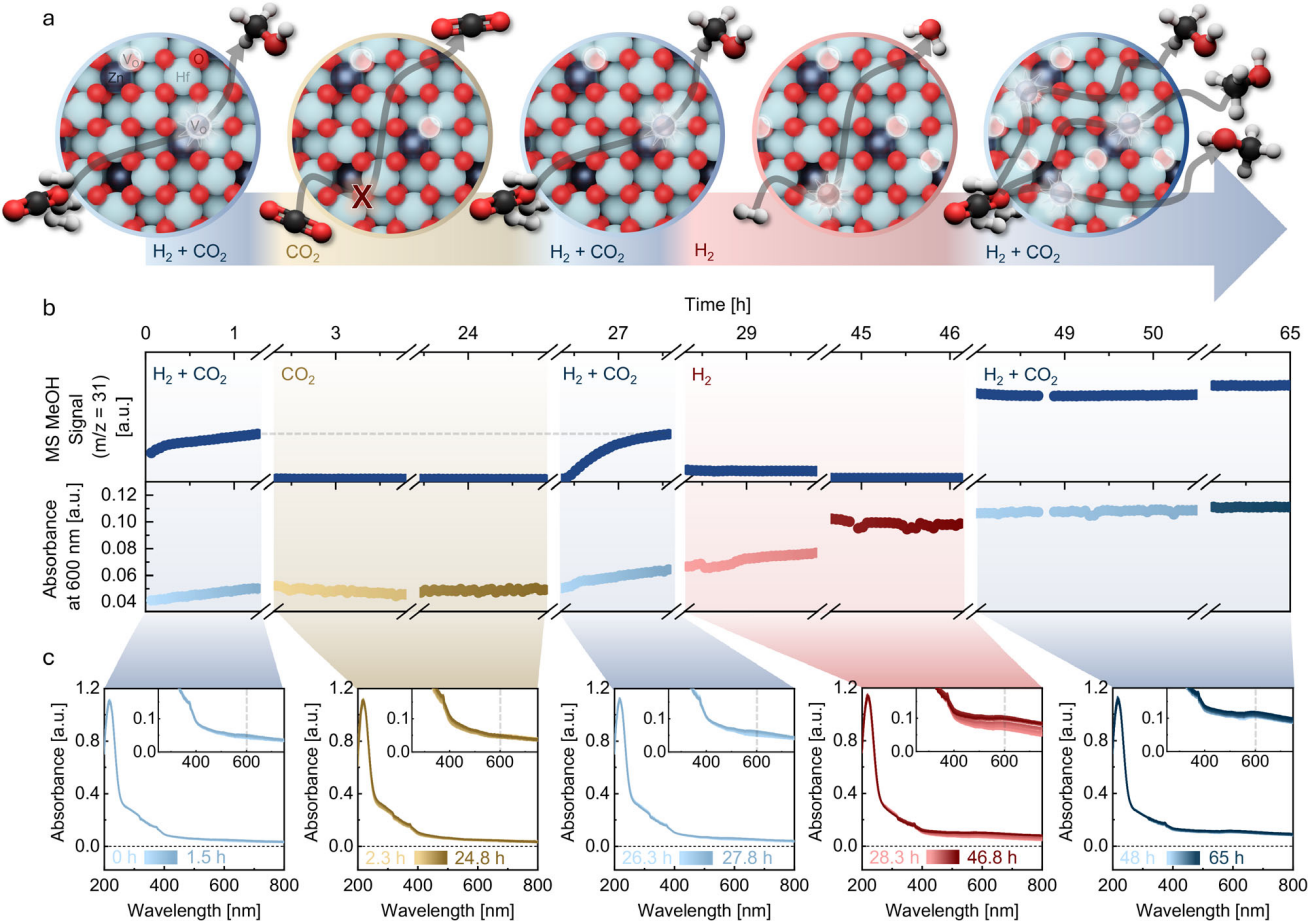
Operando UV‐Vis spectroscopy, gas‐switching experiment on 20ZnHfO*_x_*. (a) An illustration of the changes on the catalyst's surface under different gas atmospheres during an operando UV‐Vis spectroscopy, gas switching experiment. Dark blue spheres represent Zn, light blue spheres Hf, red spheres O atoms, and translucent white spheres represent oxygen vacancies. (b) Methanol formation tracked by MS (m/z = 31) and light absorbance of 20ZnHfO*_x_* at a wavelength of 600 nm during an operando UV‐vis spectroscopy, gas‐switching experiment at 20 bar, 320°C, and 24 L(g_cat_h) ^−1^. Gas flows were set as follows: H_2_:CO_2_:N_2_ = 3:1:1 for “H_2_ + CO_2_,” H_2_:CO_2_:N_2_ = 0:1:4 for “CO_2_,” and H_2_:CO_2_:N_2_ =  3:0:2 for “H_2_.” (c) Evolution of the full UV‐Vis spectra in the respective gas flows.

To verify the formation of (additional) oxygen vacancies during catalyst activation under CO_2_ hydrogenation conditions, an operando UV‐Vis spectroscopy experiment using 20ZnHfO*_x_* was conducted in which the gas flow was switched between (i) a CO_2_ hydrogenation atmosphere (H_2_:CO_2_:N_2_ = 3:1:1), (ii) CO_2_, and (iii) H_2_ (Figure 5). When 20ZnHfO*_x_* was kept under a CO_2_ hydrogenation atmosphere, the absorbance for wavelengths > 400 nm increased continuously, while the concentration of methanol in the off‐gas also increased. Subsequently, the gas flow was switched to CO_2_ (CO_2_:N_2_ = 1:4) for 22 h, during which the absorbance for wavelengths > 400 nm stayed constant. When switching the reaction atmosphere back to a CO_2_ hydrogenation atmosphere, a further growth in absorbance for wavelength > 400 nm was observed, accompanied by an increase in the methanol concentration in the off‐gas. When switching to H_2_ (H_2_:N_2_ = 3:2), the absorbance for wavelengths > 400 nm increased further, until its value stabilized after approximately 18 h. After switching the gas flow back to CO_2_ hydrogenation conditions, a significantly higher methanol formation rate compared to the previous two reaction steps was observed. In the following 16 h of time on stream, neither the methanol formation rate nor the absorbance for wavelength > 400 nm increased appreciably, demonstrating that the sample had been activated almost fully in 60 vol% H_2_ at 20 bar and 320°C for 18 h. The correlation between H_2_ treatment and the increase in absorbance for wavelengths > 400 nm provides strong evidence that the activation of ZnHfO_x_ originates from the (additional) formation of oxygen vacancies in ZnHfO_x_ under reducing conditions, with CO_2_ being unable to refill the oxygen vacancies in ZnHfO_x_ under the given reaction conditions. In this context, DFT calculations were performed to evaluate the energetics of oxygen vacancy formation in Zn‐doped HfO_2_ surfaces. As shown in Figure S48, Zn‐doped HfO_2_ containing an oxygen vacancy (ZnHfO*_x_*) is energetically favorable compared to Zn‐doped HfO_2_ without an oxygen vacancy (ZnHfO_2_), which features a negative oxygen vacancy formation energy of −3.98 eV. These results rationalize the experimentally observed presence of oxygen vacancies and their potential catalytic role in methanol synthesis.

### 2.6 Reaction Pathway of Methanol Formation Over ZnHfO*_x_*

**FIGURE 6:**
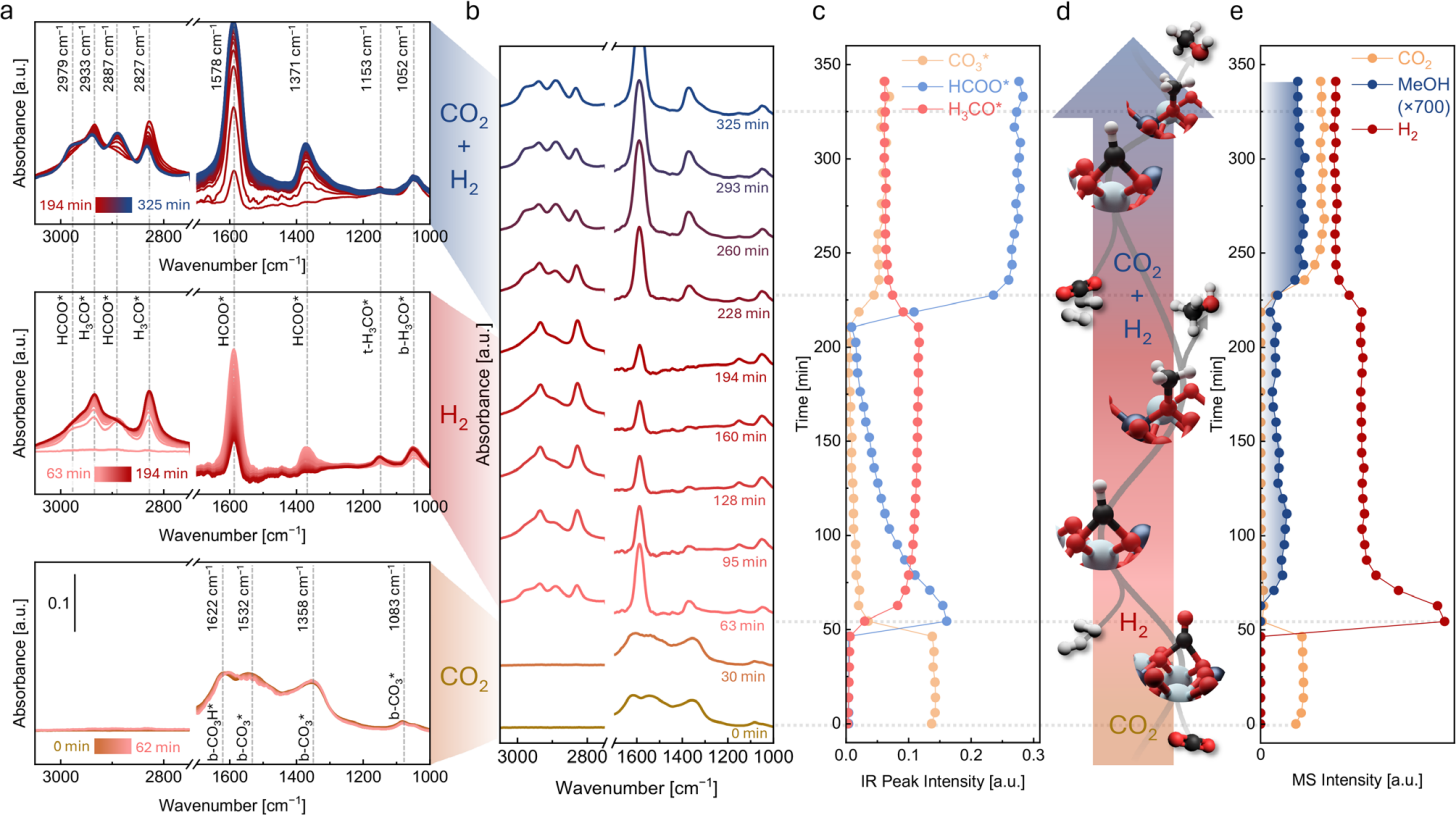
Operando DRIFTS measurement on 30ZnHfO*_x_*. (a) Evolution of the IR spectra under CO_2_, H_2_, and CO_2_ hydrogenation (H_2_:CO_2_ = 3:1) atmospheres at 320°C and 20 bar. (b) Selected spectra collected during the operando DRIFTS experiment. (c) Evolution of CO_3_* (1532 cm^−1^), HCOO* (1578 cm^−1^), and H_3_CO* (2827 cm^−1^) species as a function of time. (d) Illustration of the evolution of the surface species under the different atmospheres during the operando DRIFTS experiment. (e) Off‐gas analysis by mass spectrometry during the operando DRIFTS experiment (using m/z = 31 for methanol, m/z = 2 for H_2_, m/z = 44 for CO_2_).

To study the reaction pathway of methanol formation on ZnHfO*_x_* catalysts, an operando DRIFTS experiment was conducted on 30ZnHfO*_x_*. Here, the gas flow to the reaction cell was altered sequentially from (i) CO_2_, to (ii) H_2_, and finally (iii) CO_2_ hydrogenation conditions (H_2_:CO_2_ ratio = 3:1 as in the fixed bed reactor experiments). In the first step of the experiment, 30ZnHfO*_x_* was kept under a continuous flow of CO_2_ and N_2_ (20 vol% CO_2_) at 20 bar and 320°C for 1 h. Under these conditions, bands due to bidentate carbonate (b‐CO_3_*) at 1083, 1358, and 1532 cm^−1^, and bidentate bicarbonate (b‐CO_3_H*) at 1622 cm^−1^ were detected (Figure 6a). After switching the gas to a mixture of H_2_ and N_2_ (80 vol% H_2_), the carbonate and bicarbonate species disappeared instantaneously, while a prominent band at 1578 cm^−1^ due to the ν(C = O) stretching vibration of formate (HCOO*), and less intense bands at 2979 cm^−1^ and at 2887 cm^−1^ due to respectively, the asymmetric stretching ν_as_(C–H) and symmetric stretching ν_s_(C–H) vibrations of formate appeared (Figure 6b,c). With time (and with a decreasing concentration of CO_2_ from the previous step, see MS profiles presented in Figure 6e), the intensity of the formate bands slowly decreased while bands assigned to the asymmetric stretching ν_as_(C–H), 2933 cm^−1^, and symmetric stretching ν_s_(C–H), 2827 cm^−1^, vibrations of methoxy species (H_3_CO*) appeared and gradually increased. In addition, bands assigned to terminal (t‐H_3_CO*), 1153 cm^−1^, and bridged (b‐H_3_CO*), 1052 cm^−1^, methoxy species emerged as the formate species were gradually hydrogenated to methoxy species in H_2_. These observations provide strong evidence that on ZnHfO*_x_*‐based catalysts CO_2_ hydrogenation to methanol proceeds, similar to ZnZrO*_x_* [23, 25] and india‐based catalysts [72], via the formate‐methoxy pathway, and not via the CO/RWGS mediated pathway, which has been observed for example in Cu/Pd‐based methanol catalysts [73, 74]. In the final step of the operando DRIFTS experiment, the gas flow was switched to a mixture of H_2_, CO_2_, and N_2_ (3:1:1). As CO_2_ was introduced back into the cell, the surface of the catalyst was immediately re‐populated with formate species, while bands due to methoxy species decreased in intensity concomitantly (Figure 6b,c). The IR band intensities of the key reaction intermediates stabilized approximately 30 min after CO_2_ had been re‐introduced (i.e., as soon as the gas phase composition had stabilized) and remained constant for the remaining 1.5 h of the experiment. The off‐gas composition was analyzed by MS throughout the entire operando DRIFTS experiment (Figure 6e), showing the presence of small quantities of methanol in the off‐gas already when switching from CO_2_ to H_2_ (after approximately 60 min). The methanol concentration in the off‐gas gradually decreased as CO_2_ was purged out of the cell, and the surface intermediates were hydrogenated under H_2_ flow. When a CO_2_ hydrogenation atmosphere was introduced to the DRIFTS cell (after approximately 210 min), a steady methanol concentration was observed in the off‐gas. The same trends were observed in an in situ DRIFTS experiment for 20ZnHfO*_x_* (Figure S49).

**FIGURE 7:**
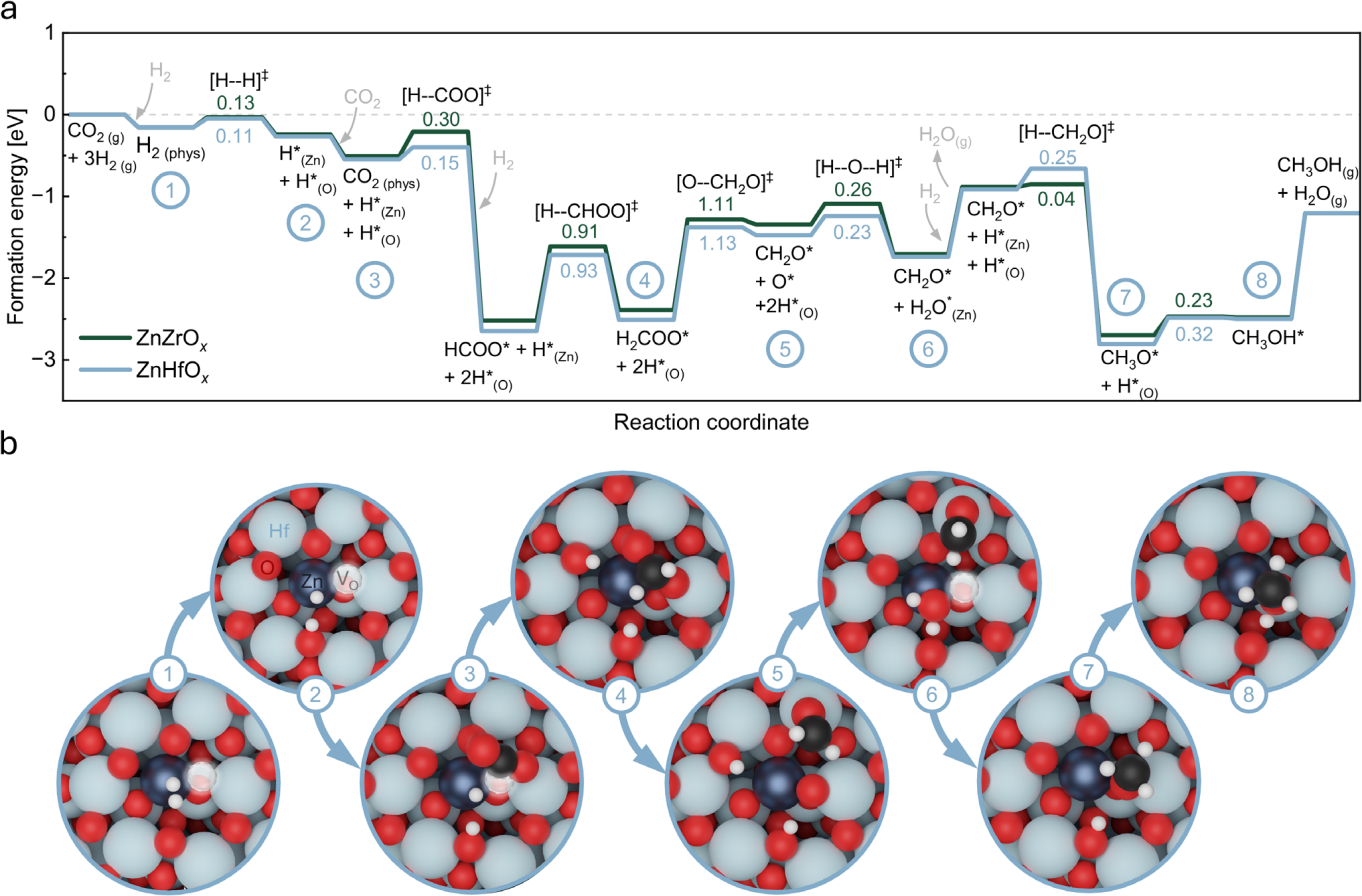
DFT potential energy profiles. (a) Potential energy diagrams for CO_2_ hydrogenation to methanol on ZnHfO*_x_* (blue) and ZnZrO*_x_* (green). All energies are referenced to the initial states, namely, gas‐phase CO_2_, H_2_, and H_2_O, and the corresponding clean catalyst surfaces. Lateral interactions between neighboring adsorbates are included. (b) Snapshots of representative surface states along the reaction coordinate on the ZnHfO*_x_* model. Hf atoms are shown in light blue, Zn in dark blue, O in red, C in black, and H in white. The oxygen vacancy is indicated by a transparent white sphere. Circled numbers correspond to states in panel (a).

To rationalize the experimental observations, we performed DFT calculations on ZnHfO_x_ to determine the potential energy profile for CO_2_ hydrogenation to methanol (Figure 7a) following the formate pathway (based on our DRIFTS measurements). For comparison, the same reaction pathway was also evaluated on the reference ZnZrO*_x_* system.

The reaction is initiated with a heterolytic dissociation of H_2_ at the dopant (Zn) site, yielding one H* species bound to the Zn atom and a second H* bonded to a neighboring lattice oxygen site (state 2 in Figure 7a). In parallel, CO_2_ physisorbs near the dopant Zn site (state 3). The subsequent hydrogenation of CO_2_ to a surface formate (HCOO*) species proceeds by the reaction of physiosorbed CO_2_ with adsorbed H*, via a very low energy barrier of 0.15 eV on ZnHfO*_x_*. This finding is consistent with the operando DRIFTS results, which show that formate species are formed instantaneously.

Along the formate pathway, the highest energy barriers correspond to the hydrogenation of HCOO* to H_2_COO* (0.93 eV) and the subsequent C–O bond cleavage to form CH_2_O* (1.19 eV). Oxygen vacancies play a crucial role in this step by stabilizing the resulting O* fragment and facilitating its transformation into H_2_O through its reaction with surface hydrogen species. The mechanism differs slightly from the route proceeding through the formation of H_2_COOH* from H_2_COO*, as proposed previously by Araújo et al. [35]. and Cheula et al. [75]. on ZnZrO*_x_*. Once CH_2_O* is formed, it is hydrogenated to CH_3_O*, requiring its diffusion to the oxygen vacancy site of the Zn–V_O_–Hf motif. Finally, CH_3_O* is hydrogenated to CH_3_OH* by a neighboring surface H*, which desorbs from the surface, completing the catalytic cycle. A direct comparison between the formate pathways proceeding on ZnHfO*_x_* and ZnZrO*_x_* reveals that both catalysts exhibit very similar overall energy profiles. The only differences worth mentioning are a slightly lower energy barrier for formate formation on ZnHfO*_x_* (0.15 eV) compared to ZnZrO*_x_* (0.30 eV), as well as a slightly stronger stabilization of most reaction intermediates on ZnHfO*_x_* by 0–0.13 eV (Figure S50). These differences are consistent with the experimentally observed high intrinsic methanol formation rates of ZnHfO*_x_* catalysts and support the formate‐methoxy pathway and the proposed involvement of oxygen vacancies in the catalytic cycle for methanol synthesis.

### 2.7 CO_2_ Activation of ZnHfO*_x_*

**FIGURE 8:**
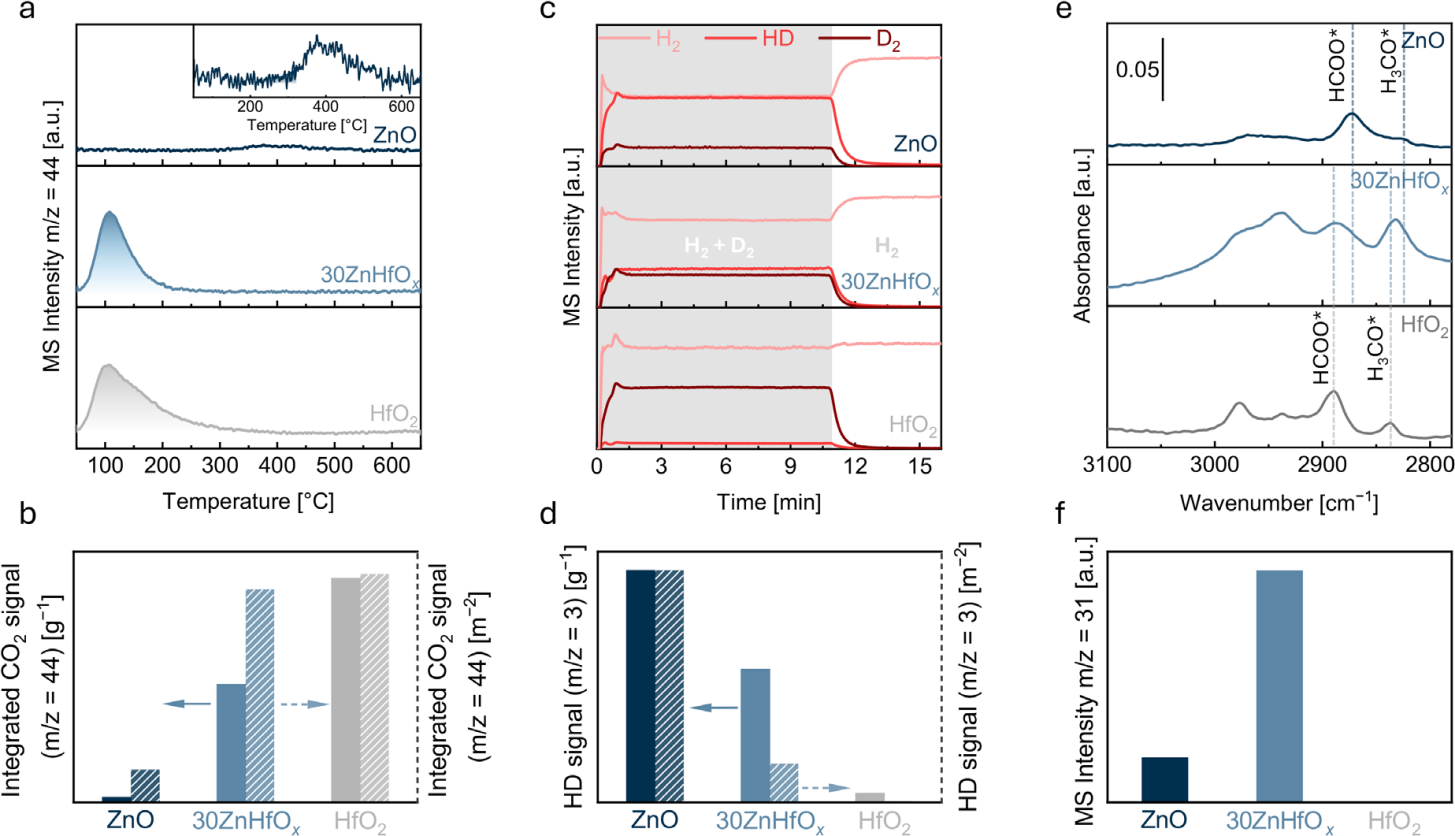
Surface chemistry analysis of 30ZnHfO*_x_*, HfO_2_, and ZnO. (a) CO_2_ TPD profiles of ZnO, 30ZnHfO*_x_*, and HfO_2_ over the temperature range 50°C–650°C. (b) Integrated CO_2_ TPD profiles of ZnO, 30ZnHfO*_x_*, and HfO_2_ normalized by mass or surface area. (c) MS signals (m/z = 2 for H_2_, m/z = 3 for HD, and m/z = 4 for D_2_) from H_2_‐D_2_ exchange experiments of ZnO, 30ZnHfO*_x_*, and HfO_2_ at 320°C, using a H_2_ concentration of 10 vol% and a D_2_ concentration of 5 vol% and total flow rate of 40 mL min^−1^. (d) HD signal intensity normalized by mass and surface area for ZnO, 30ZnHfO*_x_*, and HfO_2_. (e) Operando DRIFTS spectra of ZnO, 30ZnHfO*_x_*, and HfO_2_ after 90 min at 20 bar, 320°C, H_2_:CO_2_:N_2_ = 3:1:1, and 24 L(g_cat_h)^−1^. (f) Methanol signal (m/z  =  31) recorded by MS during operando DRIFTS measurements of ZnO, 30ZnHfO*_x_*, HfO_2_ (after 90 min).

CO_2_ temperature‐programmed desorption (TPD) experiments were conducted to probe the basicity of 30ZnHfO*_x_*, assess its capability to activate CO_2_, and contrast it to the pure oxides ZnO and HfO_2_ (Figure 8a). Both 30ZnHfO*_x_* and HfO_2_ exhibited one major CO_2_ desorption peak at approximately 120°C, while ZnO featured only a very small desorption peak at approximately 380°C. The desorption peak of HfO_2_ extended to approximately 350°C compared to 250°C for 30ZnHfO*_x_*, indicating the presence of stronger basic sites on monoclinic HfO_2_. Integrating the CO_2_ desorption peaks showed that HfO_2_ possessed the highest CO_2_ binding capacity per gram of material, followed by 30ZnHfO*_x_*, whereas the amount of CO_2_ desorbed on ZnO was very small (Figure 8b). When normalizing the CO_2_ desorption peak areas by the respective surface areas of the materials (given in Table S1), 30ZnHfO*_x_* and HfO_2_ exhibited a similar binding capacity per m^2^ of material, while the quantity of CO_2_ desorbed on ZnO is very small.

Additional CO_2_ TPD experiments were performed on ZnHfO*_x_* catalysts containing 10, 20, 50, 70, and 90 mol% Zn (Figure S51). When normalizing the CO_2_ TPD peak area by the respective surface areas, the amount of CO_2_ desorbed increased with increasing Zn content up to 30ZnHfO*_x_*, which was likely due to the increasing quantity of oxygen vacancies with increasing Zn doping. However, for Zn contents > 30 mol%, the CO_2_ sorption capacity of ZnHfO*_x_* normalized by surface area, decreased substantially, which is attributed to the segregation of hexagonal ZnO which has a lower CO_2_ sorption capacity. These results are in good agreement with the declining CO_2_ conversion observed for ZnHfO*_x_* catalysts with increasing ZnO segregation (Figure 4a). In fact, when correlating the CO_2_ binding capacity of the ZnHfO*_x_* catalysts with their CO_2_ conversion and methanol yield, a linear trend is observed (Figure S52), with 10ZnHfO*_x_* being the only outlier, potentially due to different properties of the active site in Zn‐doped monoclinic HfO_2_ (for a low Zn content) compared to Zn‐doped cubic HfO_2_.

### 2.8 H_2_ Activation of ZnHfO*_x_*

H_2_ and D_2_ (deuterium) exchange experiments were carried out at ambient pressure at 320°C to probe the ability of ZnO, 30ZnHfO*_x_*, and HfO_2_ to activate H_2_, by analyzing the off‐gas for the exchange product HD with MS (Figure 8c). The highest amount of HD was observed for ZnO, followed by 30ZnHfO*_x_*, whereas HfO_2_ produced only very little HD. When normalizing the HD signal intensity to the surface area of the respective materials, ZnO remained the most active material for HD formation, while HD formation per m^2^ of material was very low for HfO_2_, confirming the importance of Zn in ZnHfO*_x_* to activate H_2_. Surprisingly, 90ZnHfO*_x_* yielded less HD than 30ZnHfO*_x_* (Figure S53)_,_ which we attribute to the surface modification of the segregated ZnO particles by HfO*_x_* clusters. However, the surface area specific HD production of 90ZnHfO*_x_* still exceeded that of pure HfO_2_ by over 400%. In addition to monoclinic HfO_2_, also tetragonal and amorphous HfO_2_ were investigated for their ability to activate H_2_ in H_2_‐D_2_ experiments; it was found that all HfO_2_ polymorphs had similar, very low HD formation rates (Figure S54).

DFT calculations evaluating the role of the dopant Zn in H_2_ activation reveal that H_2_ activation is facilitated by both Zn incorporation and the presence of an adjacent oxygen vacancy. A comparative DFT analysis of four representative HfO_2_ surface models—pristine HfO_2_, a HfO_2_ surface with an oxygen vacancy, Zn‐doped HfO_2_ without an oxygen vacancy (ZnHfO_2_), and Zn‐doped HfO_2_ containing an oxygen vacancy adjacent to Zn (ZnHfO*_x_*)—shows that all surfaces exhibit comparable energetics for an H_2_ molecule to adsorb. However, despite this similarity, only the ZnHfO_x_ surface favors H_2_ activation via a heterolytic dissociation pathway (exothermic reaction energy of −0.11 eV and a low activation barrier of 0.10 eV). Here, the oxygen vacancy plays a key role in stabilizing the hydride–Zn bond formed upon heterolytic dissociation. In contrast, in the absence of an oxygen vacancy, that is, the ZnHfO_2_ surface, H_2_ activation can only proceed via a homolytic pathway, which does not yield hydride species and makes the subsequent formation of formate species energetically unfavorable (Figure S55a,c). These findings highlight the importance of the proximity of the oxygen vacancy and the Zn site in the active motif for enabling hydrogen activation and the subsequent hydride transfer for formate formation. Additionally, the presence of an oxygen vacancy enables the cleaving of H_2_COO* into CH_2_O* and O*.

### 2.9 ZnHfO*_x_* Active Site for Methanol Formation

To investigate the formation of reaction intermediates on ZnO and HfO_2_ under CO_2_ hydrogenation conditions (20 bar, 320°C, H_2_:CO_2_:N_2_ = 3:1:1) and contrast them to 30ZnHfO*_x_*, both individual oxides were analyzed by operando DRIFTS (Figure 8e). Under CO_2_ hydrogenation conditions, ZnO exhibited a band at ∼ 2873 cm^−1^ due to formate, which is in agreement with studies reporting on the adsorption of formic acid onto ZnO [76]. Yet, only very weak IR bands due to methoxy species were observed for ZnO, which can be rationalized by the poor ability of pure ZnO to activate CO_2_. Surprisingly, HfO_2_ revealed vibrational bands due to both formate and methoxy species under CO_2_ hydrogenation conditions, although no methanol was detected in the off‐gas by MS (Figure 8f). This finding may indicate that either the turnover of the reaction intermediates was very slow, or that surface methoxy species could not be hydrogenated to methanol on HfO_2_ in the absence of Zn, concordant with the rather low ability of pure HfO_2_ to activate H_2_, as shown in the H_2_‐D_2_ exchange experiments and DFT calculations. The intensity of the formate and methoxy bands on HfO_2_ was significantly lower compared to 30ZnHfO*_x_*, implying a lower population of reaction intermediates on the surface of HfO_2_ under reaction conditions (Figure 8e). Further, we observed a small bathochromic shift (4–5 cm^−1^) for the formate and methoxy bands on 30ZnHfO*_x_* compared to HfO_2_, positioning them in between the respective wavenumbers for the individual oxides, which indicates that the reaction intermediates were stabilized by an active motif that involves both Zn and Hf. Hence, combining these findings with operando UV‐Vis spectroscopy experiments and computational analysis, we postulate that Zn–V_O_–Hf sites are the active motifs for methanol formation in ZnHfO*_x_* catalysts.

**FIGURE 9:**
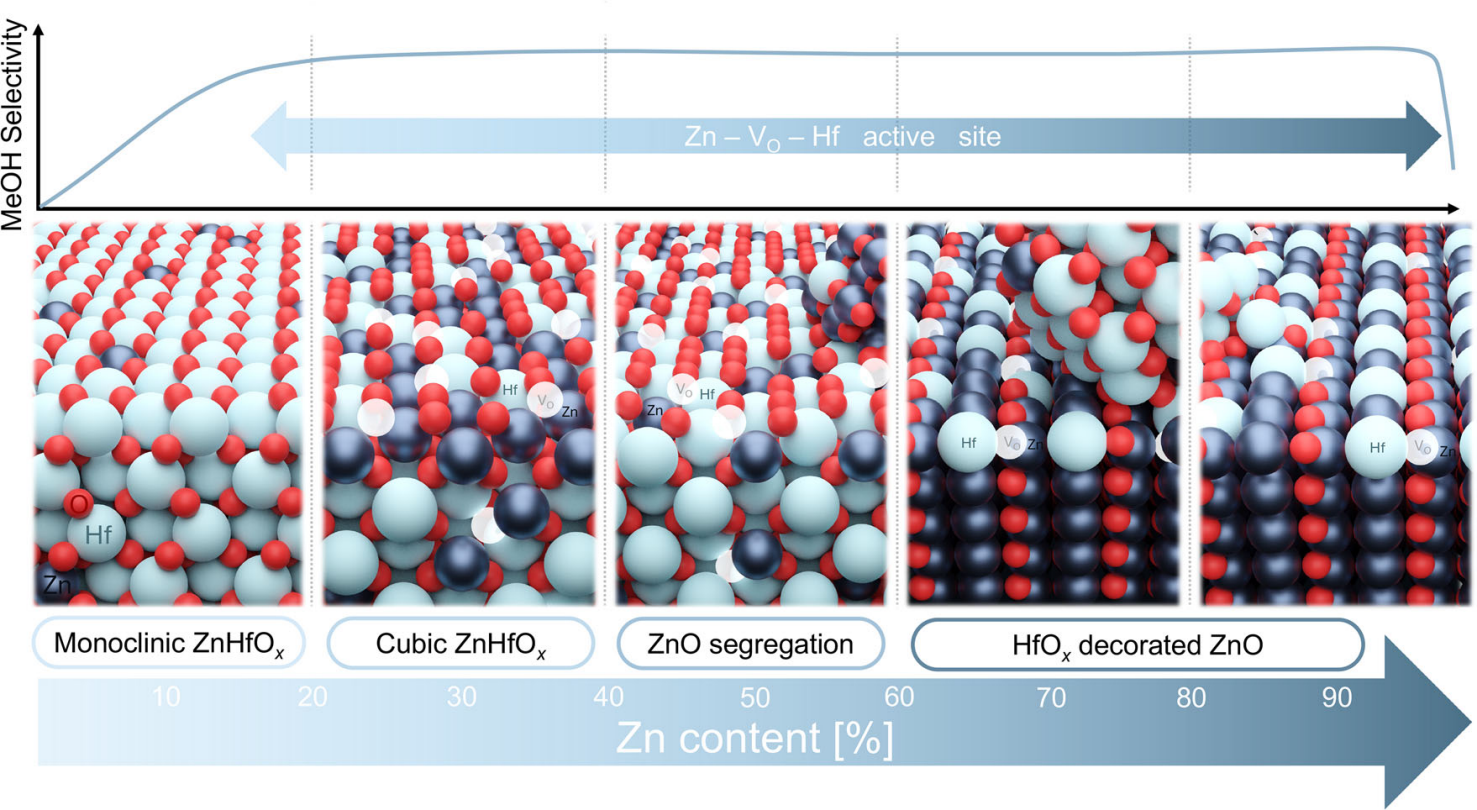
Illustration of the structural evolution of ZnHfO*_x_* catalysts and their active site for Zn contents ranging from 10 to 95 mol%. Dark blue spheres represent Zn, light blue spheres Hf, red spheres O atoms, and translucent white spheres represent oxygen vacancies.

At very high Zn contents (90ZnHfO*_x_*), the IR signatures observed by in situ DRIFTS are very similar to that of 20ZnHfO*_x_* (with regard to wavenumber position and dynamics of surface adsorbates/reaction intermediates), demonstrating that on both materials, similar active sites for methanol formation are at play (Figure S56). In addition, the apparent activation energies for methanol formation are in the range of 77–83 kJ/mol for the ZnHfO*_x_* catalysts with Zn contents in the broad range of 20–98 mol% Zn, that is, the activation energy is independent of the Hf content, providing further evidence that for both low and high Zn content catalysts methanol synthesis proceeds over similar active sites (Figures S57 and S58). In contrast, pure ZnO has an apparent activation energy for methanol formation of approximately 40 kJ/mol. A detailed discussion of these spectroscopic and kinetic results is included in the supplementary information. Taken together, we infer that Zn–V_O_–Hf motifs constitute the main active sites in both catalyst configurations, that is, ZnHfO*_x_* solid solutions as well as ZnO surfaces decorated by HfO*_x_* clusters (and/or single Hf atoms), explaining in turn the high methanol selectivity of ZnHfO*_x_* over a wide range of Zn contents (Figure 9). These HfO*_x_* clusters (and/or single Hf atoms) are stable under harsh CO_2_ hydrogenation conditions, as evidenced by catalytic testing (Figure S25) and post catalytic HR‐STEM imaging (Figure S59). Based on the N_2_ physisorption‐determined surfaces areas (Figure S60) and using models of hexagonal ZnO, (0001) and (1010) facets, a Hf loading as low as ∼0.2 and ∼0.5 mol% would yield a surface coverage of, respectively, 25% and 50% of the surface Zn atoms (Figure S61).

## 3 Conclusion

In this work, ZnHfO*_x_* mixed oxides are introduced as a novel catalyst family for the CO_2_ hydrogenation to methanol. The Zn content (Zn/(Zn+Hf)) was varied in a broad range of 10 to 99.5 mol%. For Zn contents ≤ 35 mol%, Zn was doped into the structure of HfO_2_, yielding (only) a solid solution‐type ZnHfO_x_ phase, as confirmed by XRD. While TEM analysis revealed a Zn enrichment at the surface of the catalyst with 30 mol% Zn, small ZnO clusters started to form in 35ZnHfO*_x_* and the segregation of bulk ZnO occurred for Zn contents exceeding 35 mol%. Although XRD analysis suggests that no significant amounts of Hf were integrated into the bulk structure of segregated, hexagonal ZnO, TEM, UV‐Vis, and Raman spectroscopy indicate the presence of HfO*_x_* clusters on its surface.

The best performing catalyst 20ZnHfO*_x_* outperformed the intrinsic, mol specific methanol formation rate of the reference 20ZnZrO*_x_* by 15%. For Zn contents > 20 mol%, ZnHfO*_x_* catalysts outperformed the intrinsic methanol formation rate of ZnZrO*_x_* by even larger margins (> 36%) due to their consistently high methanol selectivity (> 70% at 20 bar, 320°C and 24 L(g_cat_h)^−1^) up to 99.5ZnHfO*_x_*.

Operando UV‐Vis spectroscopy provides evidence that the presence of oxygen vacancies are crucial for the reaction to proceed, while operando DRIFTS and complementary surface chemistry analysis revealed that both Zn and Hf participate in the CO_2_ hydrogenation mechanism, suggesting that Zn–V_O_–Hf motifs are the active sites for methanol formation via the formate‐methoxy pathway. DFT calculations support this assignment by confirming that (i) formate formation on ZnHfO_x_ is associated with a low activation barrier, (ii) hydrogen dissociation is strongly favored at Zn sites that are adjacent to oxygen vacancies and (iii) oxygen vacancy sites facilitate the formation of a Zn‐hydride motif, following H_2_ dissociation, as well as C‐O bond cleavage in H_2_COO* leading to CH_2_O* and O* whereby O* is bound to an oxygen vacancy site. The active Zn–V_O_–Hf motifs are present in the solid‐solution type ZnHfO_x_ phase for Zn contents ≤ 35 mol% and at the surface of segregated ZnO particles that are decorated with Hf/HfO_x_ clusters for Zn contents > 35 mol%, rationalizing the high methanol selectivity over a wide range of Zn contents. The HfO*_x_* clusters on ZnO are generated due to the strong interactions between ZnO and HfO_2_, as the migration of Hf onto ZnO was observed even for a calcined physical mixture of the individual oxides. Such strong interactions are absent for physical mixtures of ZnO and ZrO_2_, explaining the superior performance of ZnHfO*_x_* over ZnZrO*_x_* at high Zn contents. This finding is particularly important considering the high cost of Hf (and Zr) when compared to Zn. The ZnHfO*_x_* catalyst introduced here presents a very promising CO_2_ hydrogenation catalyst, the performance of which may be increased further by alternative synthesis approaches to optimize Hf usage and/or metal promotion.

## Author Contributions

**Alexander Oing**: investigation, conceptualization, methodology, data curation, writing – original draft. **Diana Piankova**: investigation, methodology, data curation, writing – review and editing. **Jean C. Villa‐Arpi**: investigation, writing – review and editing, data curation, methodology. **Muhammad Helmi Risansyauqi**: investigation, data curation, writing – review and editing. **Hector Prats**: methodology, investigation, data curation, writing – review and editing, funding acquisition. **Felix Donat**: investigation, writing – review and editing, supervision. **Paula M. Abdala**: supervision, data curation, investigation, conceptualization, writing – review and editing. **Aleix Comas‐Vives**: writing – review and editing, supervision, conceptualization, funding acquisition. **Christoph R. Müller**: conceptualization, writing – review and editing, supervision, funding acquisition.

## Conflicts of Interest

The authors declare no conflicts of interest.

## Supporting information




**Supporting File**: anie73201‐sup‐0001‐SuppMat.pdf.

## Data Availability

The data generated in this study are publicly available in the Zenodo database under the digital object identifier (DOI) https://doi.org/10.5281/zenodo.20541315.
