# Supplementary material for: The Role of Zn–Hf Site Proximity and Oxygen Vacancies for Methanol Formation Over ZnHfO x Catalysts Under CO2 Hydrogenation Conditions
Source: Angew Chem Int Ed Engl. 2026 Jun 30;65(34):e8850754. doi: 10.1002/anie.8850754 (PMC13480701; doi:10.1002/anie.8850754)
Supplement: Supplementary file 1 [file ANIE-65-e8850754-s001.pdf]

Supporting Information

The Role of Zn–Hf Site Proximity and Oxygen Vacancies for Methanol Formation over $ZnHfO_x$ Catalysts under CO_2 Hydrogenation Conditions

*Alexander Oing[a], Diana Pankova[a], Jean C. Villa Arpi[b], Muhammad Helmi Risansyauqi[b], Hector Prats[b, c], Felix Donat[a], Paula M. Abdala[a], Aleix Comas-Vives[b], Christoph R. Müller[a]**

[a]Department of Mechanical and Process Engineering, ETH Zurich, Zurich, Switzerland

[b]Institute of Materials Chemistry, TU Wien, Vienna, Austria

[c] Departament de Ciència de Materials i Química Física & Institut de Química Teòrica i Computacional (IQTCUB), Universitat de Barcelona, Barcelona, Spain

**Corresponding Author: muelchri@ethz.ch*

Table of Contents

Materials and Methods *3*

Supplementary Discussion *10*

Supplementary Figures *15*

Supplementary Tables *87*

Supplementary References *91*

Materials and Methods

Synthesis

$ZnHfO_x$ catalysts were synthesized by co-precipitation. A total of 3.12 mmol of metal chlorides ($ZnCl_2$ (Thermo Scientific, 98 %) and $HfCl_4$ (Sigma-Aldrich, 98 %)) were dissolved in the desired Zn/Hf ratio in 50 mL of deionized water and heated to 70 °C. Separately, the precipitant was prepared by mixing aqueous ammonia (35 wt.%, Fisher Scientific) with deionized water in a volumetric ratio of 1:2. The precipitant was added dropwise to the metal chloride solution under continuous magnetic stirring at 70 °C until a pH of 7 was reached. A pH of 7 was chosen for the synthesis to ensure that both metals precipitate, while simultaneously ensuring a crystallization of the material during calcination (which becomes negatively affected at pH $\gg$ 7). The slurry was kept under continuous stirring at 70 °C for 2 h before washing the product five times in deionized water. Finally, the gel was dried in an oven at 80 °C overnight and calcined in a muffle furnace at 580 °C for 3 h (5 K min^{-1} ramp) to crystallize the powder sample. The calcination temperature was set to 580 °C to ensure crystallinity of the $ZnHfO_x$ catalysts while limiting the influence of sintering that may compromise the catalyst's surface area at higher calcination temperatures. The same precipitation procedure was carried out to obtain the respective oxides ZnO and HfO_2.

$ZnZrO_x$ catalysts were synthesized according to the co-precipitation procedure reported by Wang *et al.*[1] A total of 15.55 mmol of metal nitrates ($Zn(NO_3)_2{\cdot}6H_2O$ (Thermo Scientific, 98 %) and $Zr(NO_3)_4{\cdot}5H_2O$ (Carl Roth, ≥ 98 %)) were dissolved in the desired Zn/Hf ratio in 100 mL of deionized water and heated to 70 °C. The precipitant was prepared by dissolving 3.06 g of $(NH_4)_2CO_3$ (Thermo Scientific, analysis grade) in 100 mL of deionized water. The precipitant was then added dropwise to the metal nitrate solution at 70 °C under continuous magnetic stirring and kept at these conditions for 2 h. The product was then washed five times with deionized water, dried at 80 °C and calcined in a muffle furnace at 500 °C for 3 h (5 K min^{-1} ramp).

$ZnHfO_{x\text{-IWI}}$ catalysts were prepared by incipient wetness impregnation. To impregnate 300 mg of HfO_2, the respective amounts of $Zn(NO_3)_2{\cdot}6H_2O$ (Thermo Scientific, 98 %) were dissolved in 150 µL of deionized water to reach 20 and 30 mol%. Due to the relatively small pore volume of the synthesized HfO_2 support material, Zn nitrate solution was added in several steps as the point of incipient wetness was already reached after adding approximately 30 µL. The material was mixed and dried between each impregnation iteration. The impregnated support was then dried at 80 °C overnight and calcined in a muffle furnace at 580 °C for 3 h (5 K min^{-1} ramp). $ZnHfO_{x\text{-PM}}$ and $ZnZrO_{x\text{-PM}}$ catalysts were synthesized

by grinding the respective oxides in the desired ratio in a mortar for 10 min. The ground materials were then calcined in a muffle furnace at 580 °C for 3 h (5 K min^{-1} ramp).

Characterization

X-ray diffraction: The crystal structure of the materials was analyzed with a PANalytical Empyrean X-ray powder diffractometer equipped with an X'Celerator Scientific ultrafast line detector and Bragg-Brentano high density incident beam optics. The diffraction patterns were collected for 1 – 3 hours in a 2Θ range from 10 ° to 90 ° with a step size of 0.016 ° using Cu Kα radiation (45 kV, 40 mA). All materials were prepared as finely ground powders on silicon wafer, zero-background sample holders. Rietveld refinements were carried out using the PCR editor within FullProf Suite. Cif files obtained from the inorganic crystal structure database (ICSD) were used as crystallographic data input files for the calculations. The peaks were assumed to be of a pseudo-Voigt shape, and the background was subtracted manually.

Diffuse reflectance UV-Vis spectroscopy: Diffuse reflectance UV-Vis spectroscopy (DRUVS) was conducted with a Perkin Elmer Lambda 750 spectrophotometer in the wavelength range from 172 – 850 nm and $BaSO_4$ powder as a background. For *operando* DRUVS measurements, 50 mg of finely ground catalyst was spread evenly on a bed of quartz wool in a Silcolloy coated Harrick Praying Mantis cell. The *operando* measurements were carried out at 20 bar and a total flow rate of 20 mL min^{-1} and the off-gas was analyzed by a mass spectrometer (Pfeiffer ThermoStar GSD 320). Tauc plots were constructed according to Makula *et al.*[2] and the band gap E_g was calculated using the Tauc method and the following relationship:

$$(F(R_\infty) \cdot h\nu)^{\frac{1}{\gamma}} = B(\hbar\nu - E_g) \quad (1)$$

where $F(R_\infty)$ corresponds to the UV-Vis spectrum in Kubelka-Munk units, B is a constant, and h and v denote the Planck constant and photon's frequency, respectively. Here, γ was set to 2 due to the indirect and to 0.5 due to the direct electronic transitions for the photon absorption in HfO_2 and ZnO, respectively. E_g was then determined by constructing Tauc plots with $(F(R_\infty)\ h\nu)^{1/\gamma}$ on the ordinate and $h\nu$ on the abscissa. The linear section of the HfO_2 absorption was fitted with a linear function and its intersection with the abscissa corresponds to the band gap energy E_g.

Raman spectroscopy: Structural surface analysis was carried out with a Thermo Fisher DXR 2 Raman spectrometer equipped with a 532 nm excitation laser and a fifty-fold magnification objective, resulting in estimated spot sizes of 0.1 μm in diameter. The Raman spectra were recorded in a range from 120 – 3500 cm^{-1} with a resolution of 900 lines/mm. All plotted spectra were an average of a collected map of 100 individual Raman scans. For sample preparation, the materials were pressed into 3 mm pellets using a hand pelletizer to achieve a homogeneous surface for characterization.

Diffuse reflectance infrared Fourier transform spectroscopy: Diffuse reflectance infrared Fourier transform spectroscopy (DRIFTS) was performed using a Nicolet 6700 FT-IR. Each spectrum was calculated as an average of 64 individual scans and the spectra were acquired utilizing a mercury cadmium telluride detector cooled with liquid N_2 in a spectral resolution of 4 cm^{-1} from 650 – 4000 cm^{-1}. *Operando* experiments were carried out in a Silcolloy coated Harrick Praying Mantis cell with 100 mg of catalyst at 320 °C, 20 bar, and a total flow rate of 40 mL min^{-1}. The samples were ground to a fine powder and evenly distributed on a bed of quartz wool. The samples were kept in N_2 at 320 °C for 1 h before switching to the experimental conditions. An IR spectrum of the sample at 320 °C in N_2 was used as a background for the differential spectra throughout the *operando* experiments. The off-gas was simultaneously analyzed by a mass spectrometer (MKS Cirrus 3) and the individual ion currents were normalized by the total pressure in the instrument.

Electron microscopy: Scanning/Transmission electron microscopy (S/TEM) studies were performed on a double Cs-corrected transmission electron microscope, the JEM-ARM300F Grand ARM "Vortex," operated at 300 kV and equipped with 2 large-area SDD Energy-dispersive X-ray (EDX) detectors with a 100 mm^2 active area. The camera length settings for recording High-angle annular dark-field (HAADF)-STEM images were set to cover a collection angle range of 54-220 mrad. The specimens were prepared by directly dipping the Cu grids coated with holey carbon into the powder of the materials (exposed to air). HAADF images were denoised using a non-linear filtering algorithm.[3]

Temperature programmed desorption: Temperature programmed desorption (TPD) experiments were conducted in a Micromeritics AutoChem 2920 II equipped with a thermal conductivity detector (TCD) and a mass spectrometer (MKS Cirrus 2) for off-gas analysis. The ion currents recorded by MS were normalized by the total pressure in the instrument. For each measurement, 100 mg of sample was

placed between two quartz wool plugs in the isothermal zone of a U-shaped quartz reactor. CO_2 TPD experiments were conducted by first heating the sample to 650 °C for 1 h in He. Subsequently, the sample was cooled to 50 °C and CO_2 was adsorbed on the sample by flowing 5 vol% CO_2 in He for 1 h. The quartz reactor was then purged for 2 hours in He before starting the TPD by ramping to 650 °C at 10 K min^{-1} and dwelling for 30 min. The total gas flow rate was 20 mL min^{-1} throughout the CO_2 TPD experiment.

H_2-D_2 isotope exchange experiments: H_2-D_2 exchange isotope experiments were performed in a tubular quartz reactor with an inner diameter of 5 mm and a total volume of approximately 9 mL. 100 mg of sample was placed inside the isothermal zone of the reactor on a plug of quartz wool. The samples were heated to 320 °C in 5 vol% H_2 at ambient pressure and 40 mL min^{-1} for 30 min, before flowing H_2 (5 vol%) and D_2 (2.5 vol%) simultaneously for 10 min. The off-gas was analyzed by a mass spectrometer (Pfeiffer OmniStar GSD 350) to assess the formation of the HD intermediate, and the individual ion currents were normalized by the total pressure in the instrument.

N_2 physisorption analysis: The specific surface areas of all catalysts and reference materials were determined by N_2 physisorption in an Anton Paar NOVA 800 instrument. 100 – 200 mg of sample was pretreated by heating to 300 °C for 3 h in vacuum prior to N_2 adsorption at liquid N_2 temperature (−196 °C). The specific surface areas were then calculated by the Brunauer-Emmett-Teller (BET) method, and the pore volume and pore size volume and distribution were determined by the Barrett-Joyner-Halenda (BJH) method. The complete N_2 ad- and desorption isotherms, as well as the BET fits can be found in the supplementary information (Figure S62 – Figure S64).

X-Ray photoelectron spectroscopy: XPS measurements were conducted on a Thermo Scientific Sigma 2 spectrometer equipped with a hemispherical Alpha 110 analyzer in fixed transmission mode. The pressure inside the main chamber was 6 – 7 × 10^{-8} mbar during measurements and the samples were analyzed in large area XPS mode at an emission angle of 0° with respect to the normal vector of the sample (50° between the X-ray source and analyzer angle). The instrument was calibrated by measuring sputter cleaned references (Au 4f7/2 at 83.95 ± 0.1 eV, Ag 3d5/2 at 368.22 ± 0.1 eV, and Cu 2p3/2 at 932.62 ± 0.1 eV). The measurements were conducted using monochromatic Mg X-rays (200 W) to inhibit interferences between Zn Auger electrons and the O 1s spectra. High-resolution spectra were recorded

with a pass energy of 20 eV, a step size of 0.1 eV, and a dwell time of 240 ms per step. Survey scans were carried out at a pass energy of 50 eV and at a step size of 1 eV (50 ms dwell time) before and after high-resolution scans to check for X-ray damages. The peaks in the O 1s spectra were shifted by approximately 10 eV due to charging effects at the surface of the samples and the shift was corrected using references from literature.[4–6] The peak positions were not used for scientific interpretation and analysis due to the observed shift in binding energy.

Performance evaluation: The catalysts were evaluated catalytically in an automated fixed bed reactor setup (Microactivity Effi, PID Eng&Tech). For their catalytic evaluation, 50 mg of $ZnHfO_x$ was sieved to a size fraction of 125 – 200 μm and diluted with 400 mg of quartz chips (sieved to 200 – 300 μm) that are inert under reaction conditions. The diluted catalyst was sandwiched between two plugs of quartz wool and placed in a stainless-steel tubular reactor (inner diameter 9 mm). The catalyst was pretreated in N_2 at 300 °C for 60 min before switching to reaction conditions (typically 20 bar, H_2:CO_2:N_2 = 3:1:1, GHSV = 24 $L(g_{cat}h)^{-1}$). The off-gas was analyzed by a two-channel gas chromatograph (Agilent N6890) equipped with a flame ionization detector (FID) for quantification of C-based products and a thermal conductivity detector (TCD) for the quantification of H_2 and N_2. The N_2 in the feed functioned as an internal standard to determine the total molar flow at the outlet under the assumption of the ideal gas law. First, the total molar inlet flow was calculated from:

$$\dot{n}_{\mathrm{in}} = \frac{\dot{V}_{\mathrm{tot}} p_{\mathrm{cal}}}{T_{\mathrm{cal}} R} \quad (2)$$

Where R corresponds to the ideal gas constant, $\dot{V}_{tot}$ the total volumetric flow rate into the reactor, and p_{cal} and T_{cal} are the pressure and temperature, respectively, for which the mass flow controllers were calibrated. Since the molar flow of N_2 remains constant, the total molar flow at the outlet $\dot{n}_{tot,out}$, as well as the individual molar flows $\dot{n}_{i,out}$ for the reaction products i were determined with the respective concentrations x_i by:

$$\dot{n}_{\mathrm{tot,out}} = \dot{n}_{\mathrm{in}} \frac{x_{\mathrm{N_2,in}}}{x_{\mathrm{N_2,out}}} \quad (3)$$

$$\dot{n}_{i,\text{out}} = x_{i,\text{out}}\dot{n}_{\text{tot,out}} \quad (4)$$

The CO_2 conversion X_{CO2} and carbon-based product selectivities S_i were then calculated using the following equations:

$$X_{CO_2} = 1 - \frac{\dot{n}_{\text{CO}_2\text{,in}}}{\dot{n}_{\text{CO}_2\text{,out}} + \sum_{\text{C-Products}} N_{i,\text{C}}\dot{n}_{i,\text{out}}} \quad (5)$$

$$S_i = \frac{N_{i,\text{C}}\dot{n}_{i,\text{out}}}{\sum_{\text{C-Products}} N_{i,\text{C}}\dot{n}_{i,\text{out}}} \quad (6)$$

Where $N_{i,\text{C}}$ denotes the number of C atoms of the respective product molecule i. To assess the validity of the catalytic performance results, the carbon balance was calculated with the following formula:

$$C_{Bal} = \frac{\dot{n}_{\text{CO}_2\text{,out}} + \sum_{\text{C-Products}} N_{i,\text{C}}\dot{n}_{i,\text{out}}}{\dot{n}_{\text{CO}_2\text{,in}}} \quad (7)$$

Finally, the intrinsic methanol formation rates were calculated by:

$$r_{\text{MeOH,cat}} = \frac{\dot{n}_{\text{MeOH,out}}M_{\text{cat}}}{m_{\text{cat}}} \quad (8)$$

where m_{cat} is the mass and M_{cat} the molar mass of the respective $ZnZrO_x$ or $ZnHfO_x$ catalysts.

Density functional theory (DFT) calculations:

All DFT calculations were performed with the VASP package (version 6.4.2)[7] using the Perdew-Burke-Ernzerhof (PBE) exchange-correlation functional.[8] A dispersion correction was included using the DFT-D3 method[9] with the Becke-Johnson damping function.[10] A plane-wave basis set with a kinetic energy cutoff of 520 eV was employed to describe the valence electrons for the slab and bulk models, while the projector augmented wave (PAW) method[11,12] was used to treat core electrons. The electronic self-

consistency tolerance was set to 10^{-5} eV and 10^{-6} eV for the slab and bulk calculations, respectively. Ionic optimization was performed using the conjugate gradient method with a force convergence threshold of 0.01 eV/Å. All slab and bulk calculations were performed with a Γ-centered k-point mesh using a k-spacing of 1.0 and 0.66 $Å^{-1}$, respectively. Dipole corrections were applied for all slab calculations. The transition states (TS) were located using the ML-NEB module from CatLearn using a convergence criteria of uncertainty below 0.05 eV and a force threshold of 0.02 eV/Å.[13] Vibrational frequency analysis was performed for the transition state structures using a finite-difference method with a displacement of 0.015 Å. The structure was considered a transition state when it exhibited only one imaginary frequency along the reaction path.

Periodic boundary conditions were employed for all bulk and surface models used in this work. The monoclinic phases of HfO_2 and ZrO_2 were selected to model the structures, consistent with experimental observations indicating that at low Zn loading (10% mol Zn), a solid solution of $ZnHfO_x$ is formed with the monoclinic phase as the dominant bulk structure. The optimized bulk lattice constants of monoclinic HfO_2 and ZrO_2 are in good agreement with the experimental values, as shown in Table S2.

The (−111) surface, reported as the most thermodynamically stable surface of monoclinic HfO_2[14,15] and ZrO_2[16] was chosen for modeling the surface. The slab models of both structures were constructed by cleaving the corresponding bulk structure and consist of 2×2 supercells with three atomic layers, with the bottom layer fixed to the bulk positions. The Zn-doped slab models were created by exchanging an Hf or Zr atom with Zn and removing a neighbouring O to obtain a charge-neutral defect.

For modelling a high-Zn-loading catalyst structure, surface models were constructed by substituting a lattice Zn atom on the ZnO (1010) surface with Hf or Zr. The (1010) surface of ZnO was selected because it has been reported as the thermodynamically most stable non-polar surface of ZnO.[17] The surface was created by cleaving the relaxed bulk ZnO structure and consists of 3×2 supercells with 4 atomic layers, with the two bottom layers fixed to the bulk positions. All slab models include a vacuum region of 16 Å, which is sufficient to prevent spurious interactions between periodic images. All bulk and surface models are illustrated in Figure S65 and Figure S66.

Supplementary Discussion

Further discussion on the synthesis and characterization of $ZnHfO_x$ catalysts

The bulk crystal structure of $ZnHfO_x$ was also affected by the pH value during precipitation. For example, for $10ZnHfO_x$, the catalysts crystallized in a monoclinic HfO_2 phase for a pH in the range 2-7 (Figure S67). For pH values > 7, the diffraction patterns showed peak broadening indicative of a lower overall crystallinity of the materials; in addition, reflections, most likely attributed to the cubic or tetragonal phases of HfO_2, appear. A pH dependent bulk crystal structure of precipitation-derived ZrO_2 has been reported previously with ZrO_2 transitioning from a monoclinic to a tetragonal phase with increasing pH.[18]

Further performance evaluation of $ZnHfO_x$ catalysts

The performance of $ZnHfO_x$ was also investigated at temperatures of up to 400 °C during a long-term stability evaluation (potentially relevant in industrial applications to reach higher CO_2 conversions and to investigate the catalysts resistance against sintering). At 20 bar and 400 °C, the CO_2 conversion of $20ZnHfO_x$ increased to 12.5 % while the methanol selectivity decreased to 3 % (CO selectivity of 97 %). The most significant drop in methanol selectivity was observed between 340 and 360 °C (from 62 % to 36 %, respectively) (Figure S68 and Figure S69). The reactor was then cooled down, exposed to air, and reinstalled to investigate the stability of the activated catalyst. In the consecutive catalytic run, the CO_2 conversion and methanol selectivity reached their previous values, demonstrating its high catalytic stability (Figure S40). Moreover, no activation period was observed when the fully activated catalyst was evaluated for a second time.

Comparison of $ZnHfO_x$ catalysts derived from different synthesis routes

To investigate the effect of the synthesis route of the $ZnHfO_x$ on their catalytic performance, reference materials were synthesized by incipient wetness impregnation ($ZnHfO_{x\text{-IWI}}$) of Zn nitrate onto HfO_2, as well as a physical mixture of ZnO and HfO_2 ($ZnHfO_{x\text{-PM}}$). Both alternative synthesis routes yielded materials composed of two phases at Zn contents of 20 and 30 mol%, *i.e.* featuring monoclinic HfO_2 and hexagonal ZnO (Figure S27 and Figure S70). The co-precipitated catalysts with the same Zn contents (20 and 30 mol%) outperformed the two reference materials in CO_2 conversion and methanol selectivity, showcasing the solid solution-type structure as the most active motif for methanol formation in $ZnHfO_x$ catalysts (Figure S71). However, it is noteworthy that while the CO_2 conversions were slightly

lower (by 4 – 30 %), the methanol selectivity was high (> 65 %) for both $ZnHfO_{x\text{-PM}}$ and $ZnHfO_{x\text{-IWI}}$ at 20 and 30 mol% Zn despite the presence of hexagonal ZnO, agreeing well with the high methanol selectivity for co-precipitated catalysts with high Zn contents (> 40 mol%) that contained substantial amounts of hexagonal ZnO.

Additional remarks on CO_2 TPD of $ZnHfO_x$ catalysts

$10ZnHfO_x$ revealed a similarly broad desorption peak as HfO_2, indicating the presence of stronger basic sites. We attribute the stronger basicity of HfO_2 and $10ZnHfO_x$ to their monoclinic structure, whereas $ZnHfO_x$ catalysts with Zn contents > 10 mol% had a predominantly cubic structure (containing also some small monoclinic phase domains and segregated ZnO domains for Zn contents > 35 mol%). A similar observation has been reported for ZrO_2, *viz.* its monoclinic phase exhibits stronger basic sites than its other polymorphs (tetragonal).[19]

X-ray photoelectron spectroscopy of $ZnHfO_x$ catalysts

XPS O 1s spectra were acquired for $20ZnHfO_x$, $30ZnHfO_x$, and $90ZnHfO_x$ (after catalytic reaction). The O 1s spectra were fitted with three Gaussian peaks to account for lattice oxygen (O_I), oxygen neighboring defect sites (O_{II}), and surface hydroxyl groups (O_{III}), as previously reported in related, $ZnZrO_x$-based studies (Figure S72).[20–22] The O_{II} peak, which has been correlated to the concentration of oxygen vacancies in a material, is present in all of the three catalysts and notably very pronounced in the case of $90ZnHfO_x$. However, the assignment of the peak at a binding energy 1 – 2 eV higher than the main O 1s peak of lattice oxygen (O_I) to oxygen vacancies has been under considerable debate.[23,24] In particular in the case of ZnO, which constitutes the main phase of $90ZnHfO_x$, a DFT-based study has attributed the O_{II} peak to surface adsorbed oxygen species (e.g. from chemisorbed water) instead of oxygen close to defect sites.[6] In this context, $ZnZrO_x$-based reports have also assigned the O_{II} and O_{III} peaks to oxygen of surface adsorbed species rather than oxygen vacancies.[25,26] It is also noteworthy that a deconvolution of the O 1s spectra of the reacted catalysts into only two Gaussian peaks (O_{I^*}, O_{II^*}) yields an equally good fit compared to a deconvolution into three Gaussian peaks (Figure S66). Consequently, in particular considering the debate on the assignment and identification of oxygen vacancies by XPS, we refrain from over-interpreting the XPS data.

Extending the active site discussion to $ZnHfO_x$ catalysts at very high Zn contents

We performed additional *in-situ* DRIFTS on $90ZnHfO_x$ and compared the results to the data acquired for $20ZnHfO_x$ and ZnO under identical conditions. We observe that the IR signatures of $90ZnHfO_x$ are very similar to that of $20ZnHfO_x$ with regard to wavenumber position and evolution of surface adsorbates/reaction intermediates, demonstrating that on both materials similar active sites for methanol formation are at play (Figure S56). Specifically, at 20 bar and 320 °C in 20 vol.% CO_2 in N_2, $90ZnHfO_x$ exhibits bands for bidentate carbonate (b-CO_3*) and bidentate bicarbonate (b-CO_3H*) at a similar intensity ratio and at almost identical positions compared to $20ZnHfO_x$, demonstrating that the activation of CO_2 on high Zn content $ZnHfO_x$ catalysts that mainly contain HfO_x clusters on ZnO is very similar to that on solid solution-type $ZnHfO_x$ catalysts (20 % Zn content). On the contrary, pure ZnO features very different IR spectra in a CO_2 containing environment, with bands at 1511 and 1324 cm^{-1} assigned to polydentate carbonates (p-CO_3*), at 1595 cm^{-1} assigned to bidentate carbonates (b-CO_3*), and at 1370 cm^{-1} due to linearly adsorbed CO_2 species (CO_2*).[27,28]

Furthermore, under reaction conditions (320 °C, 20 bar, H_2:CO_2:N_2 = 3:1:1), the bands of the reaction intermediates formate (HCOO*) and methoxy (H_3CO*) appear at respectively 2892 and 2832 cm^{-1} for $20ZnHfO_x$ and at 2894 and 2835 cm^{-1} for $90ZnHfO_x$, again demonstrating almost identical band positions, which allows us to infer that the reaction intermediates are stabilized at very similar active motifs. In contrast, on ZnO, the bands for formate and methoxy shift significantly to 2873 and 2825 cm^{-1}, respectively.

When switching from a CO_2 hydrogenation mixture to H_2 at 20 bar and 320 °C on $90ZnHfO_x$ the intensity of the bands due to adsorbed formate species decrease, while the bands due to adsorbed methoxy species increase. Such dynamic behaviour is also observed on $20ZnHfO_x$ confirmed that also on $90ZnHfO_x$, methanol is produced via the formate-methoxy pathway. On ZnO (after switching to a H_2 atmosphere), also the intensity of the band due to adsorbed formate species decreases, however, the increase in methoxy bands is less pronounced compared to Hf containing catalysts, indicating that the presence of Hf in the active site motif stabilizes methoxy species. While an overstabilization of methoxy species can impede the catalytic turnover, the stabilization of methoxy species in general has been reported to be beneficial for methanol selectivity.[29] It is noteworthy that the intensity of the bands due to reactive species is lower on $90ZnHfO_x$ compared to $20ZnHfO_x$, which can be attributed to an overall smaller surface area and/or a lower density of active sites and correlates well with the lower methanol yield of $90ZnHfO_x$ compared to $20ZnHfO_x$.

We further determined the apparent activation energies for methanol and CO formation on $ZnHfO_x$ catalysts with low ($20ZnHfO_x$, $30ZnHfO_x$) and high ($90ZnHfO_x$, $98ZnHfO_x$, and $99ZnHfO_x$) Zn contents as well as ZnO (Figure S57, Figure S58). The apparent activation energies for methanol formation are in a range of 77 – 83 kJ/mol for the $ZnHfO_x$ catalysts with Zn contents in the range 20 – 98 mol% Zn, i.e. the activation energy is independent of the Hf content, providing evidence that for both low and high Zn content catalysts methanol synthesis proceeds over similar Zn-V_O-Hf motifs. Even $99ZnHfO_x$ exhibits an apparent activation energy for methanol formation of 73 kJ/mol. In comparison, pure ZnO has an apparent activation energy for methanol formation of approximately 40 kJ/mol, i.e. a very different value compared to the $ZnHfO_x$-based catalysts. A slightly larger variance was observed in the apparent activation energies for CO formation, ranging between 95 – 115 kJ/mol for $ZnHfO_x$ catalysts for Zn contents in the range 20 – 99 mol%. Nevertheless, all Hf containing catalysts exhibited significantly higher activation energies for CO formation compared to pure ZnO (80 kJ/mol), which explains their higher methanol selectivity.

Summarizing, *in-situ* DRIFTS demonstrates that the reaction intermediates adsorb on chemically similar sites on solution-type $ZnHfO_x$ catalysts (Zn content < 40 mol%) and HfO_x cluster on ZnO-type $ZnHfO_x$ catalysts (Zn content $\gg$ 40 mol%). In addition, Arrhenius analysis reveals almost identical apparent activation energies for methanol formation for $ZnHfO_x$ catalysts containing 20 – 99 mol% Zn. Combining these spectroscopic and kinetic results, we infer that Zn-V_O-Hf sites, as determined as the active sites in solid solution-type $ZnHfO_x$ catalysts, are also the dominating active motifs for methanol formation at very high Zn contents ($\gg$ 40 mol%) in which HfO_x clusters decorate the surface of ZnO.

To explore Zn-rich catalyst configurations, we extend the DFT analysis by doping Hf into the ZnO(1010) surface lattice (replacing one lattice Zn with Hf, Figure S66), hereafter referred to as the HfZnO surface. As shown in Table S3, the H_2 adsorption energy on HfZnO is very similar to that on ZnO, i.e. the difference is only 0.07 eV. However, Hf incorporation enhances the stabilization of heterolytic H_2 dissociation. The calculated energy barriers for this process are low, i.e. 0.22 eV for HfZnO and 0.16 eV for ZnO. These results suggest that Zn primarily lowers the activation barrier for the hydrogen splitting, whereas Hf stabilizes the dissociation products as indicated by a lower adsorption energy of dissociated hydrogen of −0.83 eV compared to −0.37 eV on pristine ZnO (Figure S55). In contrast, H_2 dissociation on pure HfO_2 exhibits a higher energy barrier of 0.66 eV and poor stabilization of the adsorbed hydrogen species with an adsorption energy of 0.19 eV (Figure S55). Although the energy barrier for hydrogen splitting is reduced on the $ZnHfO_2$ slab, the resulting hydrogen species are overly stabilized with an

adsorption energy of −5.49 eV. This over-stabilization hinders the subsequent reaction steps by increasing their associated energy barriers, suggesting that oxygen vacancies are required to enhance the overall reactivity of this surface.

Concerning CO_2 adsorption and subsequent HCOO* formation, both intermediates are energetically more stable on the HfZnO surface, with adsorption energies of −1.01 and −2.84 eV, respectively, when compared to HfO_2, HfO_x (Figure S55), and ZnO (Table S3). This suggests that the presence of Zn in the vicinity of Hf, either on HfO_2 or ZnO-based surfaces, modulates the adsorption strength of CO_2. In addition, the co-adsorption strength of CO_2 in the presence of pre-dissociated hydrogen on HfZnO is still weaker compared to low-Zn-content HfO_2 surfaces without oxygen vacancies ($ZnHfO_2$), and this is also found to allow the reaction on HfZnO to proceed with a low activation barrier of 0.06 eV for the formate formation step. A possible reason for the weaker co-adsorption strength of CO_2 and lower barrier for formate formation on HfZnO (compared to $ZnHfO_2$) might be due to the fact that the incorporation of Hf into the ZnO lattice introduces undercoordinated Hf centers. This Hf centers appears to partially mimic Zn–V_O–Hf motifs, thereby compensating for the lack of “conventional” oxygen vacancies.

However, the absence of conventional oxygen vacancies on HfZnO surfaces, given by their positive formation energies (Figure S73), may increase the energy barriers of subsequent reaction steps along the formate pathway, particularly H_2COO* bond cleavage to form CH_2O*, which typically occurs at oxygen vacancy sites. In addition, in the absence of oxygen vacancies, the stabilization of the intermediate CH_3O* is also compromised, as such species are preferentially stabilized at V_O sites.

Supplementary Figures

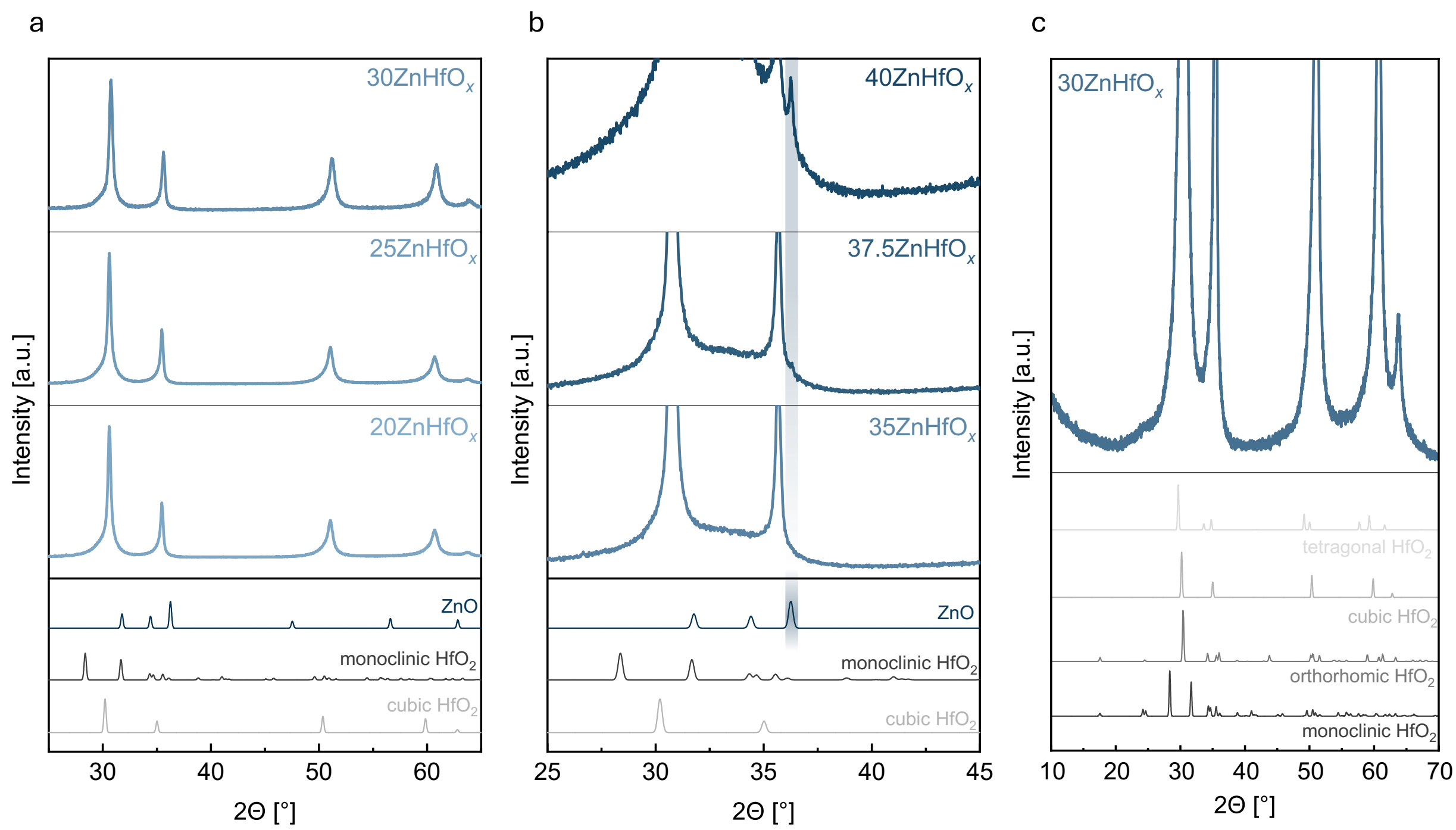

Figure S1: XRD analysis of as-prepared ZnHfO$_x$ catalysts with Zn contents ranging from 20 – 40 mol%. (a) XRD patterns of 20ZnHfO$_x$, 25ZnHfO$_x$, and 30ZnHfO$_x$ showing an asymmetric peak shape that is tailing towards lower diffraction angles for all three catalysts. (b) Enlarged XRD patterns of 35ZnHfO$_x$, 37.5ZnHfO$_x$, and 40ZnHfO$_x$, demonstrating an increase in the amorphous halo with increasing Zn content. (c) Enlarged XRD pattern of 30ZnHfO$_x$, showing a slight amorphous halo, as well as phase impurities indicating the presence of monoclinic phase domains.

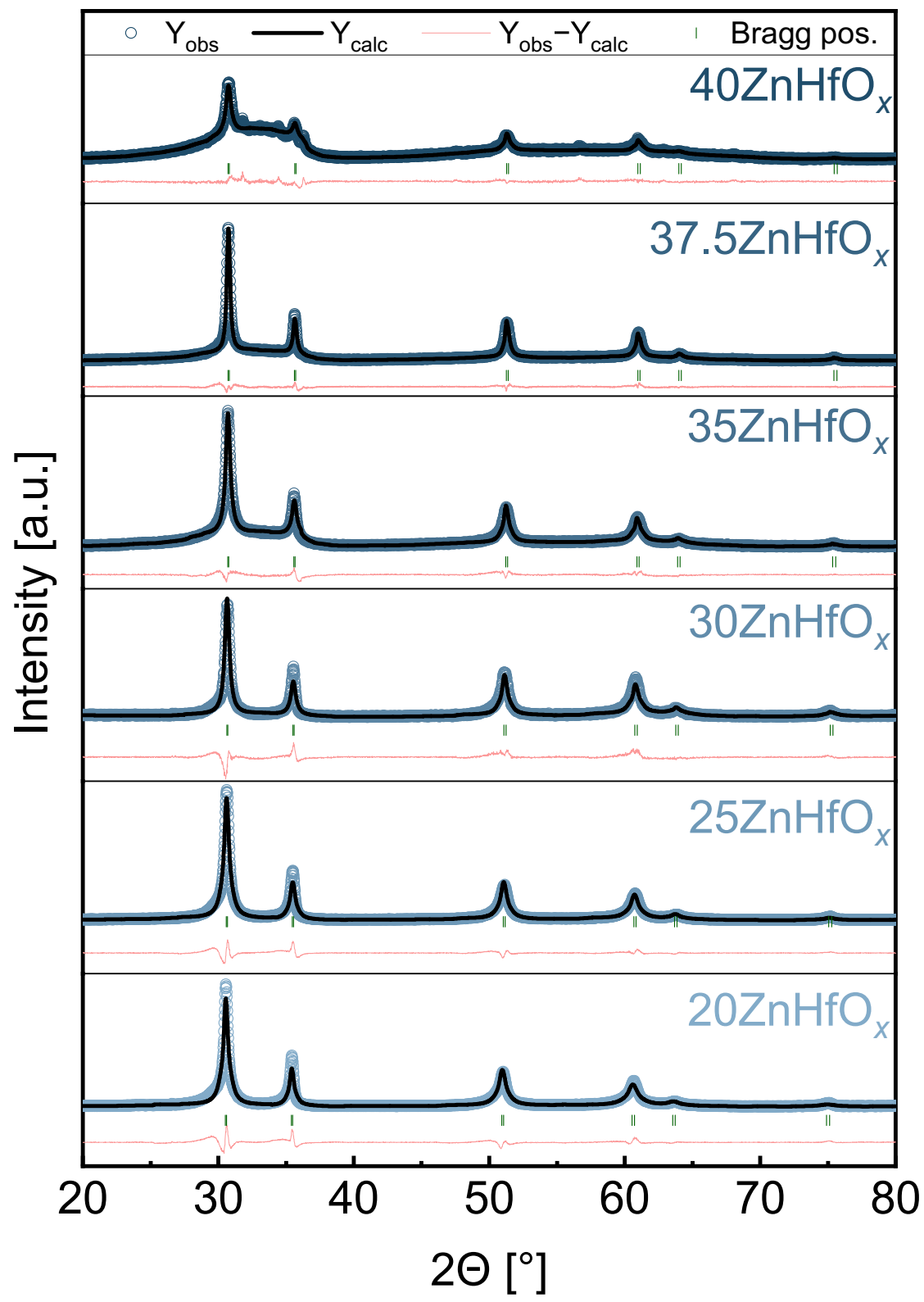

Figure S2: Rietveld refinements of as-prepared $ZnHfO_x$ with Zn contents ranging from 20 – 40 mol%.

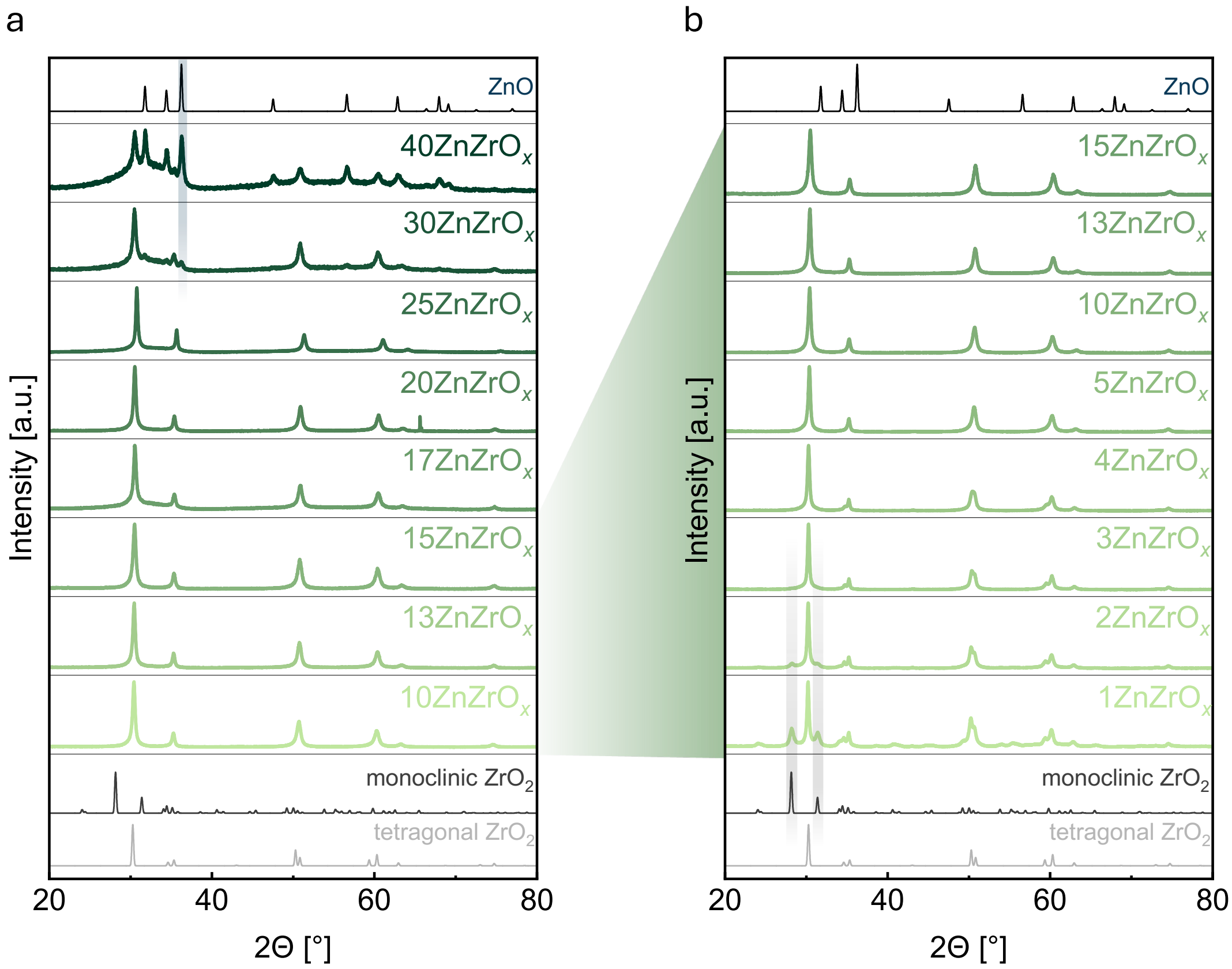

Figure S3: XRD patterns of the as-prepared $ZnZrO_x$ catalysts. (a) XRD patterns of $ZnZrO_x$ catalysts with Zn contents ranging from 10 – 40 mol%. Segregation of bulk, hexagonal ZnO is observed for Zn contents ≥ 30 mol%. (b) XRD patterns of $ZnZrO_x$ catalysts with Zn contents ranging from 10 – 40 mol%. The tetragonal phase is stabilized for Zn content > 4 – 5 mol%.

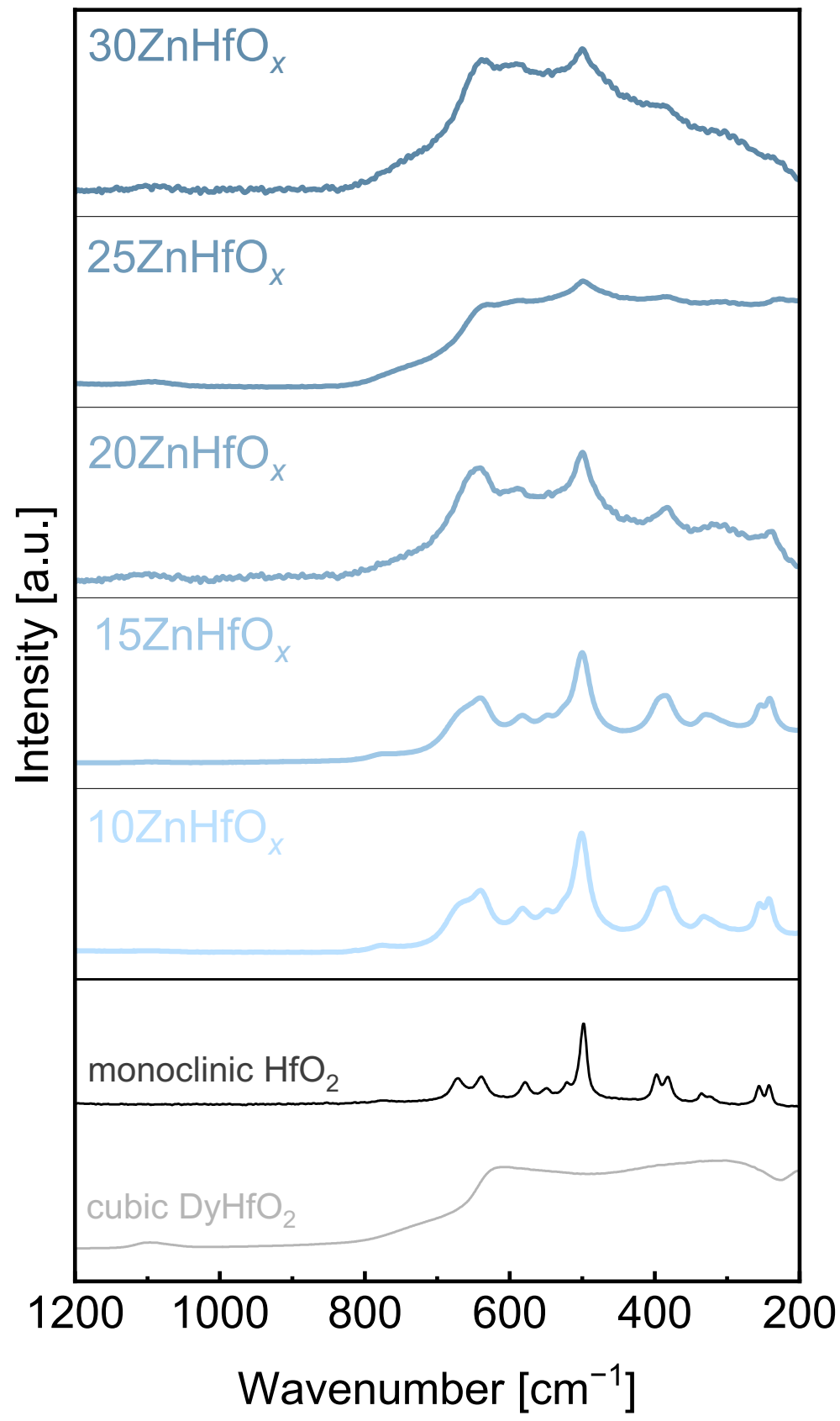

Figure S4: Raman spectra of as-prepared $ZnHfO_x$ catalysts with Zn contents ranging from 10 – 30 mol%. Dy-doped HfO_2 (12 mol% Dy) is used as a reference for cubic HfO_2.

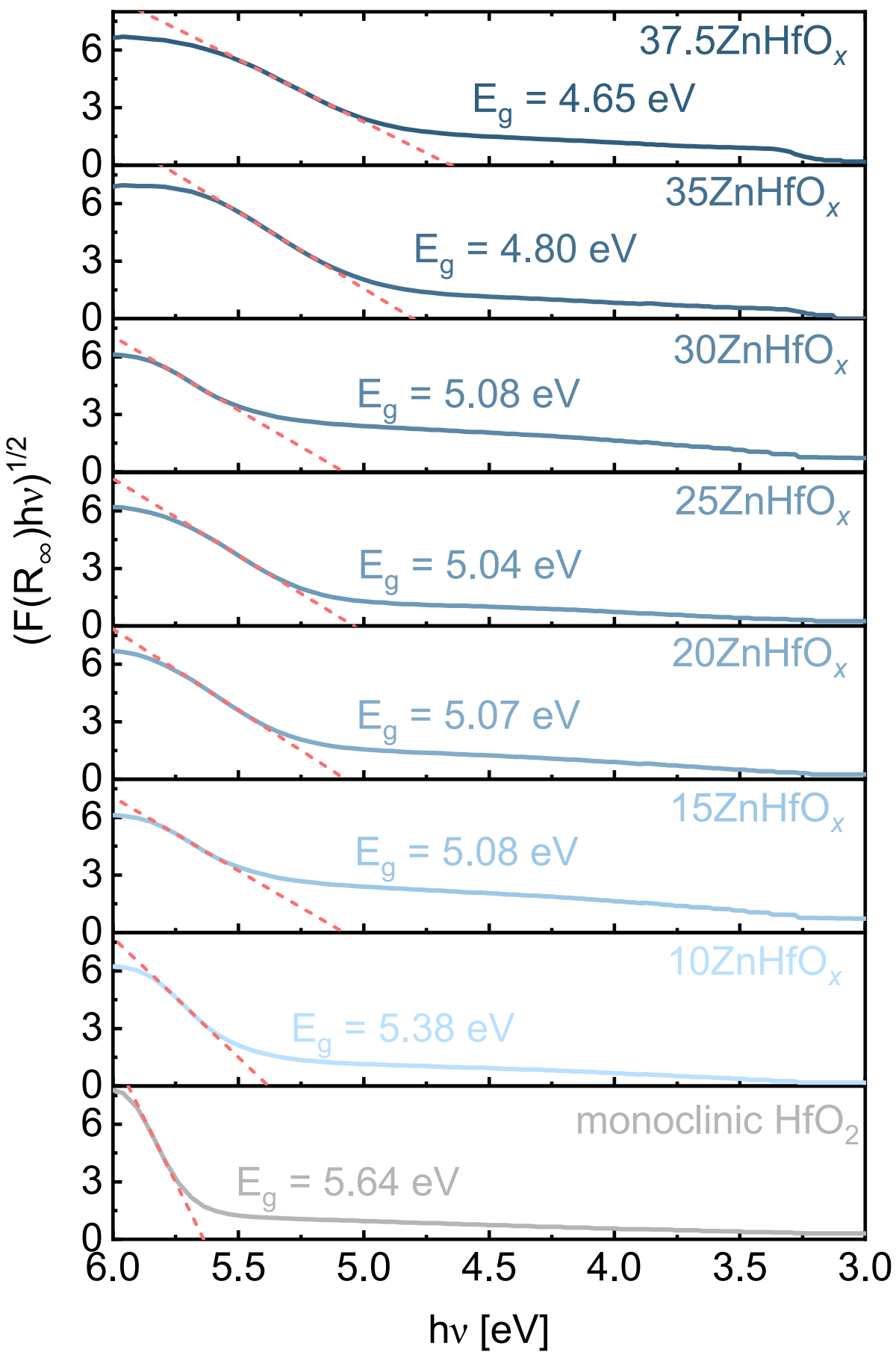

Figure S5: Tauc plots of as-prepared ZnHfO$_x$ UV-vis spectra with Zn contents ranging from 10 – 37.5 mol%. Tauc plots of HfO$_2$ and ZnHfO$_x$ with Zn contents ranging from 10 – 37.5 mol%. The intersection of the linear fit of the absorbance step (red dashed line) with the ordinate yields the energy of the bandgap in eV.

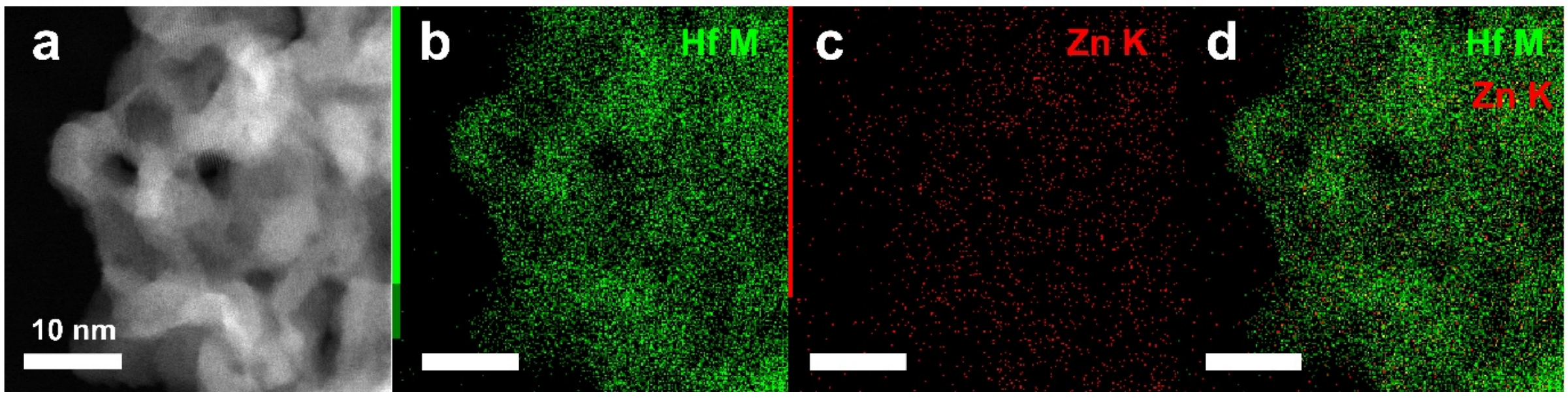

Figure S6: Electron microscopy of 20ZnHfO$_x$. (a) HAADF-STEM image of the area of 20ZnHfO$_x$ studied by EDX. Elemental maps of (b) Hf M, (c) Zn K EDX signals, and (d) their overlap.

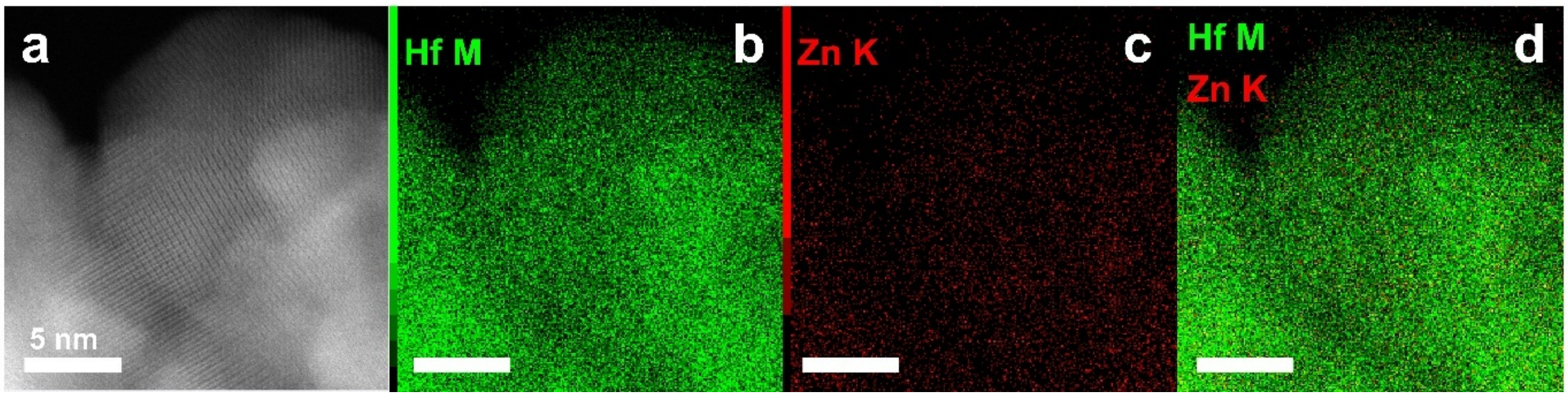

Figure S7: Electron microscopy of 30ZnHfO$_x$. (a) HAADF-STEM image of the area of 30ZnHfO$_x$ studied by EDX. Elemental maps of (b) Hf M, (c) Zn K EDX signals, and (d) their overlap.

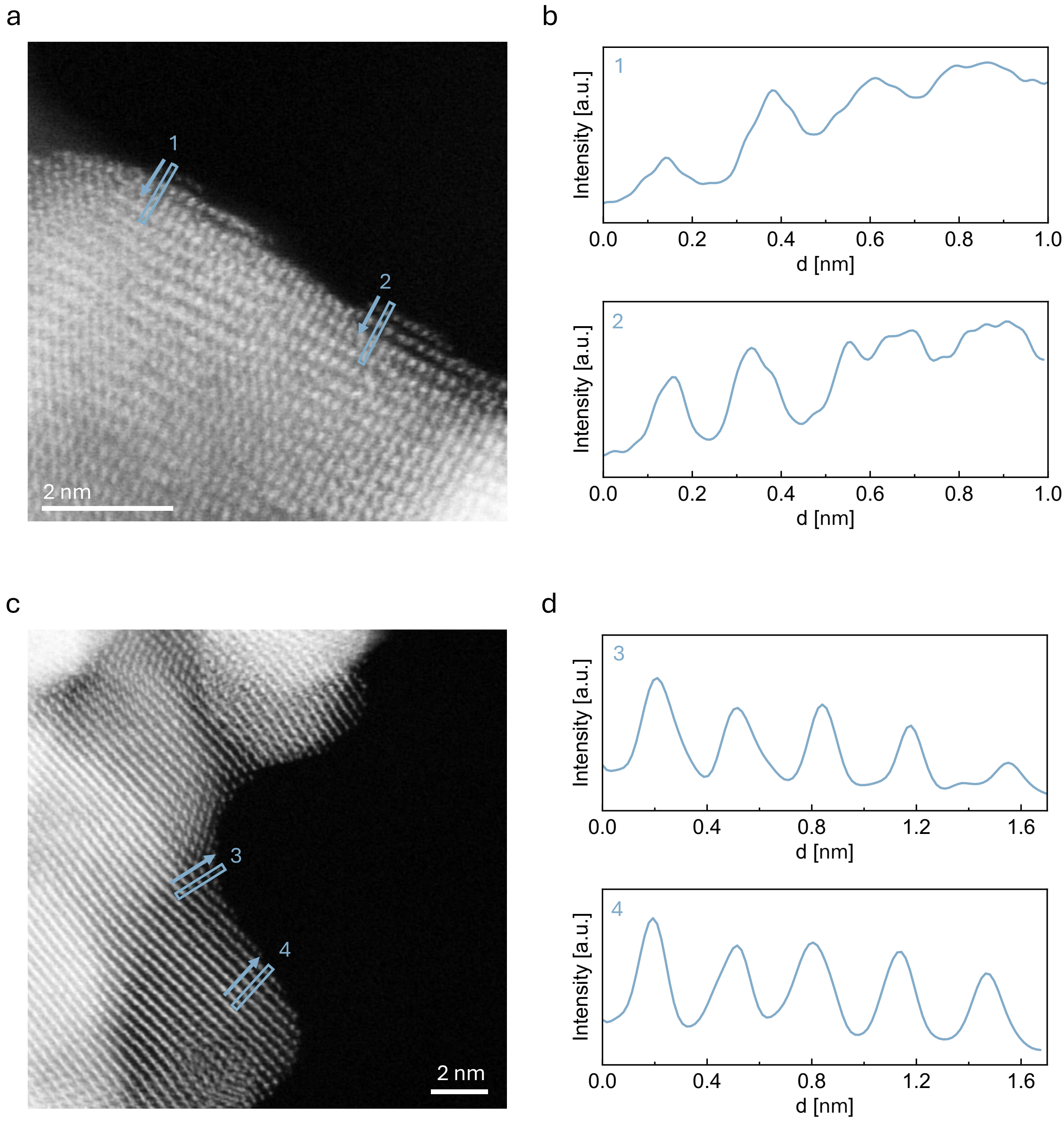

Figure S8: High resolution electron microscopy of 20ZnHfO$_x$. (a) And (c) high-angle annular dark-field STEM images of 20ZnHfO$_x$. (b) And (d) line profiles of the HAADF intensity across the surface regions in (a) and (d) corresponding to the respective numbers.

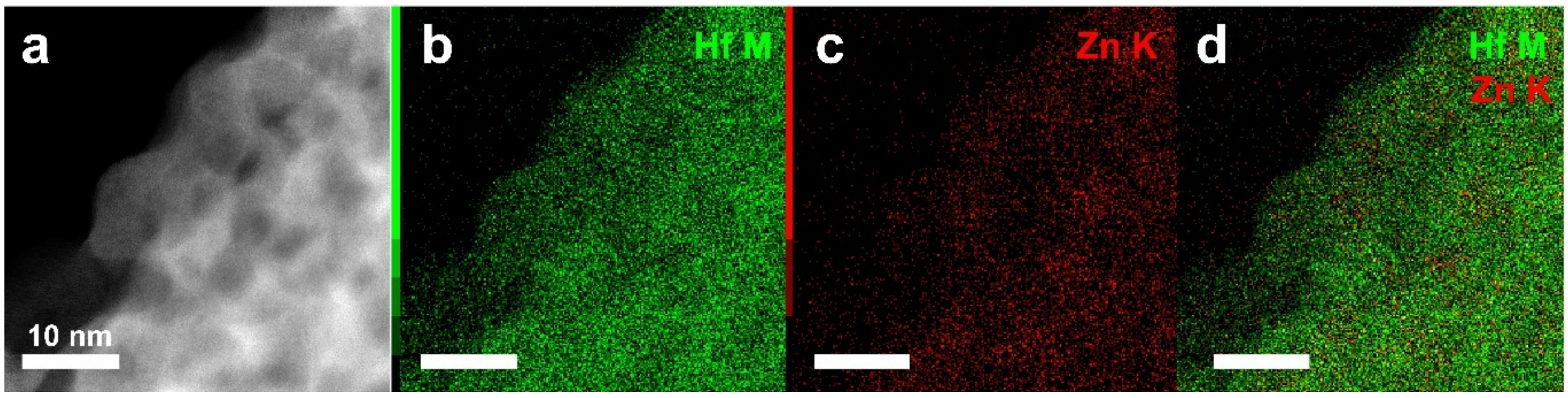

Figure S9: Electron microscopy of 37.5ZnHfO$_x$. (a) HAADF-STEM image of the area of 37.5ZnHfO$_x$ studied by EDX. Elemental maps of (b**)** Hf M, (c) Zn K EDX signals, and (d**)** their overlap.

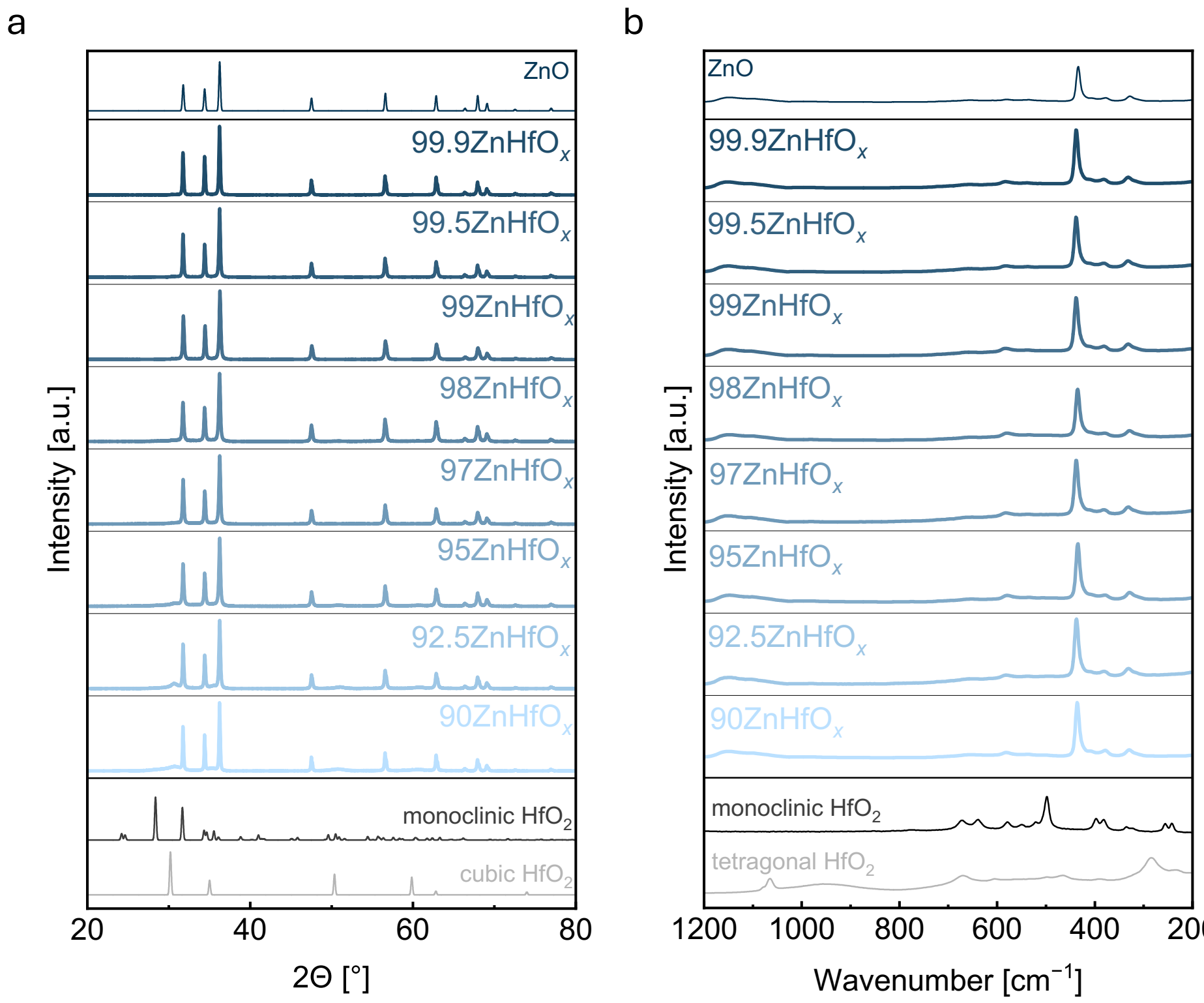

Figure S10: Structural characterization of $ZnHfO_x$ catalysts at very high Zn contents (90 – 99.9 mol%). (a) XRD patterns and (b) Raman spectra of $ZnHfO_x$ catalysts with a Zn content ranging from 90 – 99.9 mol%.

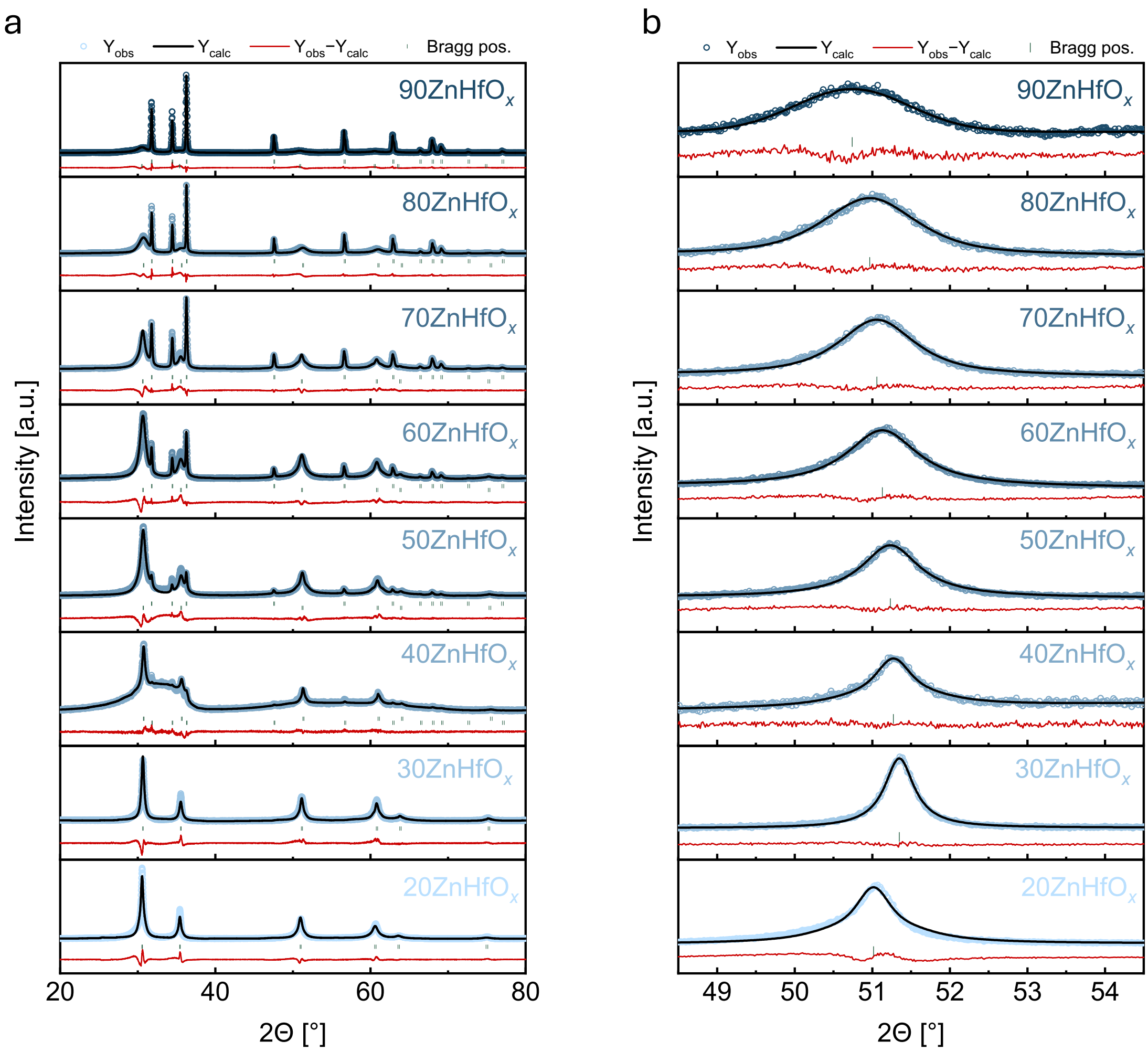

Figure S11: Diffraction pattern refinement of as-prepared $ZnHfO_x$. (a) Multi-phase Rietveld refinements of $ZnHfO_x$ with Zn contents ranging from 20 – 90 mol% Zn. Cubic HfO_2 was used to fit Zn doped HfO_2, and hexagonal ZnO was used to fit the segregated, crystalline ZnO. (b) Peak fits of the (220) reflection of cubic HfO_2 in the XRD patterns of $ZnHfO_x$ with Zn contents ranging 20 – 90 mol% to obtain the average crystallite size from the Scherrer equation.

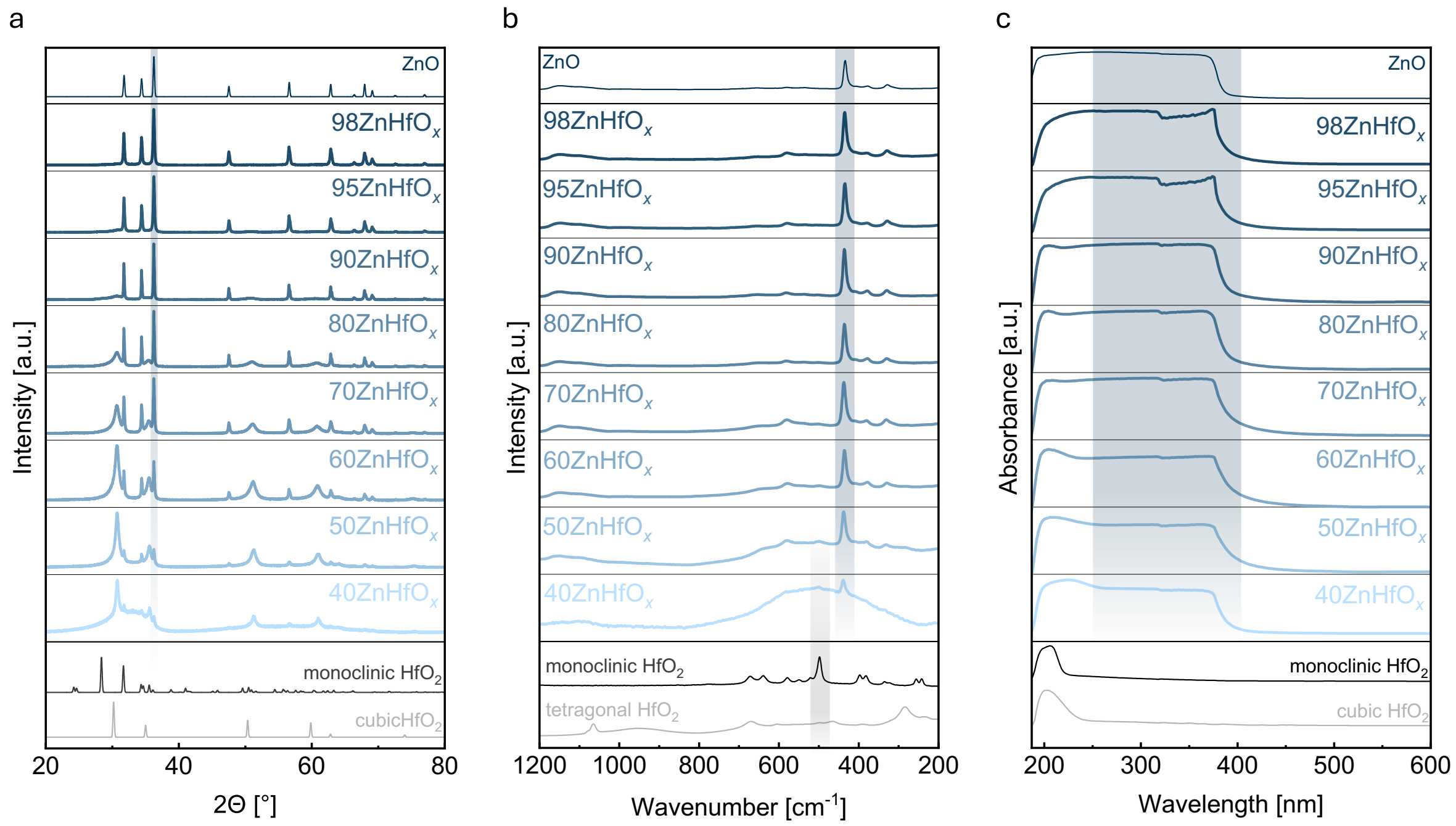

Figure S12: Structural characterization of as-prepared $ZnHfO_x$ catalysts with Zn contents ≥ 40 mol%. (a) XRD patterns, (b) Raman spectra, and (c) UV-vis spectra of $ZnHfO_x$ catalysts with Zn contents ≥ 40 mol%.

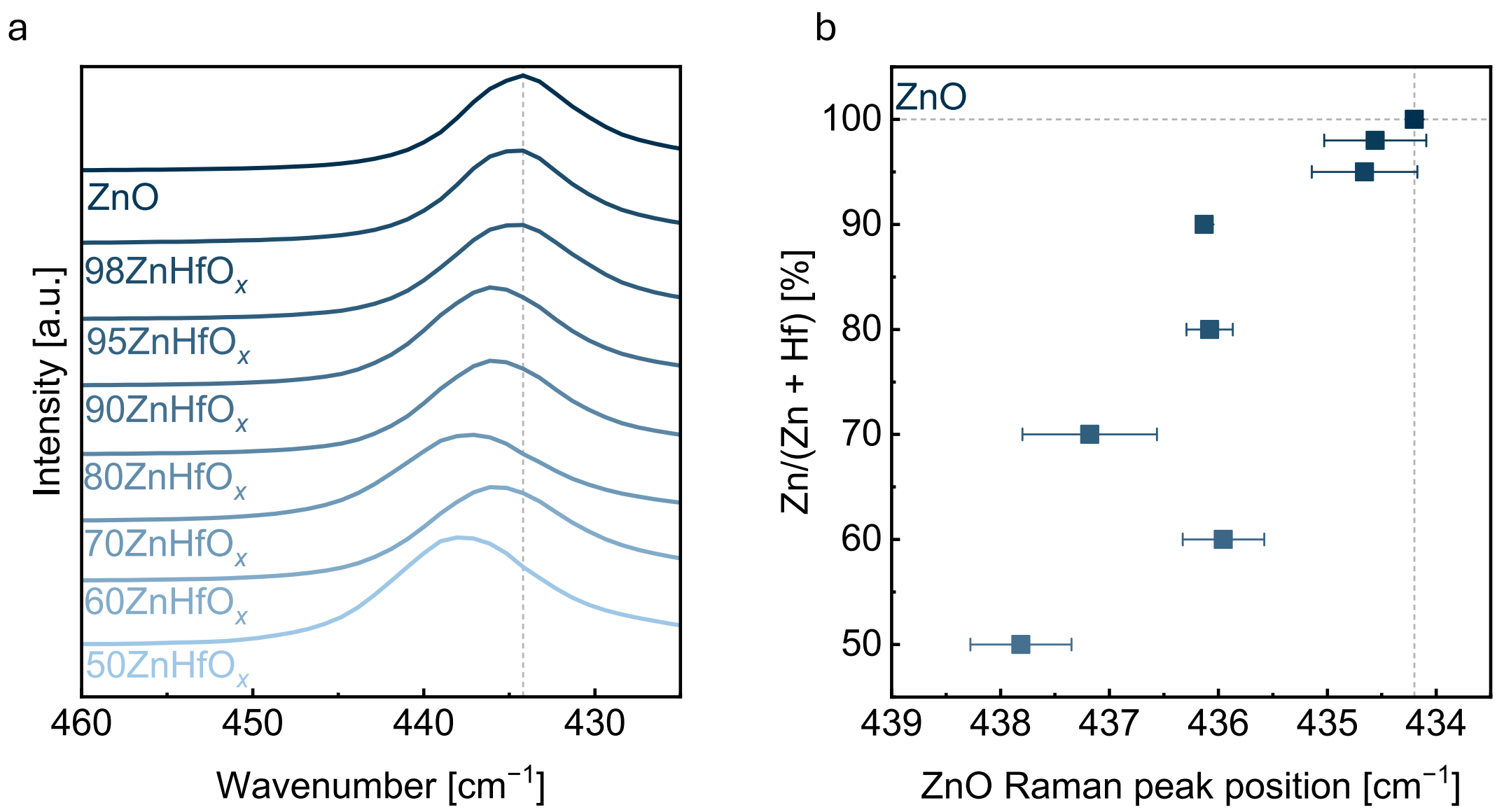

Figure S13: Raman spectroscopy of $ZnHfO_x$ for Zn contents ranging from 50 – 98 mol%. (a) Enlarged Raman spectra of $ZnHfO_x$ for Zn contents ranging from 50 – 98 mol% revealing a peak shift with respect to pure ZnO. Every Raman spectrum is an average of 100 individual scans. (b) Average and standard deviation of the peak maximum due to the ZnO vibration of 100 recorded scans.

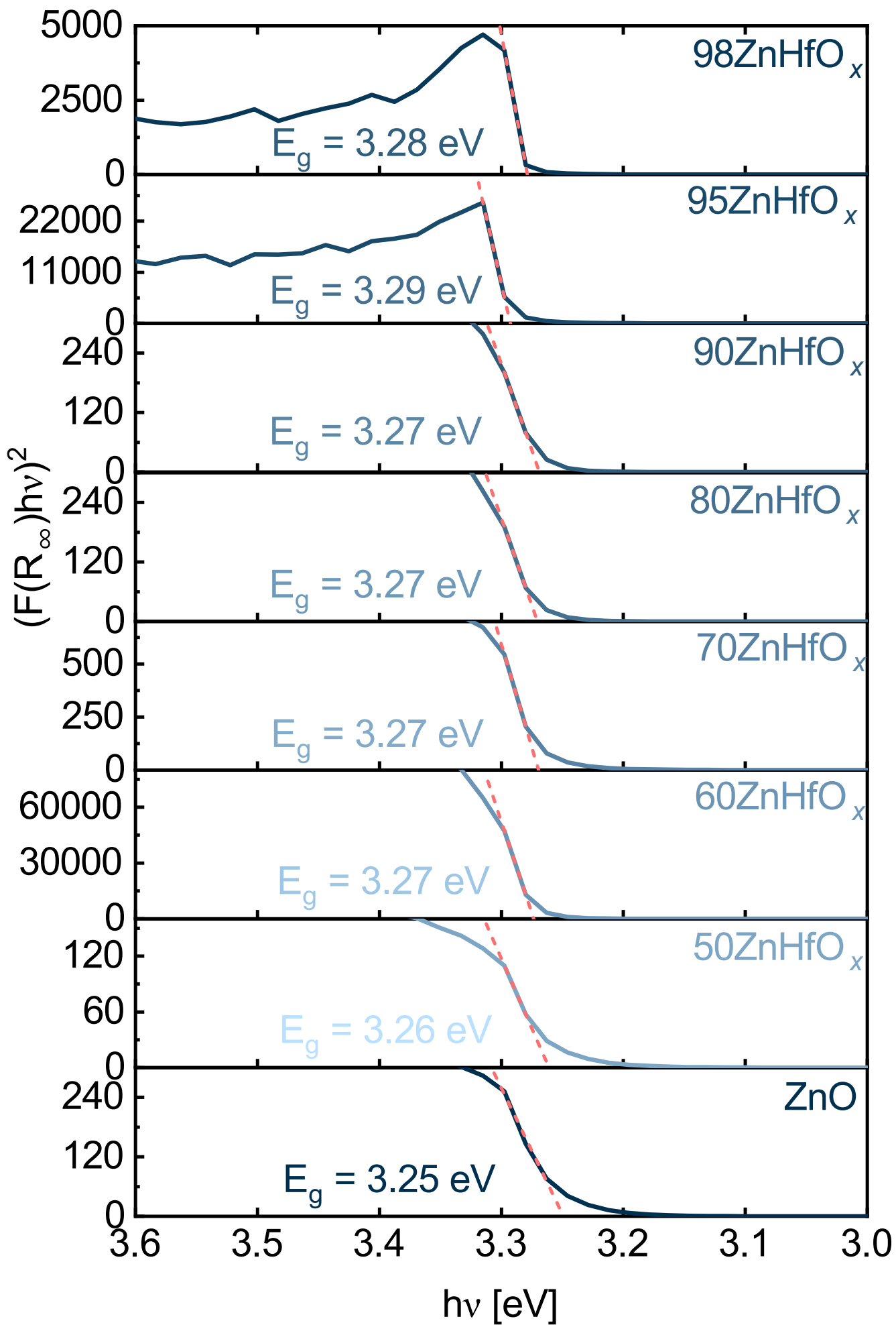

Figure S14: Tauc plots of as-prepared $ZnHfO_x$ UV-vis spectra with Zn contents ranging from 50 – 98 mol%. Tauc plots of ZnO as well as $ZnHfO_x$ spectra with Zn contents ranging from 50 – 98 mol%. The intersection of the linear fit of the absorbance step (red dashed line) with the ordinate yields the energy of the bandgap in eV.

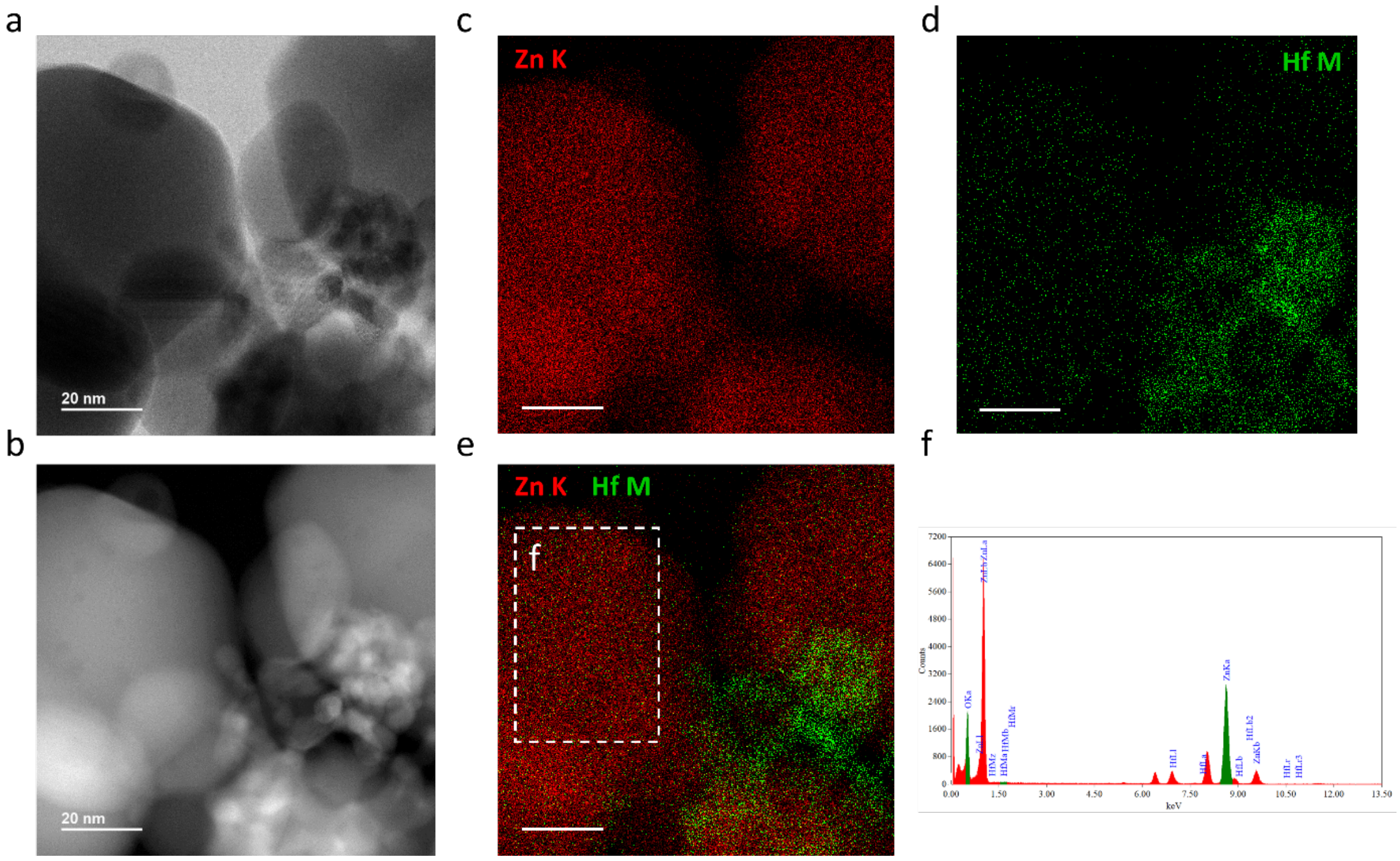

Figure S15: Electron microscopy of 98ZnHfO$_x$. (a) Annular bright field-STEM (ABF) and (b) HAADF-STEM image of the area of 98ZnHfO$_x$ studied by EDX. Elemental maps of (c) Zn K, (d) Hf M EDX signals, and (e) their overlap. (f) EDX spectrum integrated from the area marked in (e).)

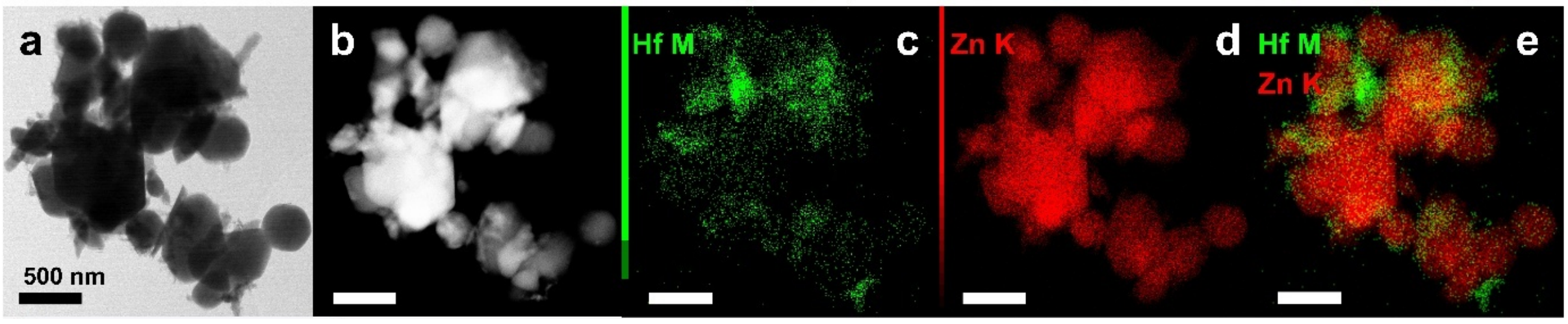

Figure S16: Electron microscopy of 90ZnHfO$_x$ showing different morphologies of ZnO and Hf-rich domains. (a) Annular bright field-STEM (ABF) and (b) HAADF-STEM image of the area of 90ZnHfO$_x$ studied by EDX. (Elemental maps of (a) Hf M, (b) Zn K EDX signals, and (d) their overlap.)

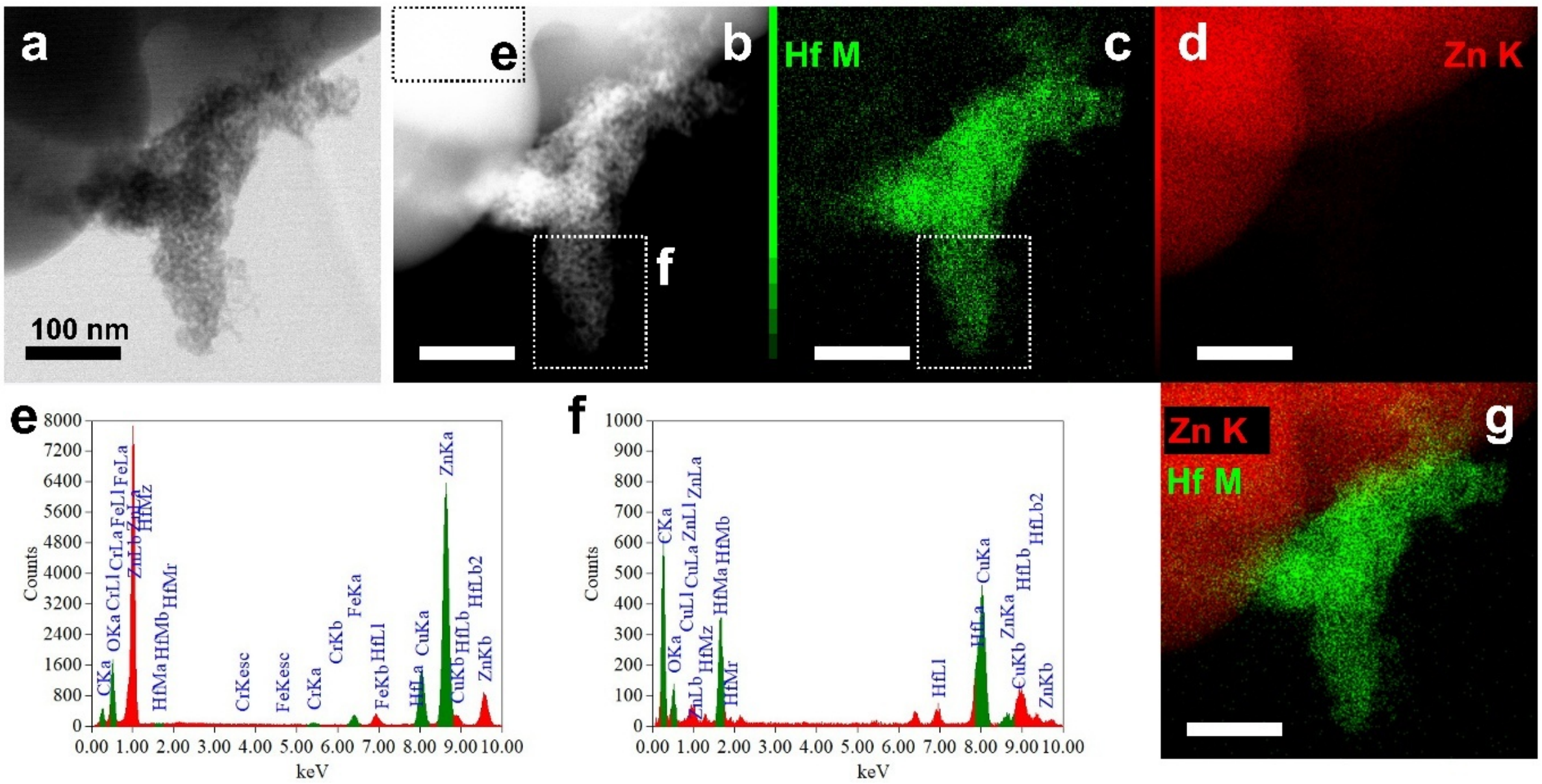

Figure S17: Electron microscopy of 90ZnHfO$_x$. (a) Annular bright field-STEM (ABF) and (b) HAADF-STEM image of the area of 90ZnHfO$_x$ studied by EDX. (Elemental maps of (c) Hf M, (d) Zn K EDX signals, and (g) their overlap. (e) and (f) EDX spectra integrated from the areas shown in (b).)

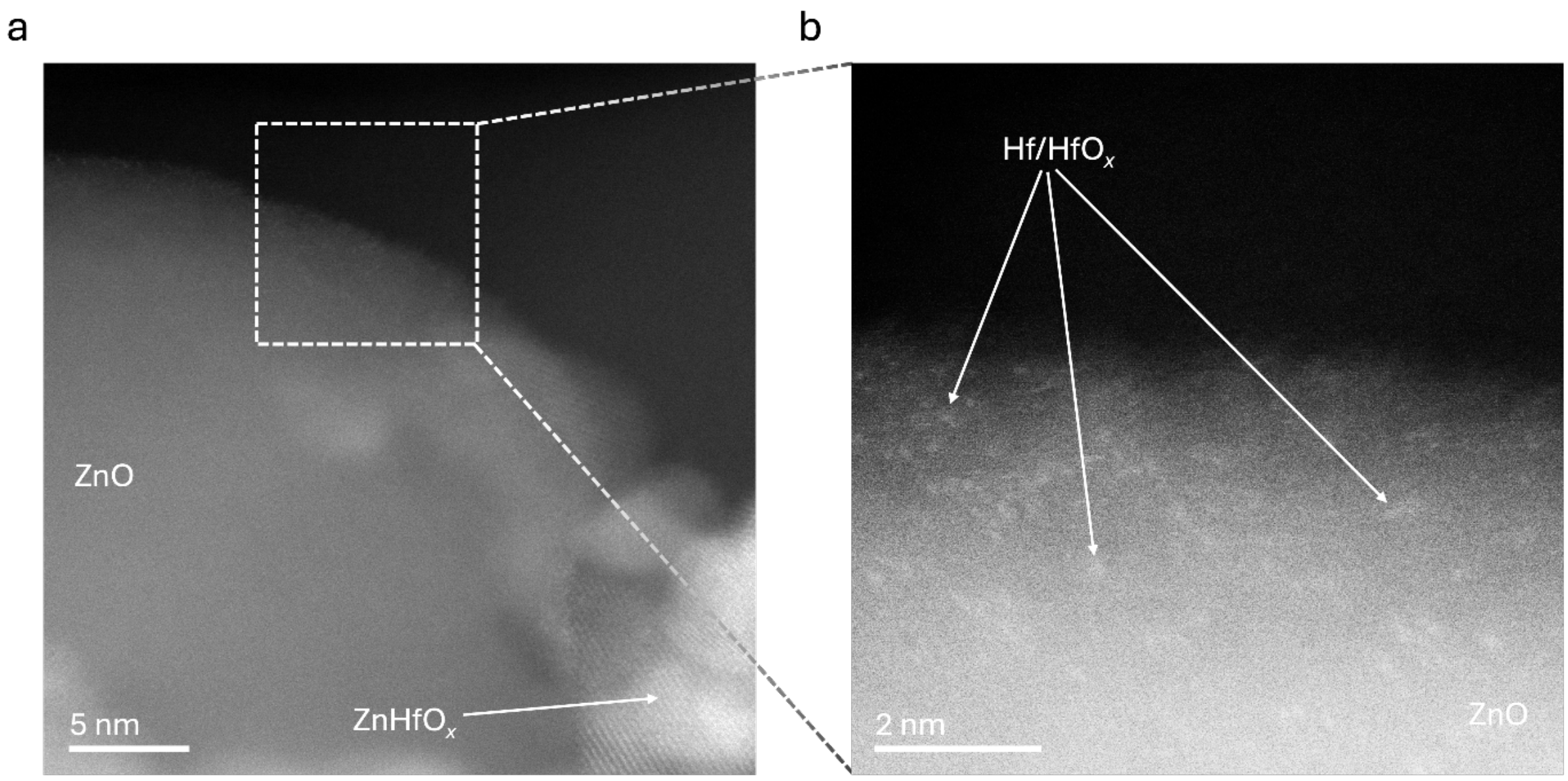

Figure S18: HAADF-STEM images of 90ZnHfO$_x$. (a) HAADF-STEM image of 90ZnHfO$_x$ showing different domains of segregated bulk ZnO and nanocrystalline ZnHfO$_x$ particles. (b) Higher resolution HAADF-STEM image of the 90ZnHfO$_x$ surface reveals Hf/HfO$_x$ single atoms/clusters.

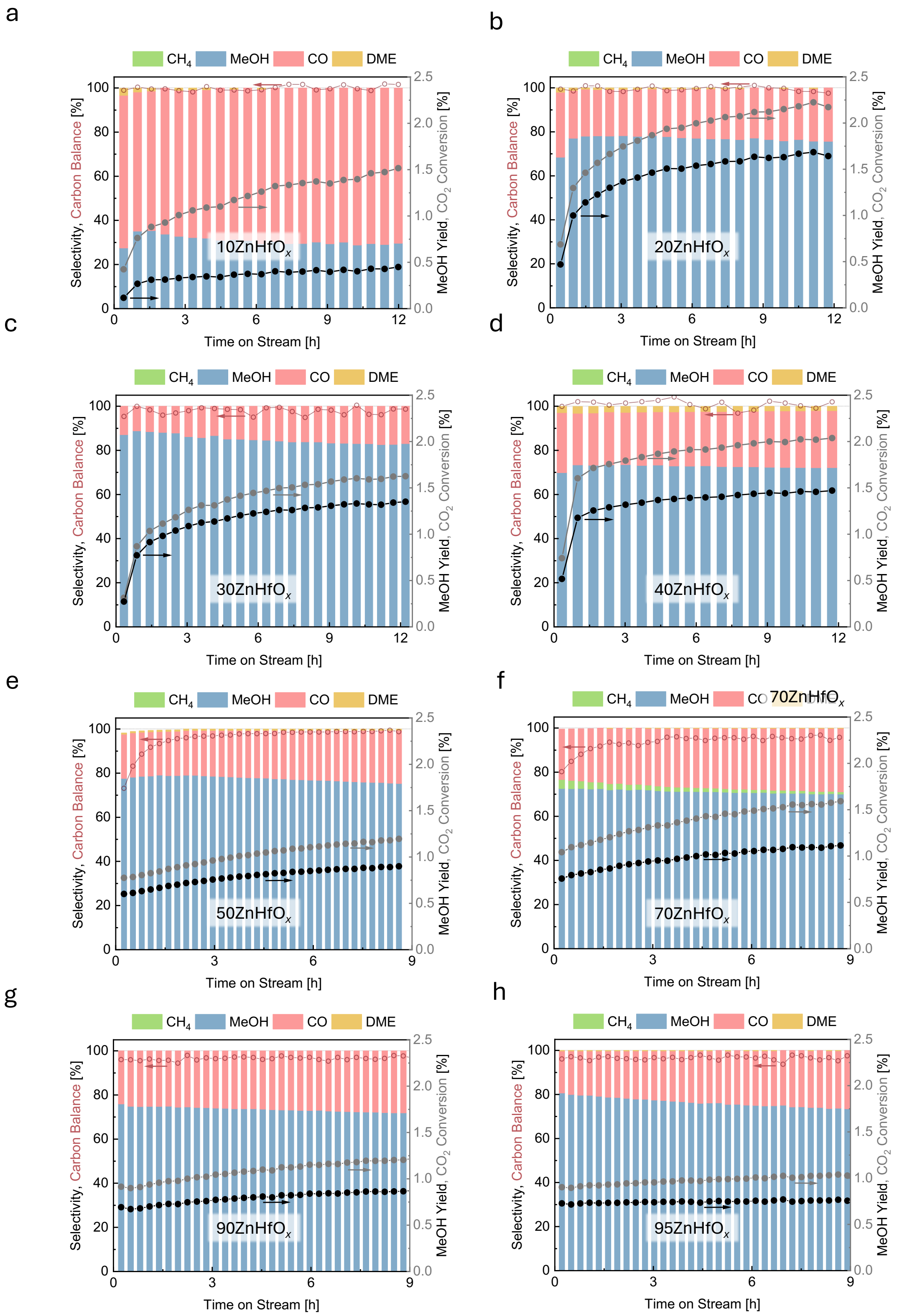

Figure S19: Catalytic performance of ZnHfO$_x$ catalysts with Zn contents ranging from 10 – 95 mol%. The catalytic performance of (a) 10ZnHfO$_x$, (b) 20ZnHfO$_x$, (c) 30ZnHfO$_x$, (d) 40ZnHfO$_x$, (e) 50ZnHfO$_x$, (f) 70ZnHfO$_x$, (g) 90ZnHfO$_x$, and (h) 95ZnHfO$_x$ at 20 bar, 320 °C, H_2:CO_2:N_2 = 3:1:1, and 24 L(g_{cat}h)$^{-1}$.

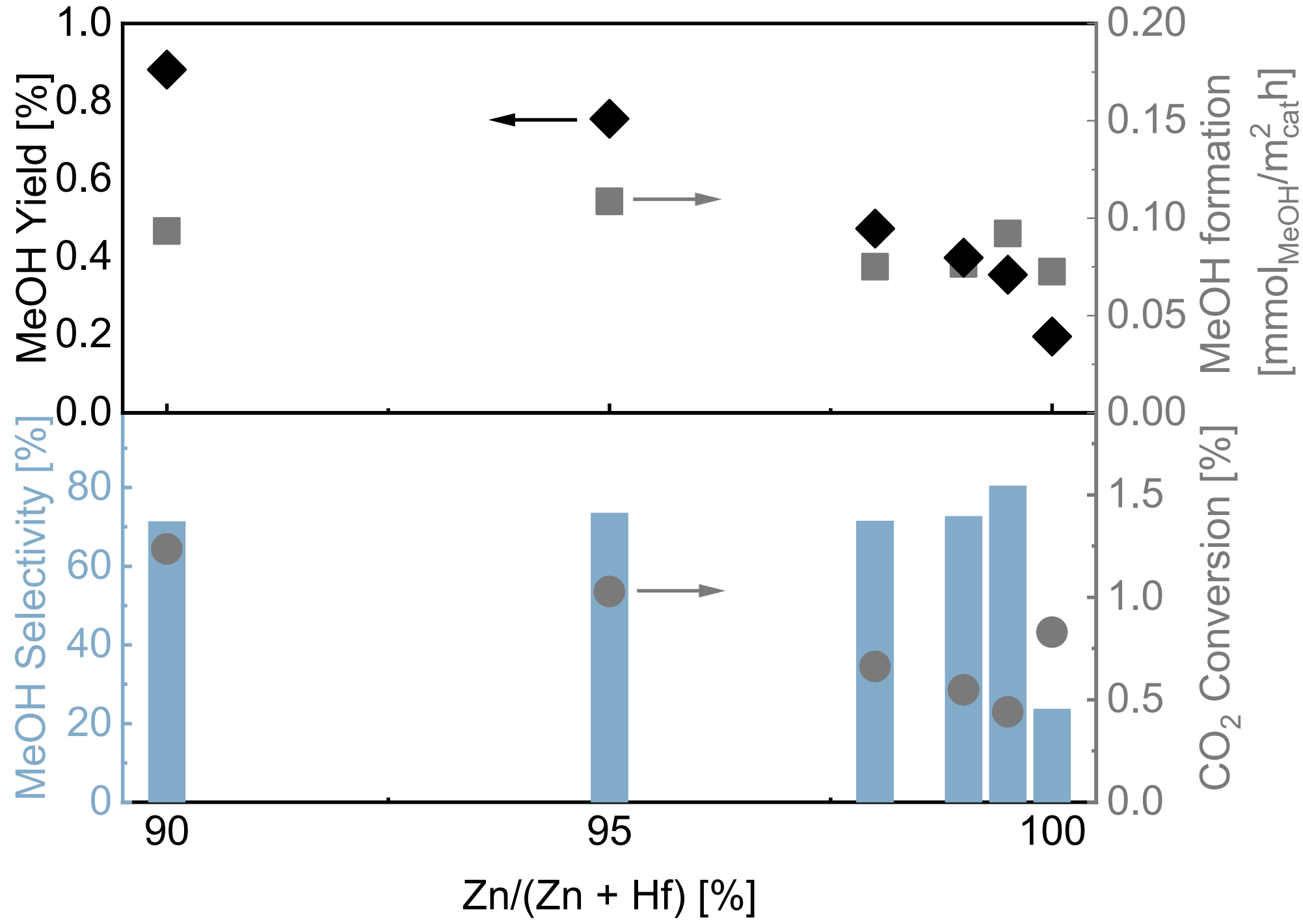

Figure S20: Catalytic performance of ZnHfO$_x$ catalysts at very high Zn contents (90 – 99.9 mol%). Methanol yield, methanol formation rate (surface area-normalied), selectivity, and CO_2 conversion of ZnHfO$_x$ catalysts with Zn contents ranging from 90 – 99.5 mol%. Catalytic tests were carried out at 320 °C, 20 bar, and GHSV = 24 L/(g_{cat}h).

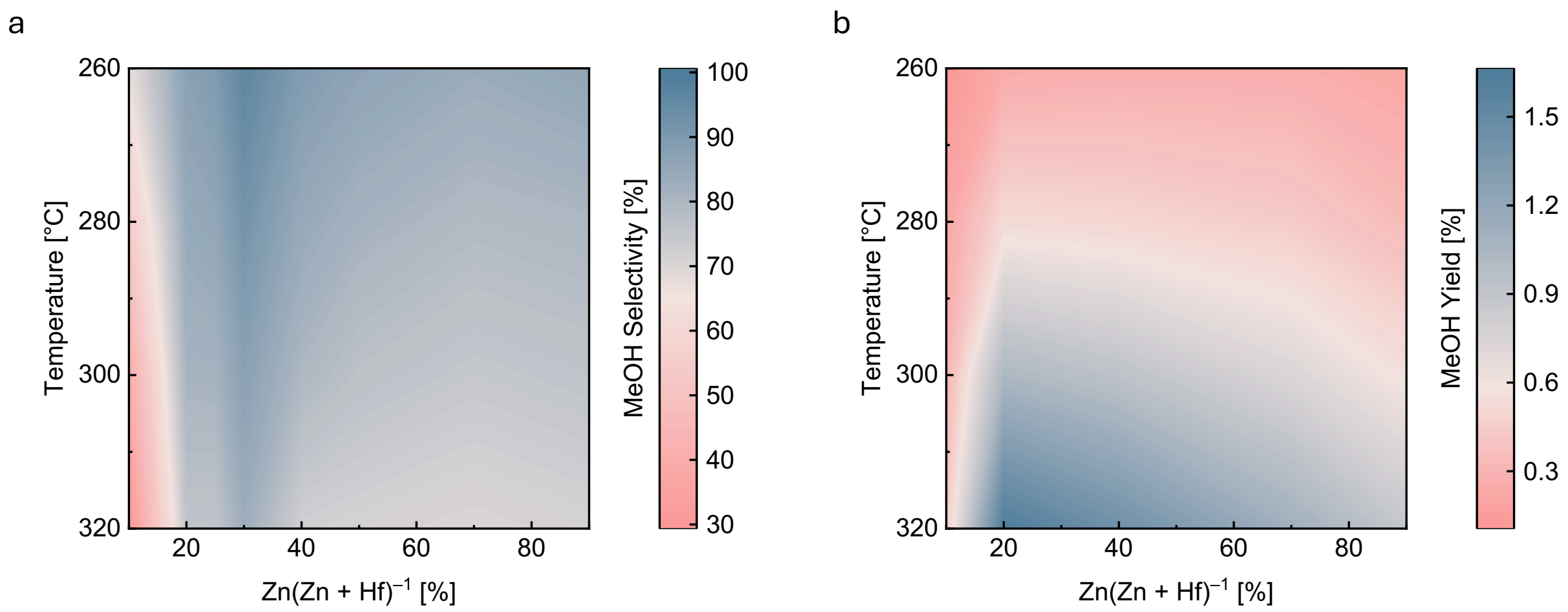

Figure S21: Methanol selectivity and yield of $ZnHfO_x$ catalysts for Zn contents ranging from 10 – 90 mol%. (a) Methanol selectivity and (b) methanol yield of $ZnHfO_x$ catalysts for Zn contents ranging from 10 – 90 mol% over a temperature range of 260 – 320 °C at 20 bar, H_2:CO_2:N_2 = 3:1:1, and 24 $L(g_{cat}h)^{-1}$.

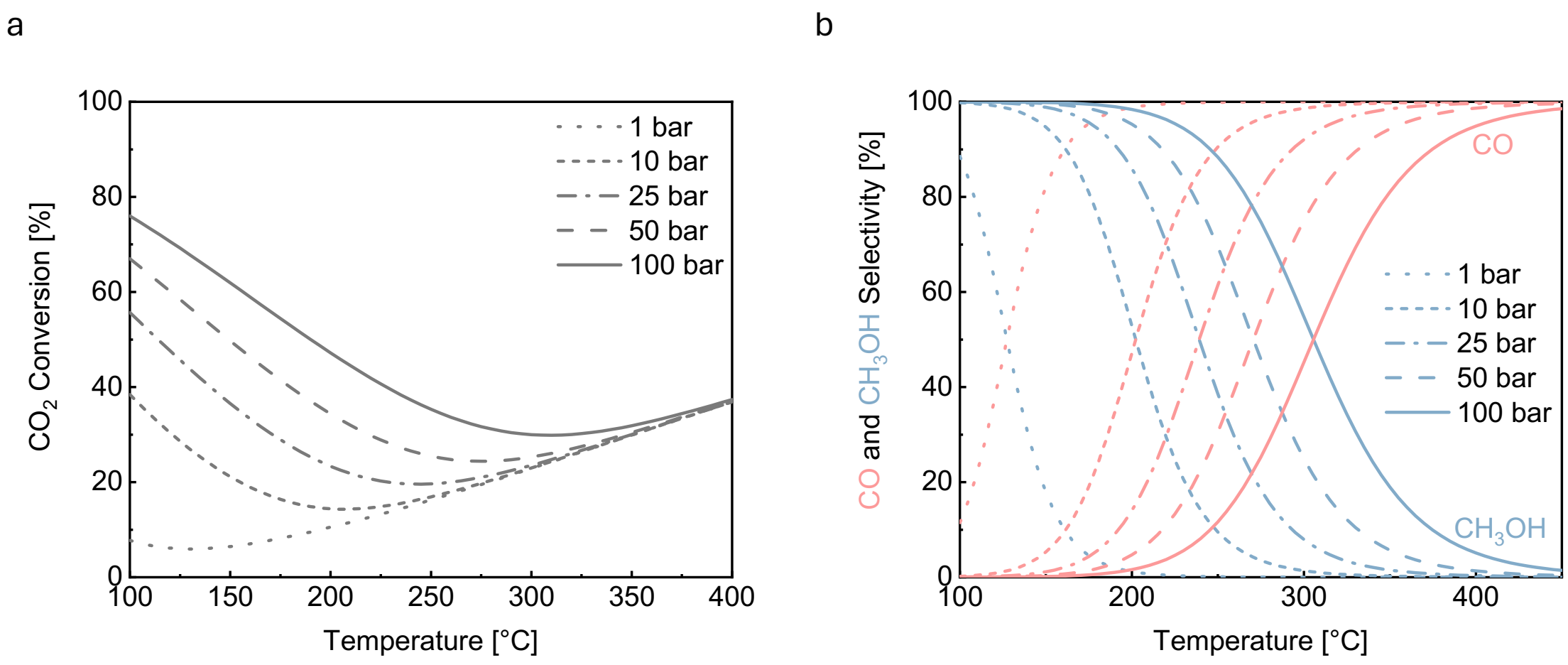

Figure S22: CO_2 conversion and product selectivities at thermodynamic equilibrium. (a) CO_2 conversion at thermodynamic equilibrium in the pressure range from 1 – 100 bar and temperature range from 100 – 400 °C. (b) CO and methanol selectivities at thermodynamic equilibrium in the pressure range from 1 – 100 bar and temperature range from 100 – 400 °C. Thermodynamic equilibrium was calculated by Gibbs free energy minimization considering the products of the RWGS reaction and the hydrogenation of CO_2 to methanol.

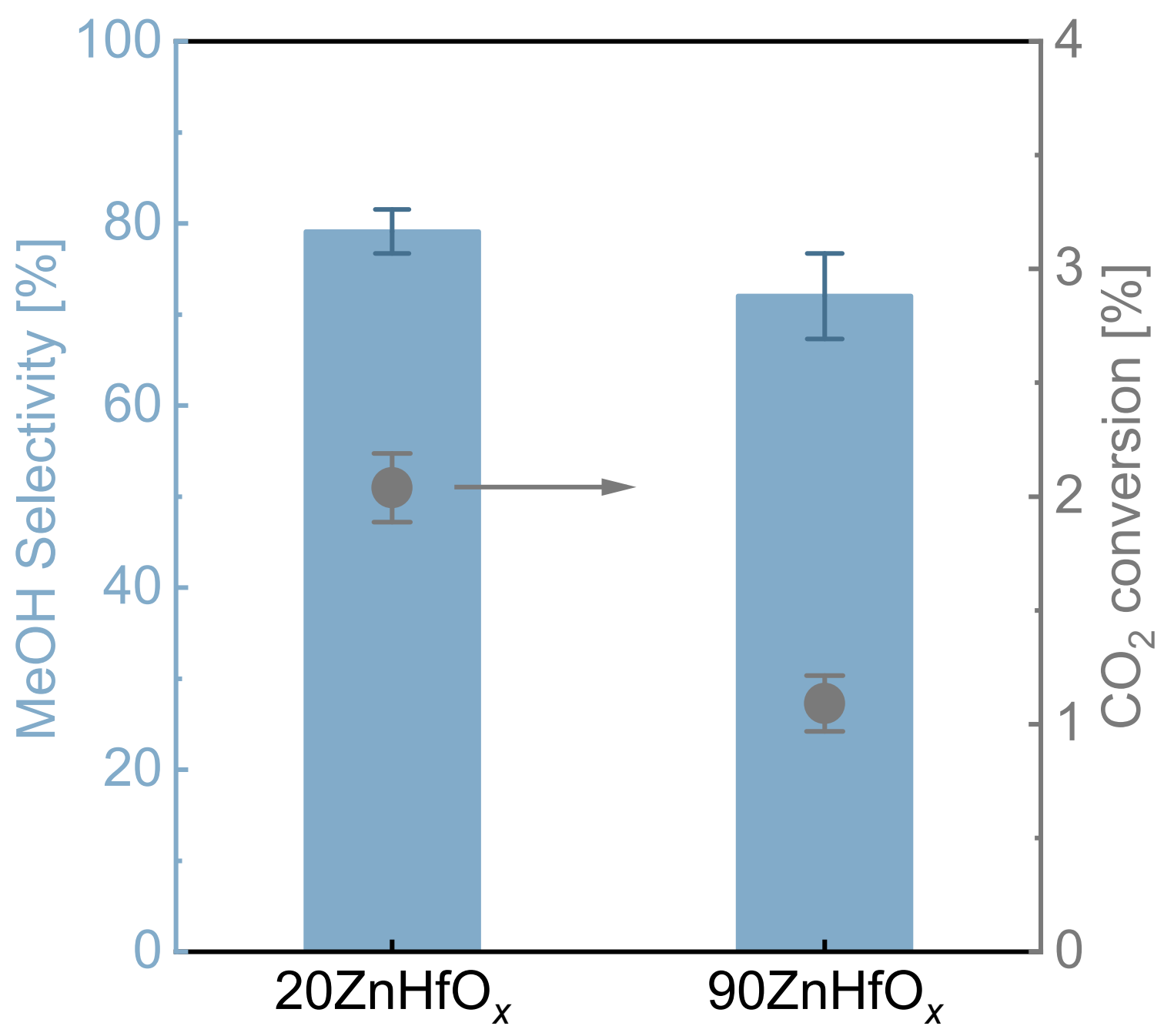

Figure S23: Reproducibility of catalytic data. Average methanol selectivity, and CO_2 conversion of 20ZnHfO_x and 90ZnHfO_x calculated from 3 individual catalytic tests. Error bars indicate the standard deviation from the mean. Catalytic tests were carried out at 320 °C, 20 bar, and GHSV = 24 L/(g_{cat}h).

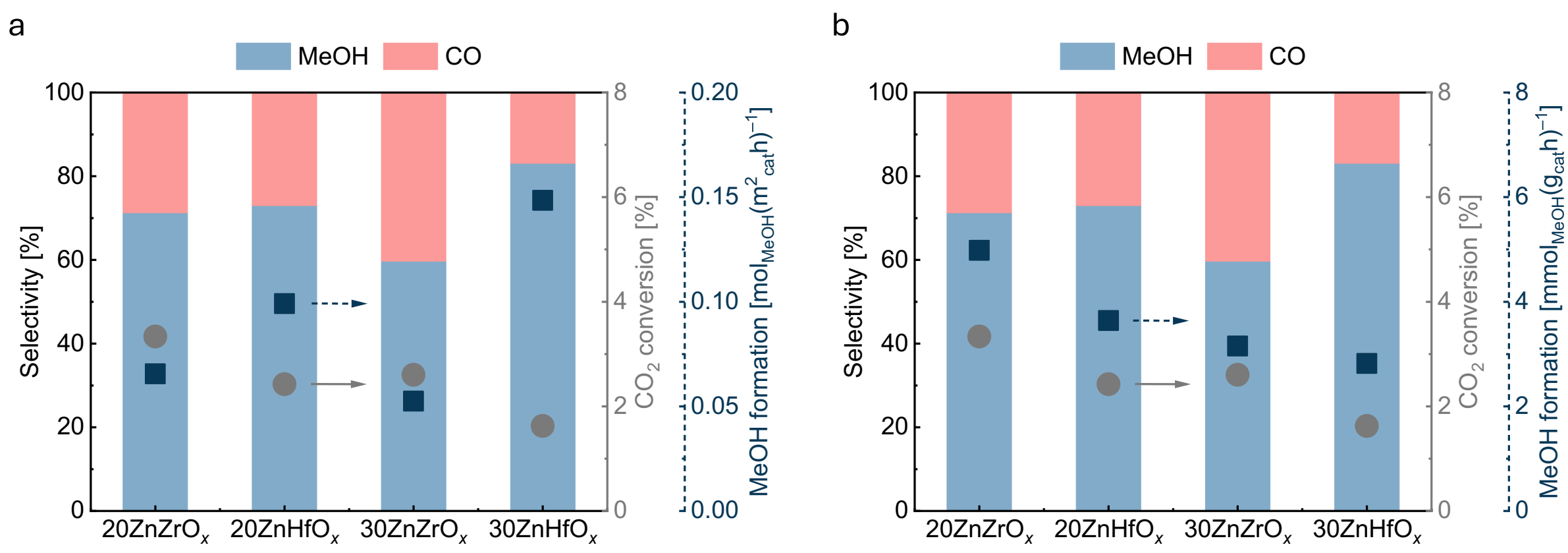

Figure S24: Catalytic performance metrics of $ZnHfO_x$ and $ZnZrO_x$ catalysts. CO_2 conversion, methanol selectivity, and (a) surface area-normalized methanol formation rate, (b) mass-normalized methanol formation rate of $ZnHfO_x$ and $ZnZrO_x$ containing 20 or 30 mol% Zn. Catalytic tests were carried out at 320 °C, 20 bar, and GHSV = 24 L/(g_{cat}h).

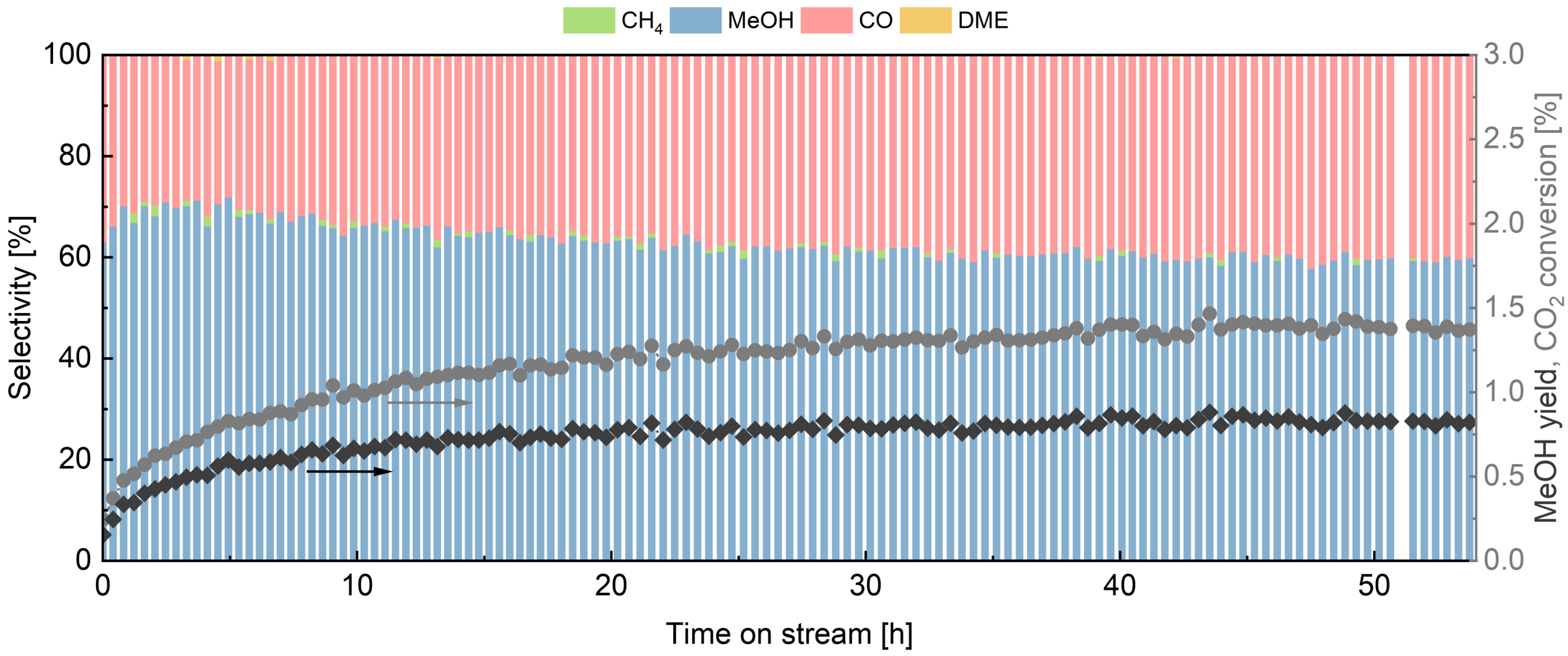

Figure S25: Stability test of 90ZnHfO$_x$. CO_2 conversion, product selectivities and methanol yield of 90ZnHfO$_x$ over 55 h of time on stream at 320 °C, 20 bar, and GHSV = 24 L/(g_{cat}h).

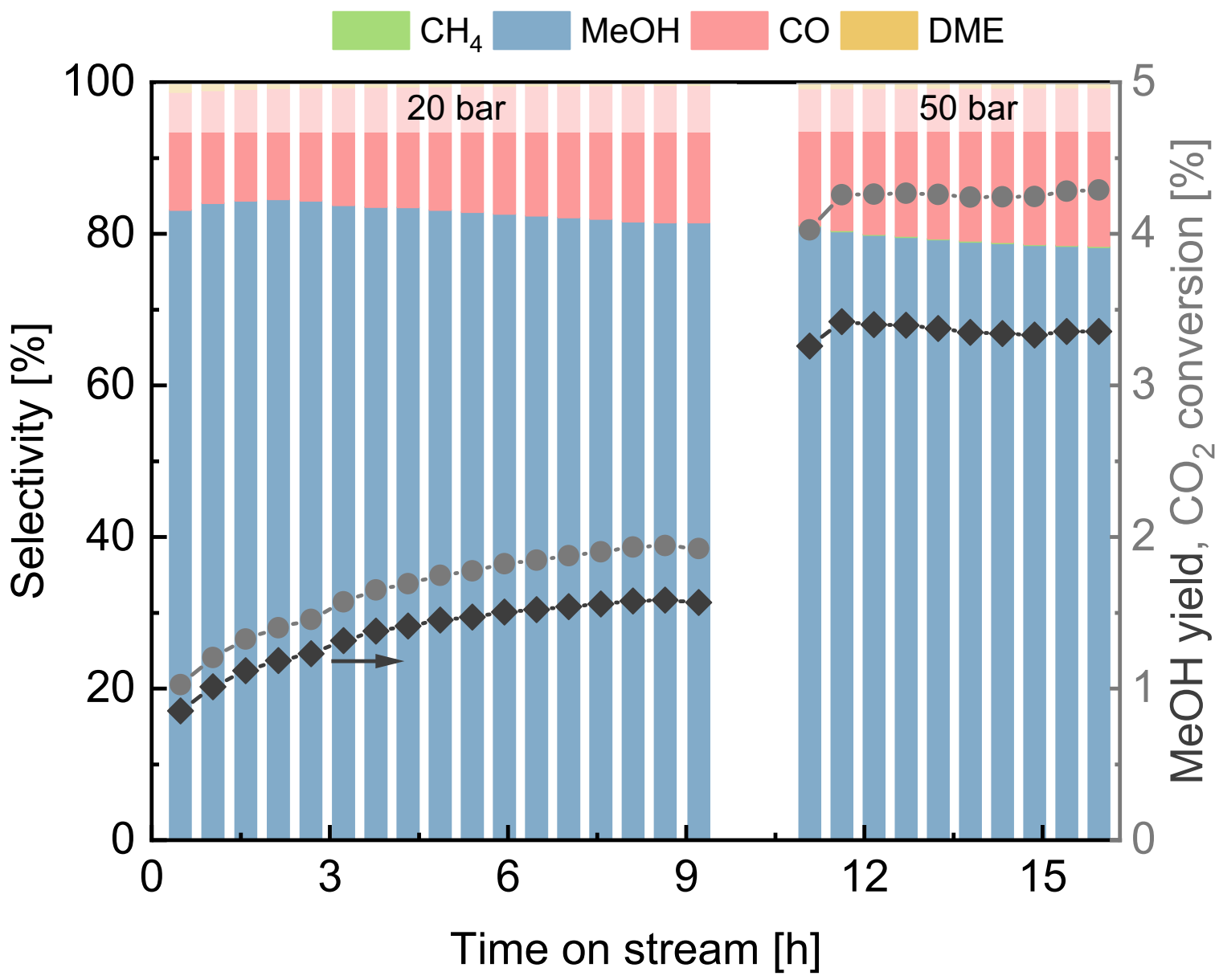

Figure S26: High pressure catalytic performance assessment of 20ZnHfO$_x$. 20ZnHfO$_x$ at 20 and 50 bar, 320 °C, H_2:CO_2:N_2 = 3:1:1, and 24 L$(g_{cat}h)^{-1}$.

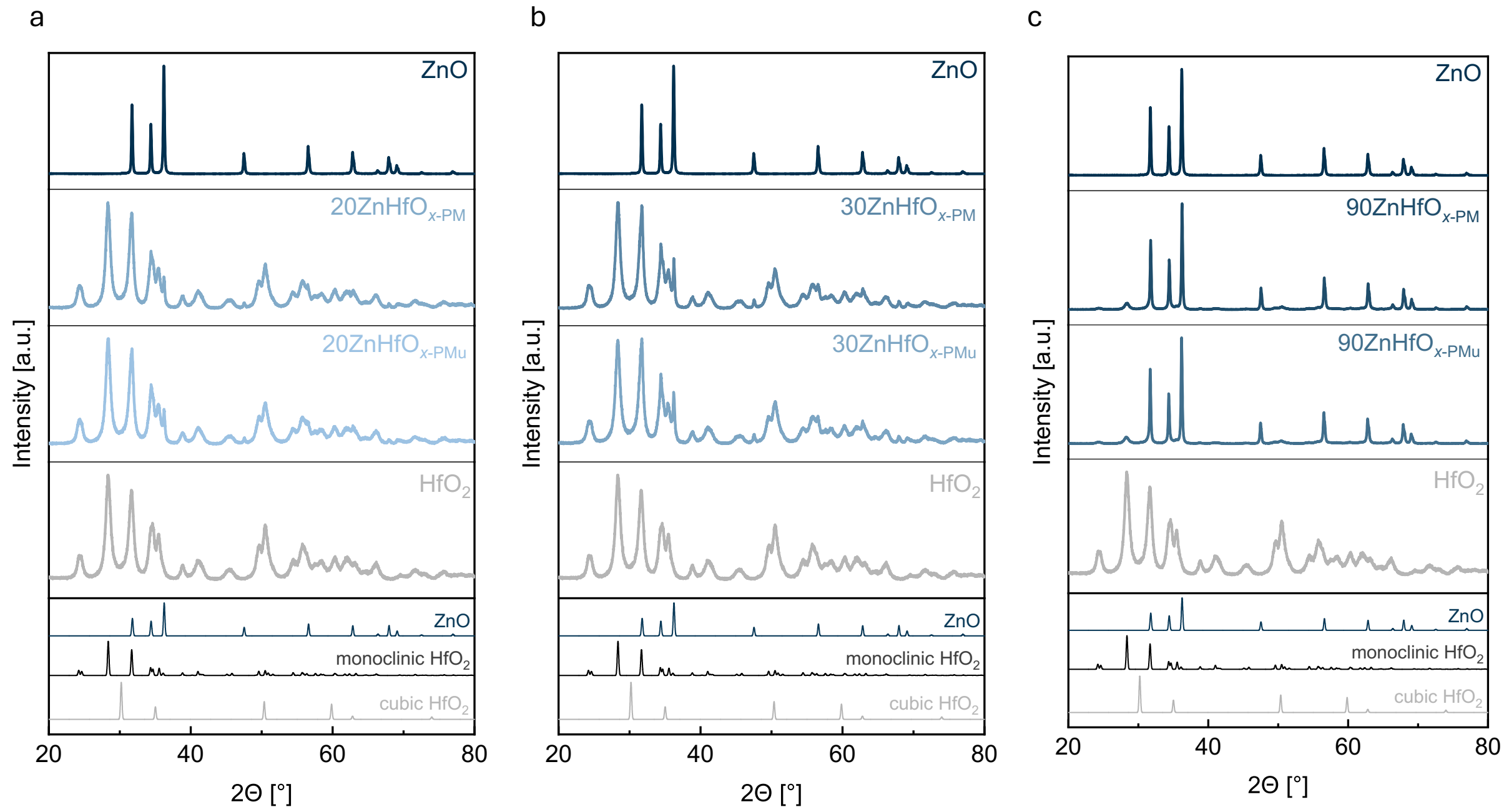

Figure S27: XRD patterns of as-prepared reference $ZnHfO_x$ catalysts. XRD patterns of (a) $20ZnHfO_x$, (b) $30ZnHfO_x$, and (c) $90ZnHfO_x$ synthesized by physical mixing (PM) of ZnO and ZrO_2; the respective diffraction patterns are included in the individual graphs. The graphs contain diffraction patterns of the physically mixed catalysts before (PMu) and after calcination (PM).

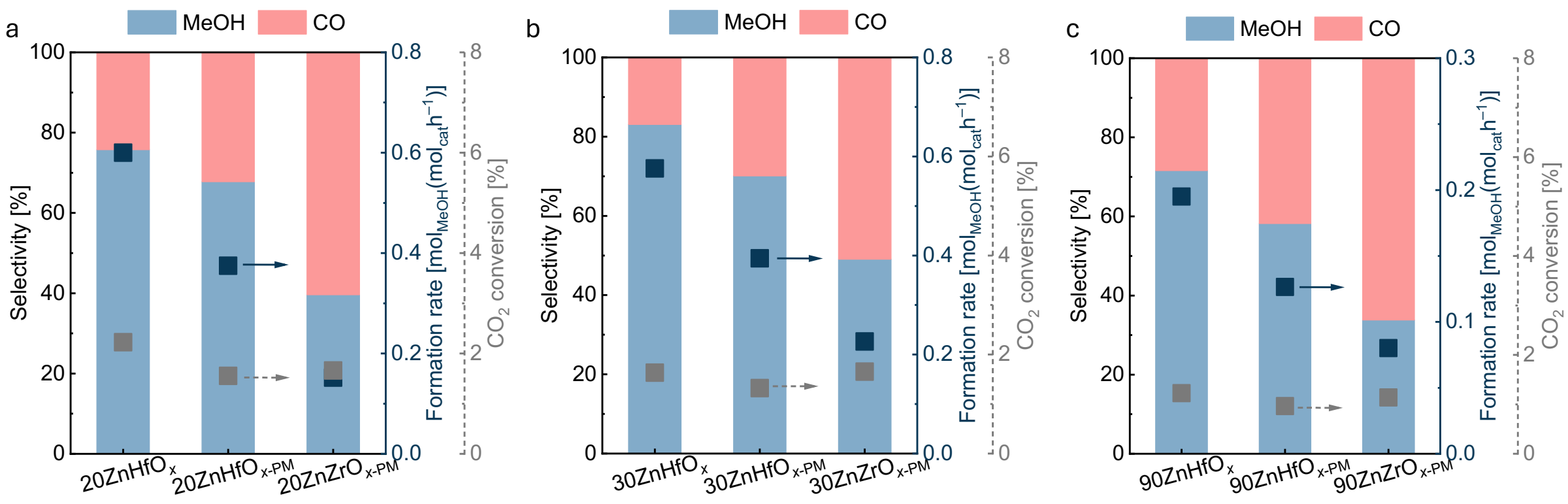

Figure S28: Catalytic performance of ZnHfO$_{x\text{-PM}}$ and ZnZrO$_{x\text{-PM}}$ catalysts. Comparison of the catalytic performances of co-precipitated ZnHfO$_x$ with that of ZnHfO$_{x\text{-PM}}$ and ZnZrO$_{x\text{-PM}}$ with Zn contents of (a) 20 mol%, (b) 30 mol%, and (c) 90 mol% at 20 bar, 320 °C, H_2:CO_2:N_2 = 3:1:1, and 24 L(g_{cat}h)$^{-1}$.

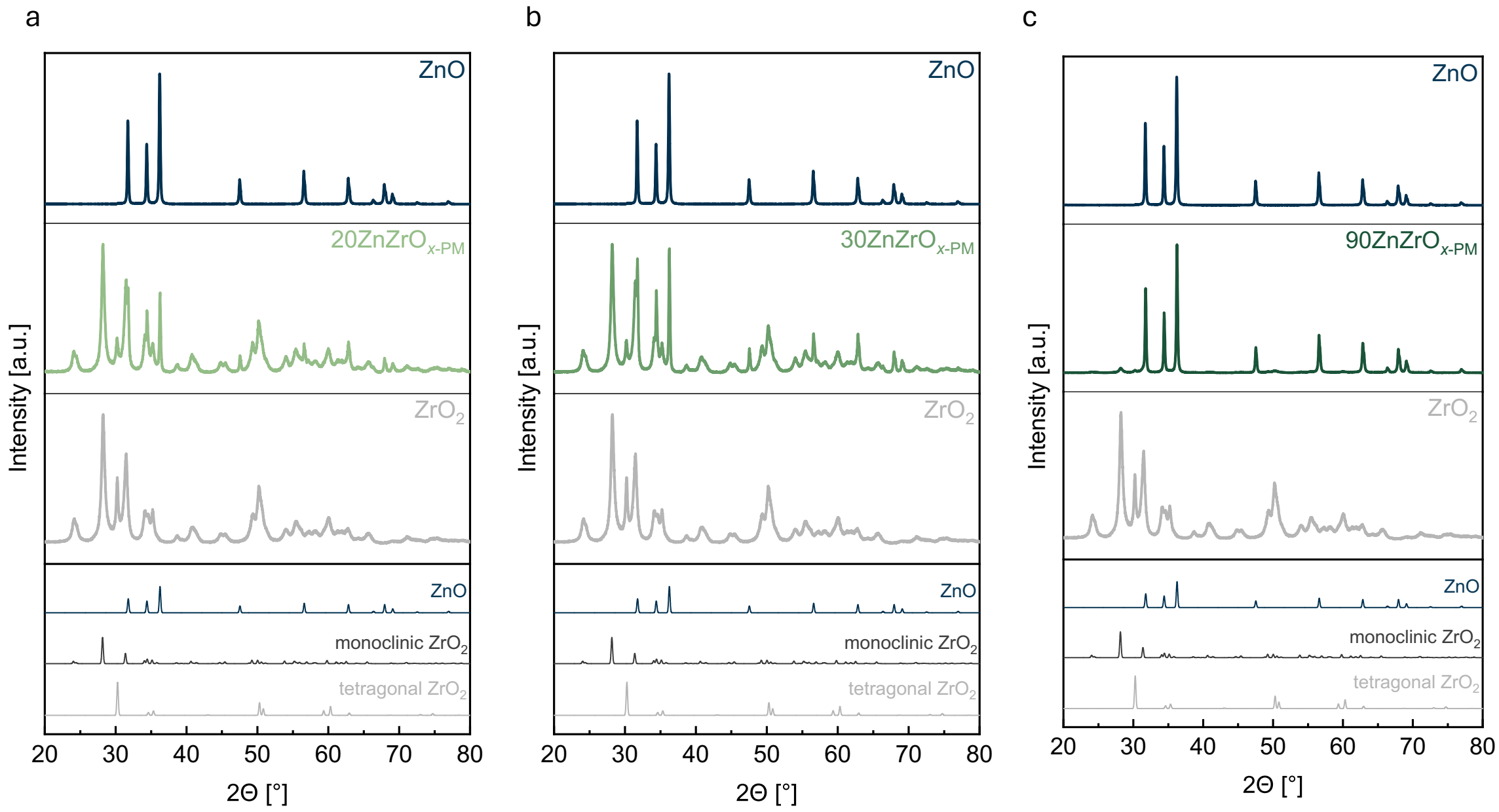

Figure S30: XRD patterns of as-prepared ZnZrO$_{x\text{-PM}}$ catalysts. XRD patterns of (a) 20ZnZrO$_{x\text{-PM}}$, (b**)** 20ZnZrO$_{x\text{-PM}}$, and (c**)** 20ZnZrO$_{x\text{-PM}}$ catalysts synthesized by physical mixing of ZnO and ZrO_2; the respective diffraction patterns are included in the individual graphs.

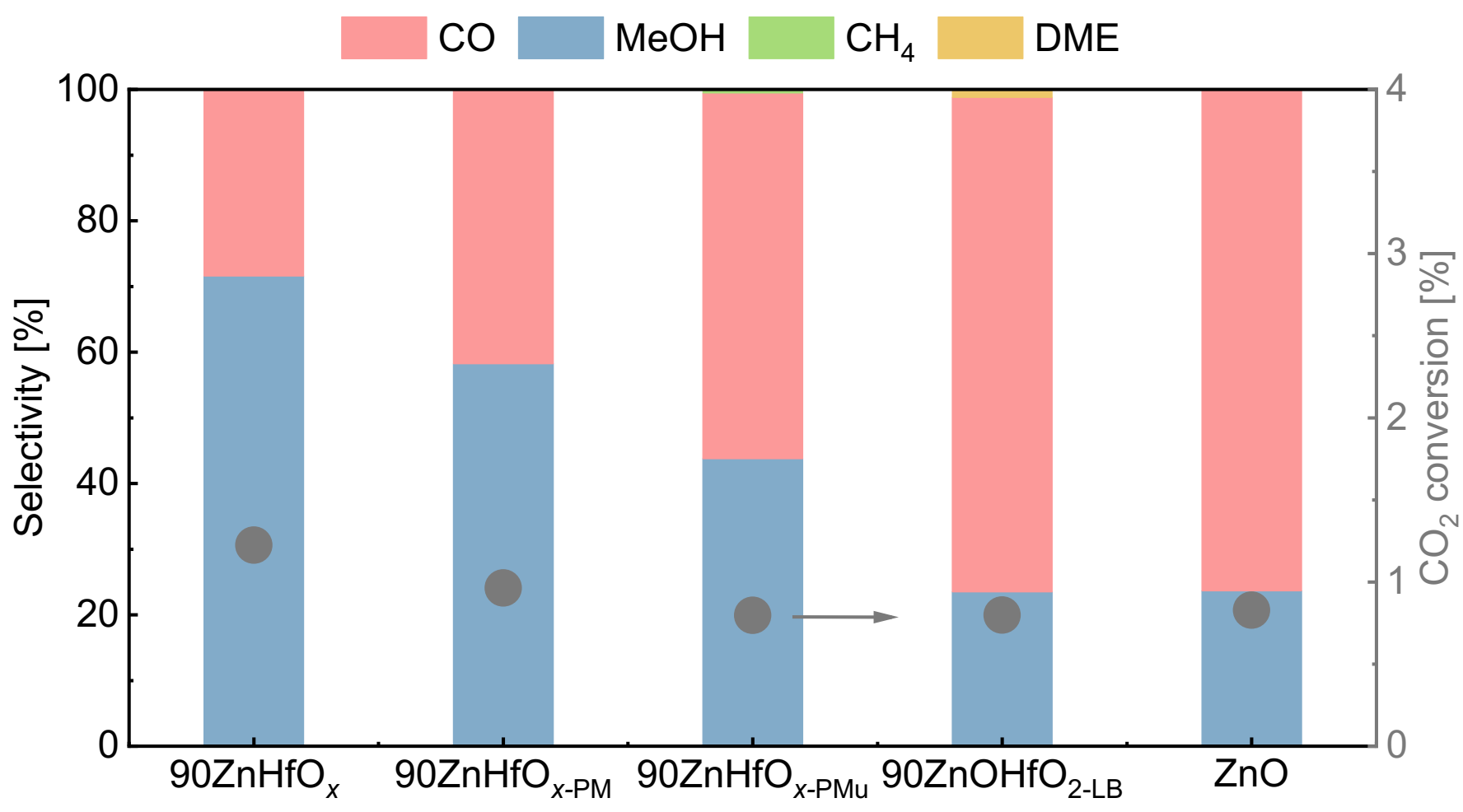

Figure S31: Catalytic performance of differently prepared 90ZnHfO$_x$ catalysts. Comparison of the CO_2 conversion and methanol selectivity of a co-precipitated catalyst (90ZnHfO$_x$), a calcined (90ZnHfO$_{x\text{-PM}}$) and uncalcined (90ZnHfO$_{x\text{-PMu}}$) physical mixture, a layered bed of ZnO and HfO_2, each with a molar ZnO:HfO_2 ratio of 90:10, and pure ZnO. The catalytic tests were performed at 20 bar, 320 °C, H_2:CO_2:N_2 = 3:1:1, and 24 L(g_{cat}h)$^{-1}$.

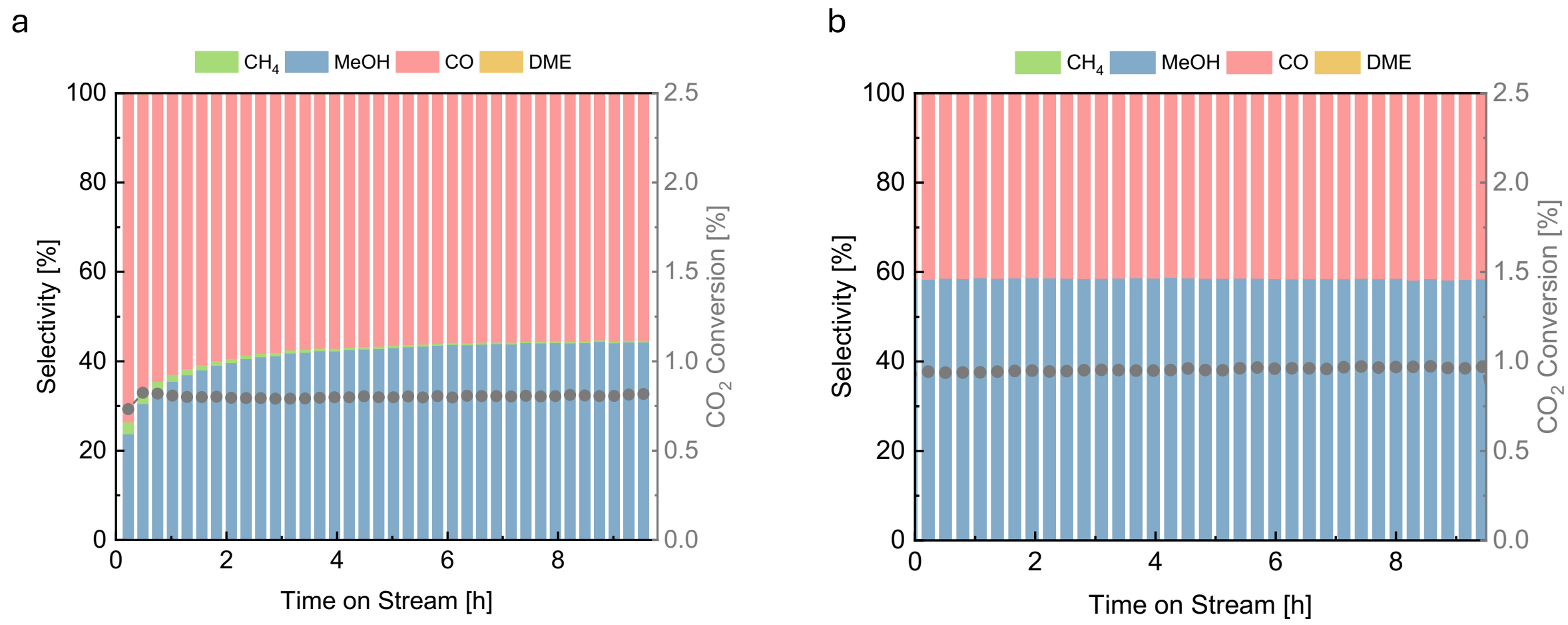

Figure S32: Catalytic performance of 90ZnHfO$_{x\text{-PM}}$ before and after calcination. The catalytic performance of 90ZnHfO$_{x\text{-PM}}$ at 20 bar, 320 °C, H_2:CO_2:N_2 = 3:1:1, and 24 L(g_{cat}h)$^{-1}$ (a) before and (b) after calcination at 580 °C for 3 hours.

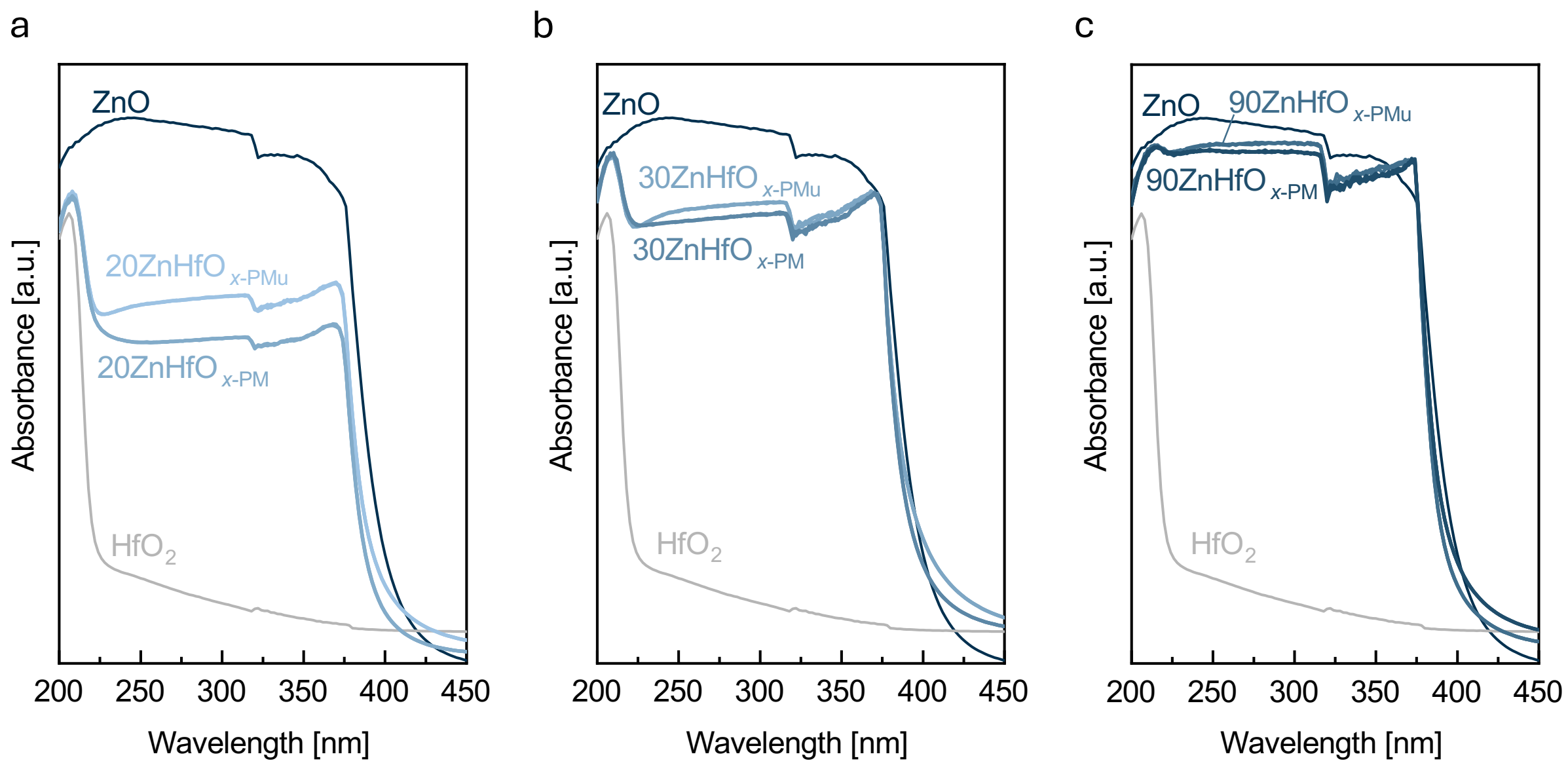

Figure S33: UV-Vis spectroscopy of ZnHfO$_{x\text{-PM}}$ catalysts before and after calcination. UV-Vis spectra of ZnHfO$_{x\text{-PM}}$ catalysts before (PMu) and after (PM) calcination at 580 °C for 3 h for Zn contents of (a) 20 mol%, (b) 30 mol%, and (c) 90 mol%.

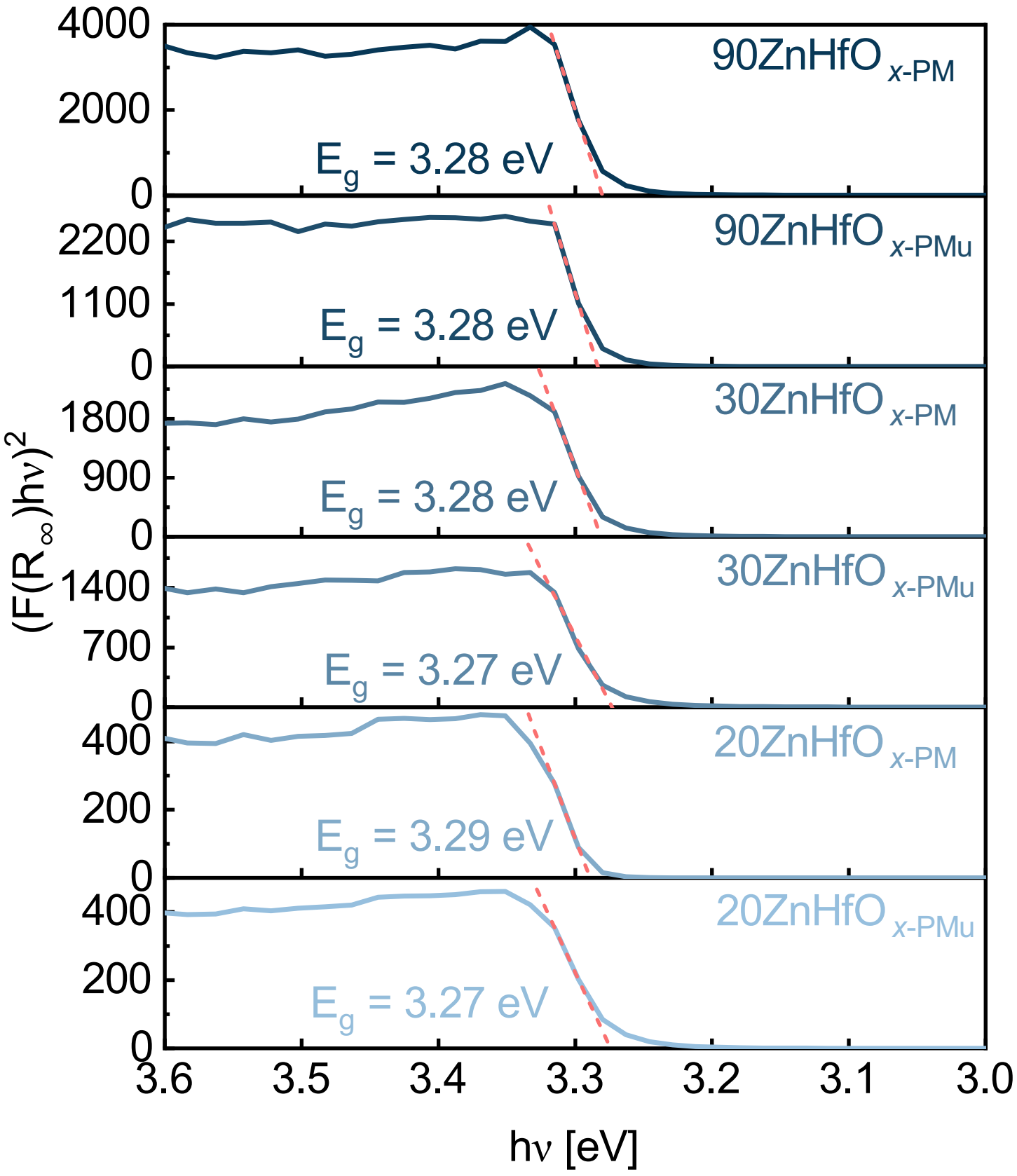

Figure S34: Tauc plots of ZnHfO$_{x\text{-PM}}$ UV-vis spectra with Zn contents ranging from 20 – 90 mol%. Tauc plots of uncalcined (PMu) and calcined (PM) ZnHfO$_{x\text{-PM}}$ UV-Vis spectra with Zn contents ranging from 20 – 90 mol%. The intersection of the linear fit of the absorbance step (red dashed line) with the ordinate yields the energy of the bandgap in eV.

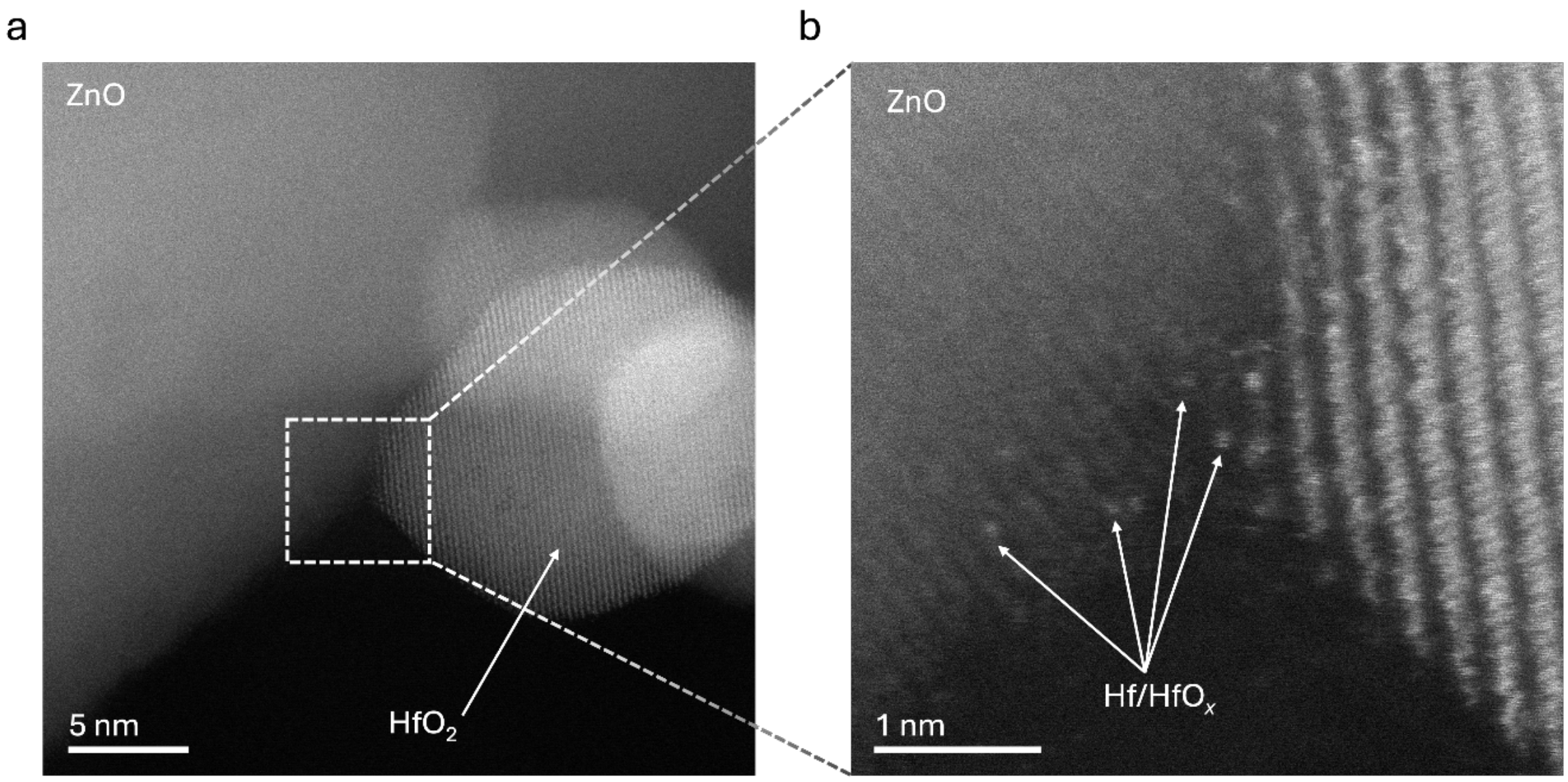

Figure S35: HAADF-STEM images of 90ZnHfO$_{x\text{-PM}}$. (a) HAADF-STEM image of 90ZnHfO$_{x\text{-PM}}$ showing different domains/particles of bulk ZnO and HfO_2. (b) Enlarged HAADF-STEM image of 90ZnHfO$_{x\text{-PM}}$ at the interface of ZnO and HfO_2 revealing Hf/HfO$_x$ clusters that migrated onto the bulk ZnO particle.

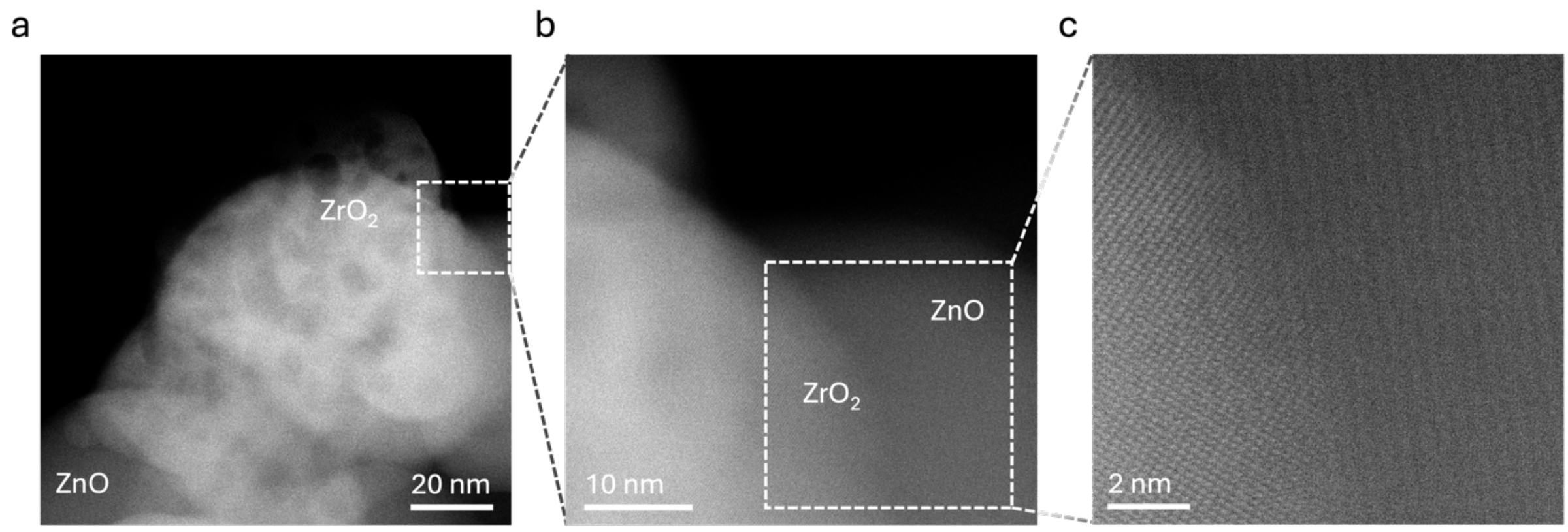

Figure S36: ADF-STEM images of 90ZnZrO$_{x\text{-PM}}$. (a) ADF-STEM image of 90ZnZrO$_{x\text{-PM}}$ showing the different domains/particles of ZnO and ZrO_2. (b) and (c) enlarged ADF-STEM images of 90ZnZrO$_{x\text{-PM}}$ revealing a ZnO surface devoid of Zr/ZrO_x clusters at the ZnO/ZrO_2 interface demonstrating the absence of Zr migration.

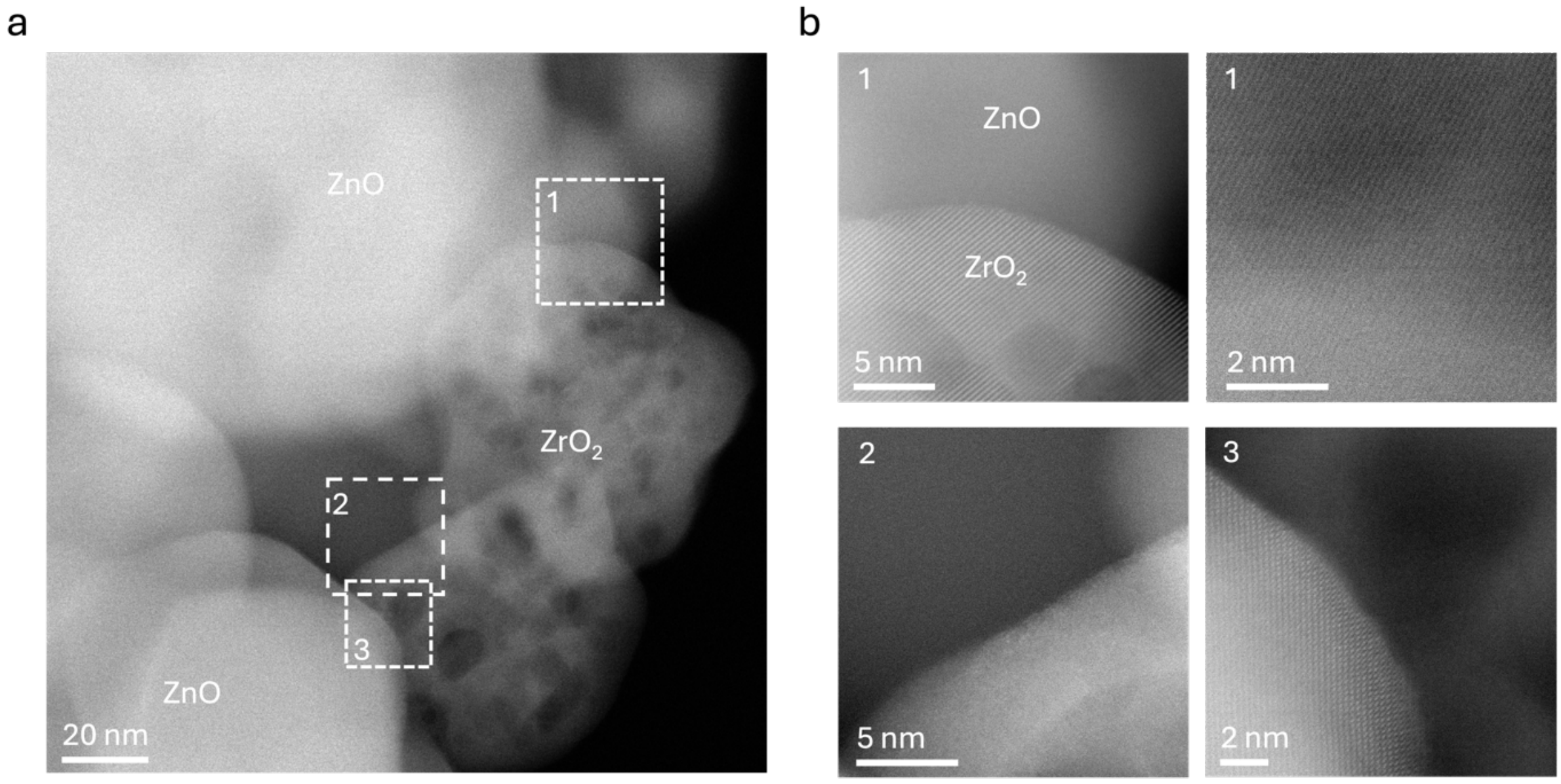

Figure S37: ADF-STEM images of 90ZnZrO$_{x\text{-PM}}$. (a) ADF-STEM image of 90ZnZrO$_{x\text{-PM}}$ showing the different domains/particles of ZnO and ZrO_2. (b) Enlarged ADF-STEM images of 90ZnZrO$_{x\text{-PM}}$ (according to the respective numbered frames) reveal weak attachment/interactions between the particles of the individual oxides and ZnO surfaces devoid of Zr/ZrO$_x$ clusters.

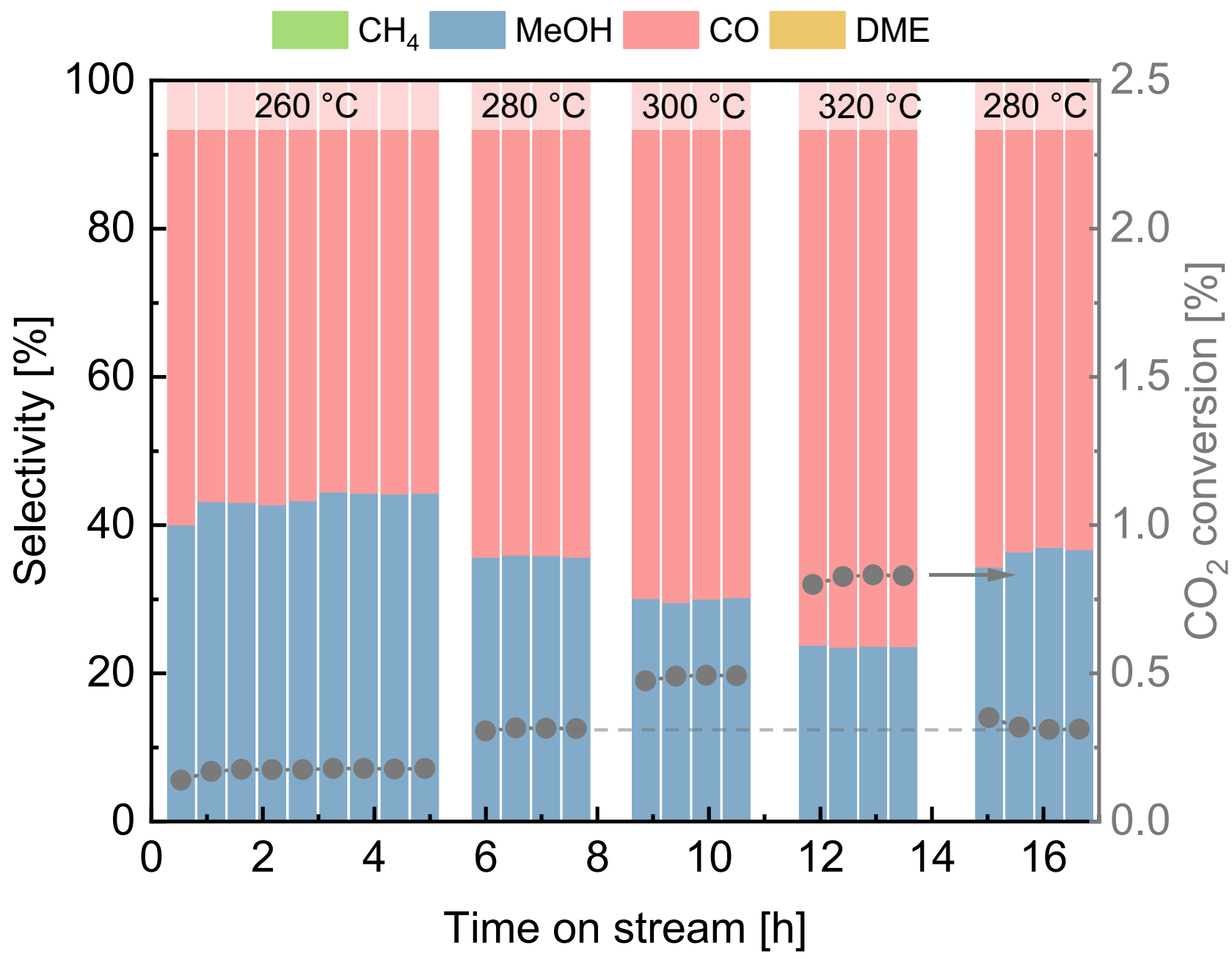

Figure S38: Catalytic performance of ZnO with time on stream. Catalytic performance of ZnO with time on stream at 20 bar, 320 °C, H_2:CO_2:N_2 = 3:1:1, and 24 L(g_{cat}h)$^{-1}$.

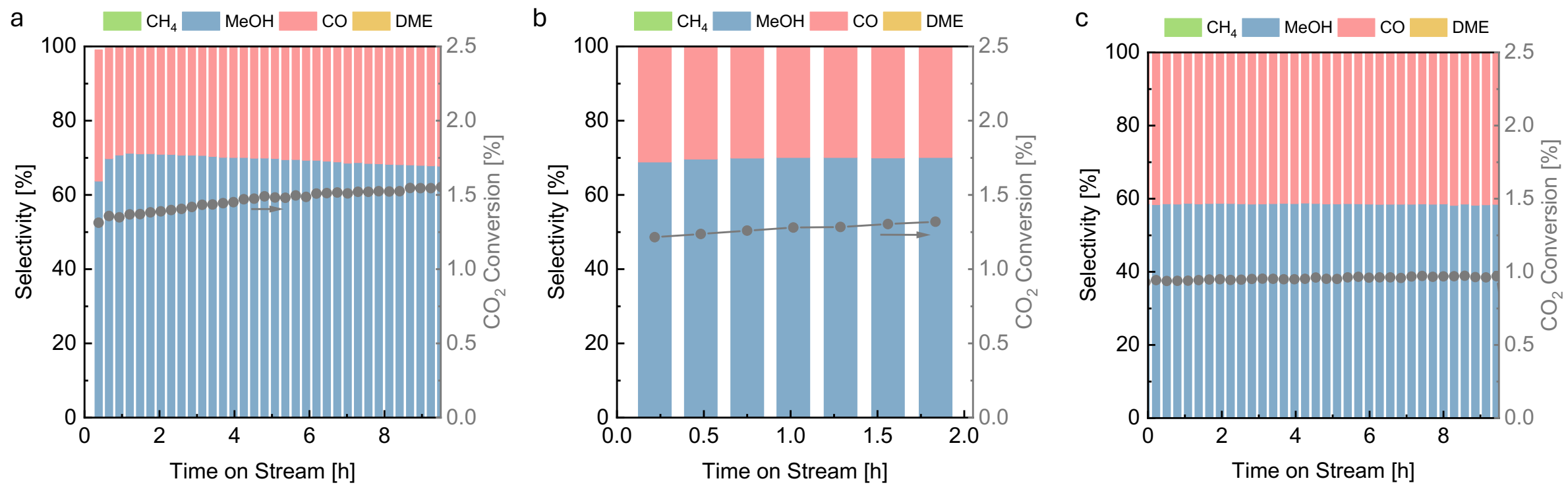

Figure S39: Catalytic performance of $ZnHfO_{x\text{-}PM}$ catalysts with time on stream. Catalytic performance of (a) $20ZnHfO_{x\text{-}PM}$, (b) $30ZnHfO_{x\text{-}PM}$, and (c) $90ZnHfO_{x\text{-}PM}$ with time on stream at 20 bar, 320 °C, H_2:CO_2:N_2 = 3:1:1, and 24 $L(g_{cat}h)^{-1}$.

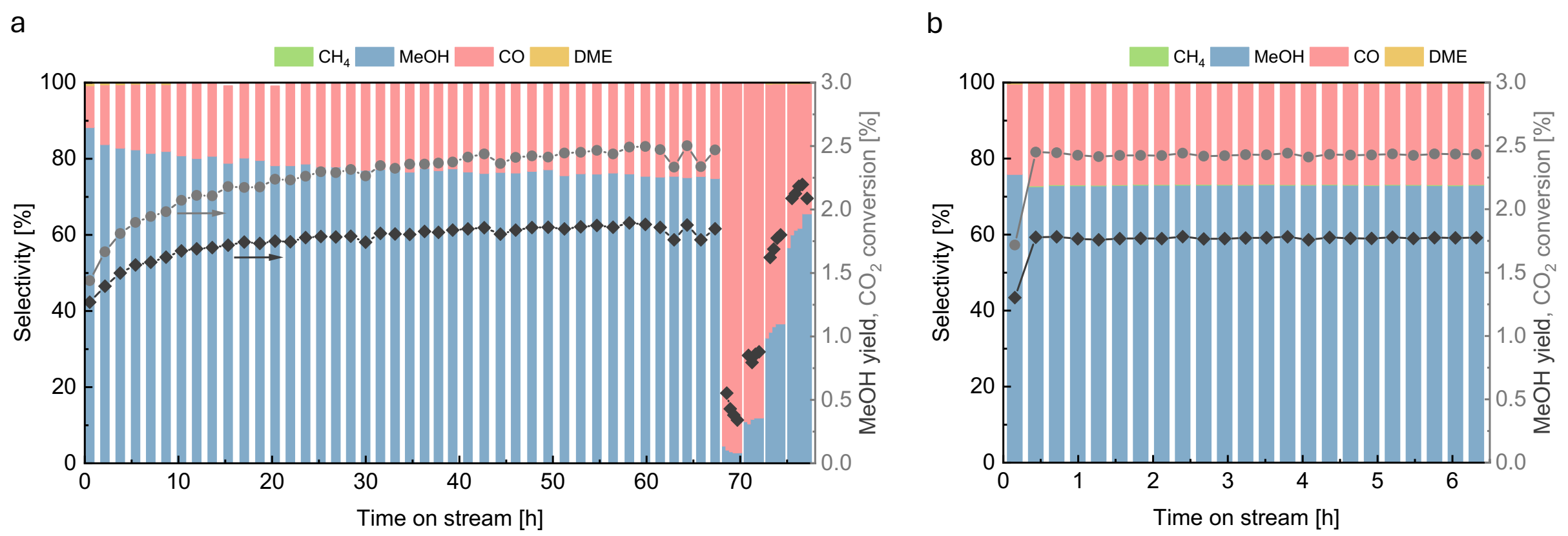

Figure S40: Long-term stability test of 20ZnHfO$_x$. (a) Longterm performance assessment of 20ZnHfO$_x$ at 20 bar, 320 °C, H_2:CO_2:N_2 = 3:1:1 and 24 L(g_{cat}h)$^{-1}$ for 68 h including a consecutive temperature sweep from 400 to 340 °C, and (b) a consecutive, second catalytic test of the same catalyst at 20 bar, 320 °C, H_2:CO_2:N_2 = 3:1:1, and 24 L(g_{cat}h)$^{-1}$ to demonstrate its stability.

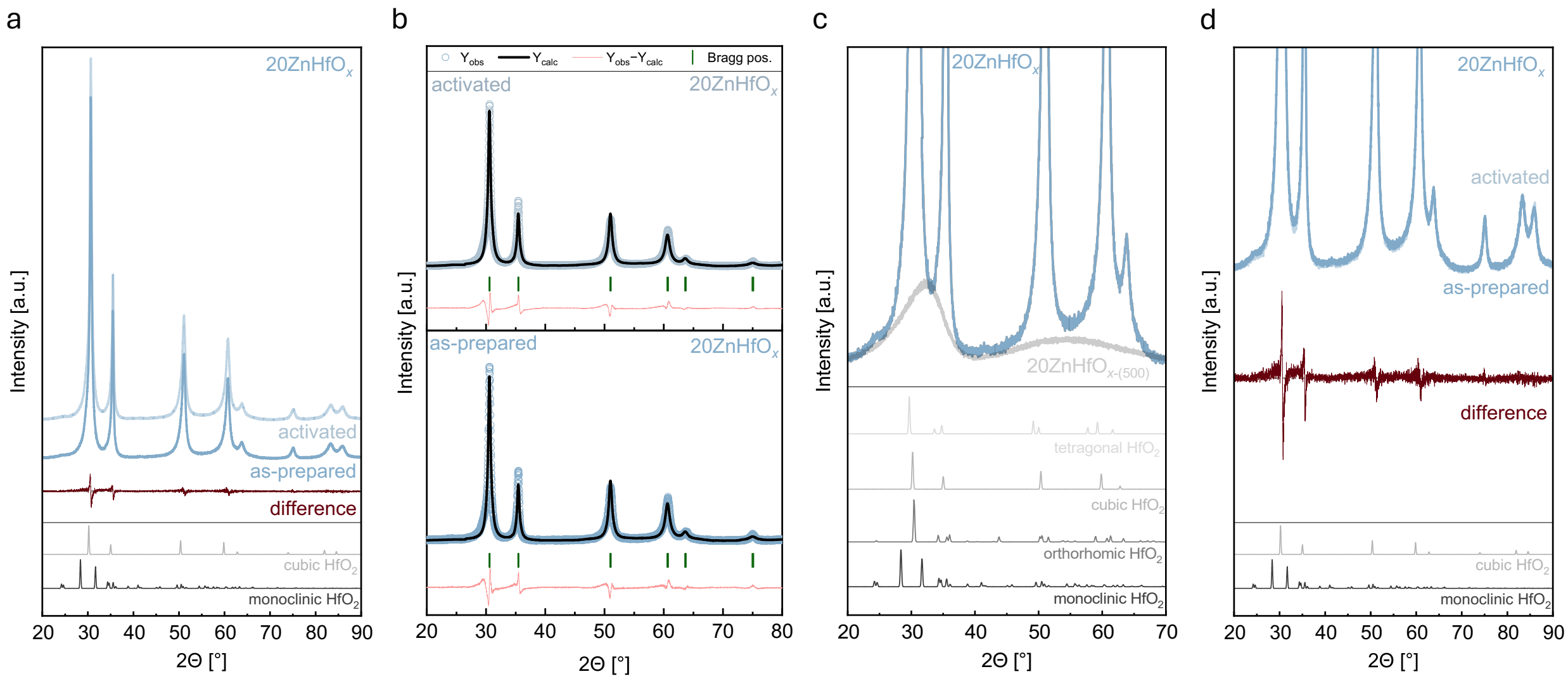

Figure S41: Post catalytic XRD analysis of 20ZnHfO*x*. (a) Comparison of XRD patterns of as-prepared and activated 20ZnHfO*x*, as well as the calculated difference between both patterns. (b) Rietveld refinements of as-prepared and activated 20ZnHfO*x*. (c) Overlay of the XRD patterns of 20ZnHfO*x* and amorphous 20ZnHfO*x*-(500). (d) Overlay of the XRD patterns of as-prepared and activated 20ZnHfO*x*.

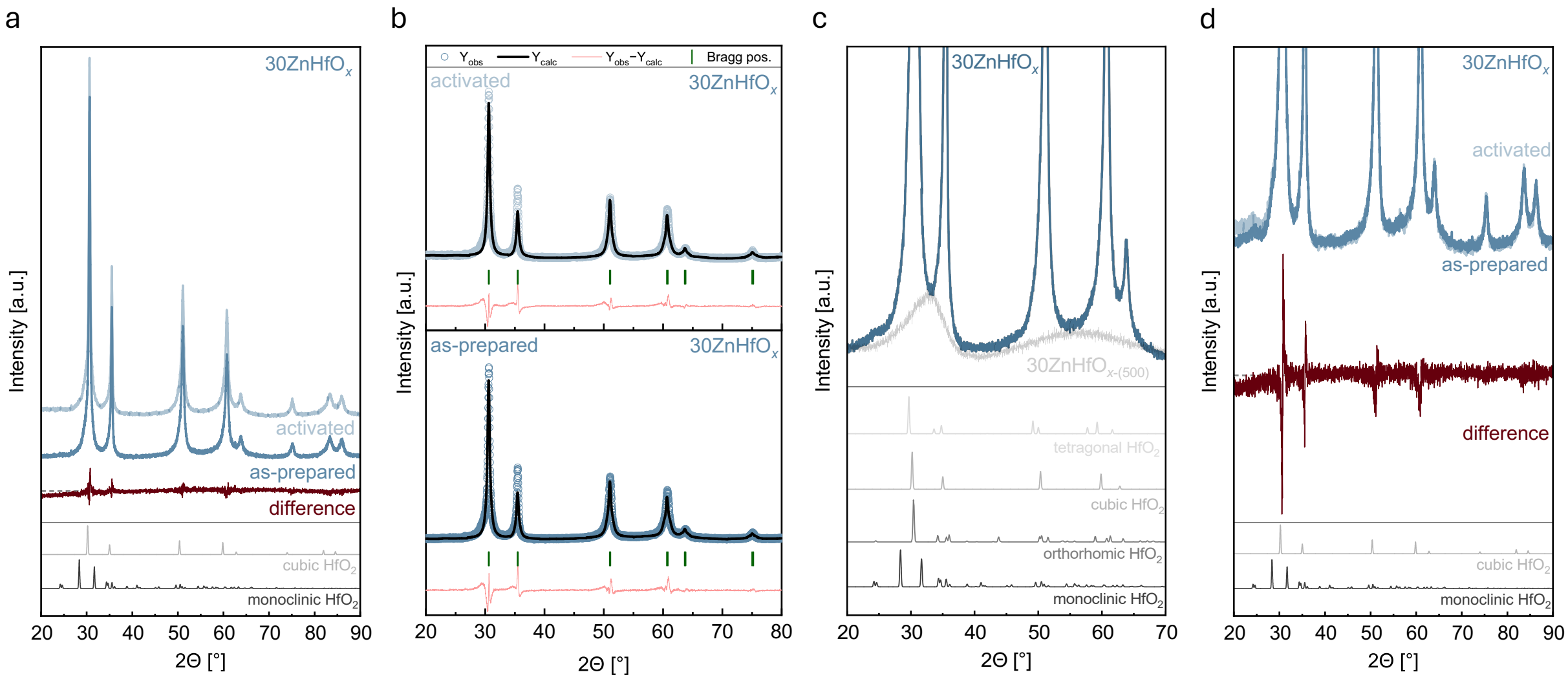

Figure S42: Post catalytic XRD analysis of 30ZnHfO$_x$. (a) Comparison of XRD patterns of as-prepared and activated 30ZnHfO$_x$, as well as the calculated difference between both patterns. (b) Rietveld refinements of as-prepared and activated 30ZnHfO$_x$. (c) Overlay of the XRD patterns of 30ZnHfO$_x$ and amorphous 30ZnHfO$_{x\text{-}(500)}$. (d) Overlay of the XRD patterns of as-prepared and activated 20ZnHfO$_x$.

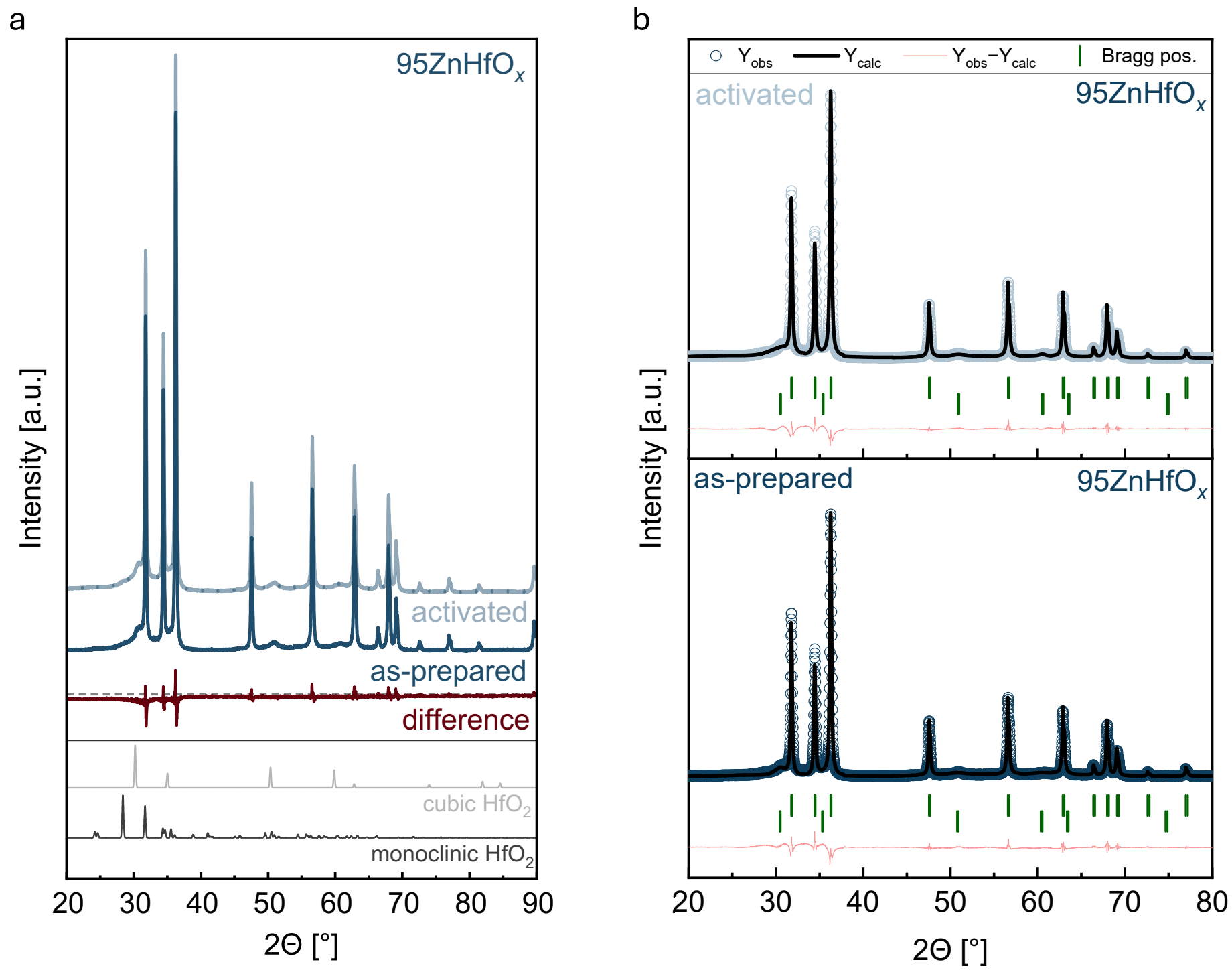

Figure S43: Post catalytic XRD analysis of 30ZnHfO$_x$. (a) Comparison of XRD patterns of as-prepared (fresh) and activated (spent) 95ZnHfO$_x$, as well as the calculated difference between both patterns. (b) Rietveld refinements of as-prepared and activated 95ZnHfO$_x$.

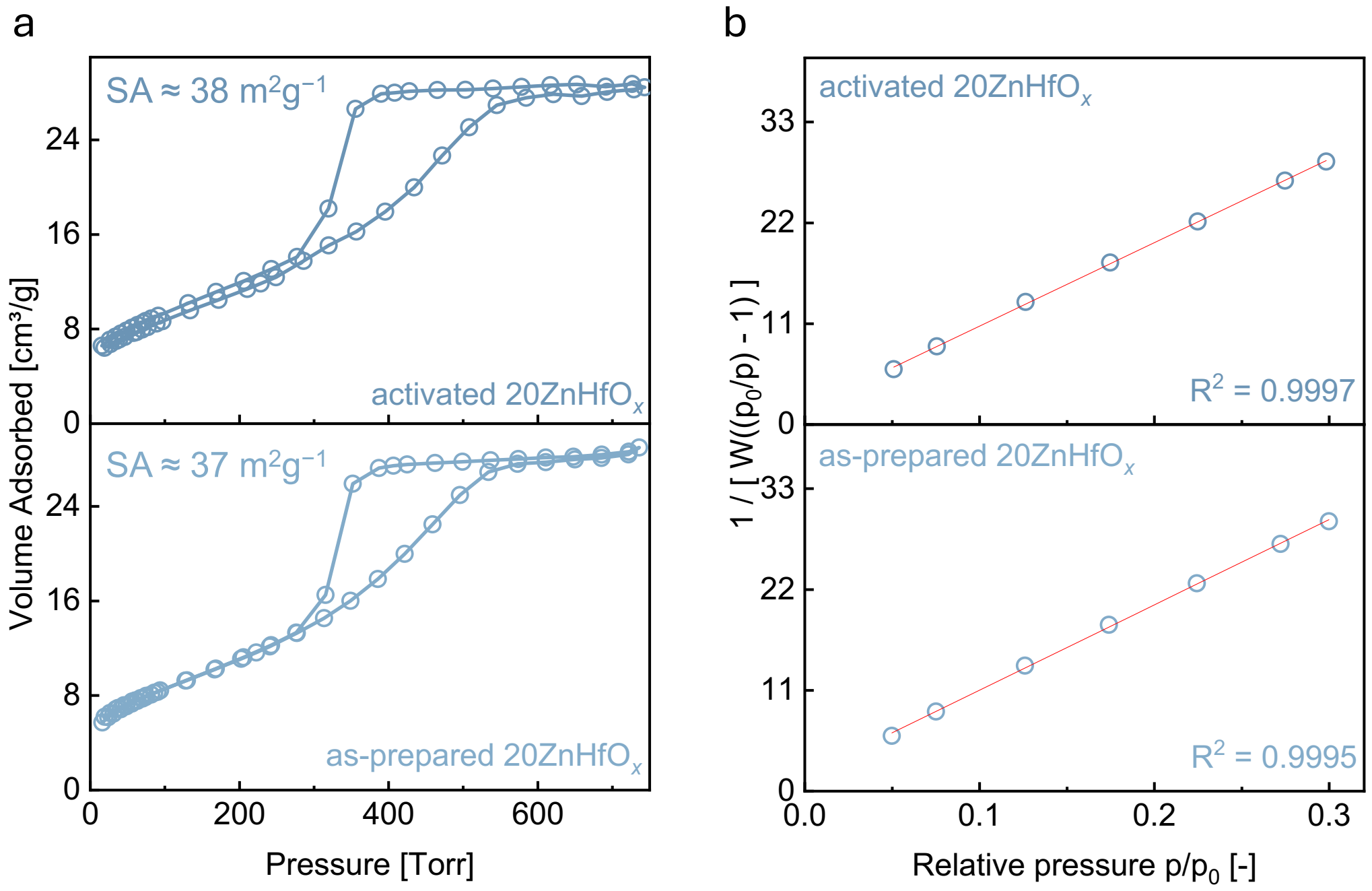

Figure S44: N_2 physisorption of as-prepared and activated 20ZnHfO$_x$ catalysts. (a) N_2 physisorption isotherms of as-prepared and activated 20ZnHfO$_x$ catalysts. The surface areas (SA) are displayed in the respective frames of the isotherms. (b) BET fits to obtain the specific surface area of as-prepared and activated 20ZnHfO$_x$ catalysts.

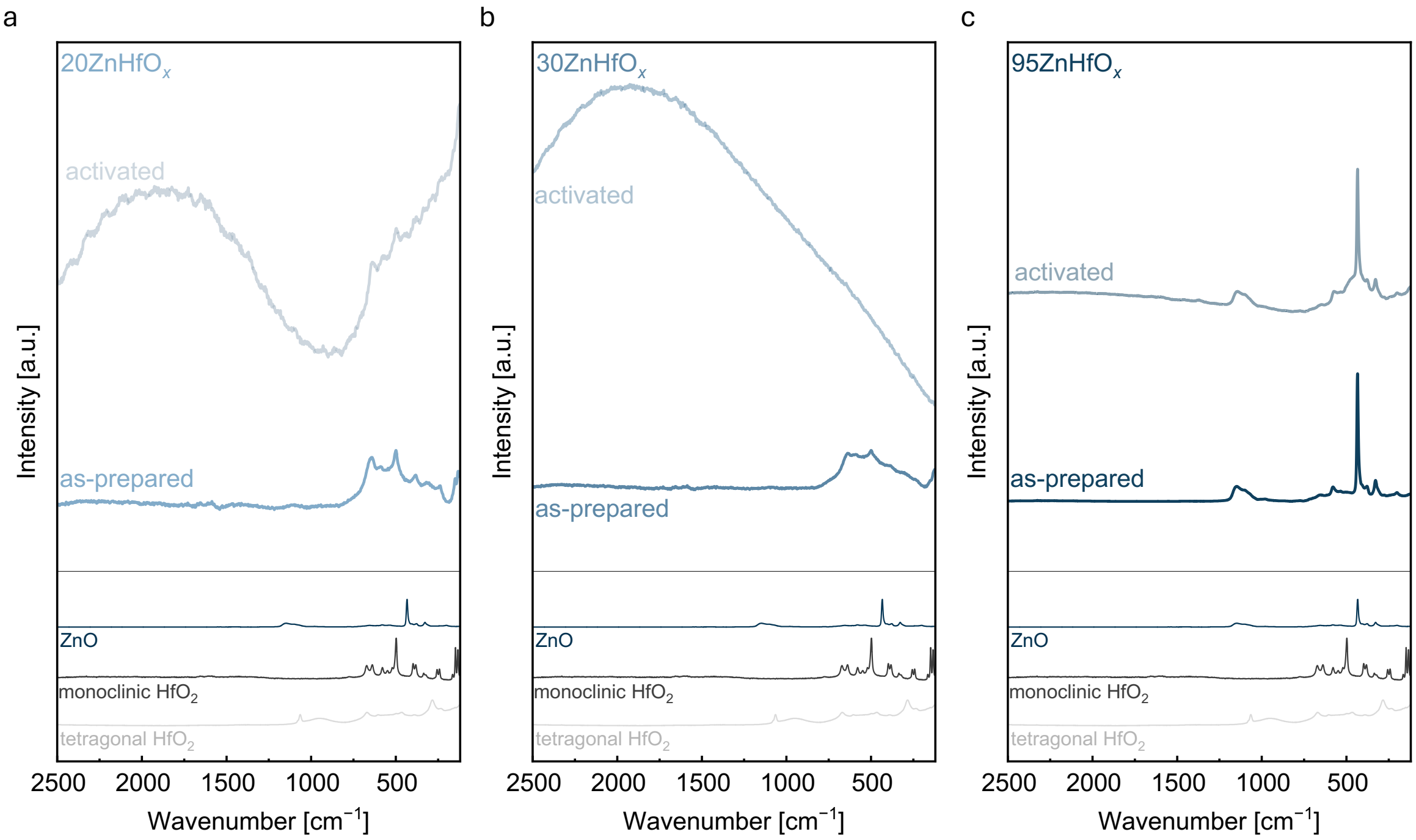

Figure S45: Post catalytic Raman spectroscopy analysis of ZnHfO$_x$. Raman spectra of as-prepared (fresh) and activated (spent) (a) 20ZnHfO$_x$, (b) 30ZnHfO$_x$, and (c) 95ZnHfO$_x$ catalysts. Every Raman spectrum is an average of 100 collected spectra of each catalyst.

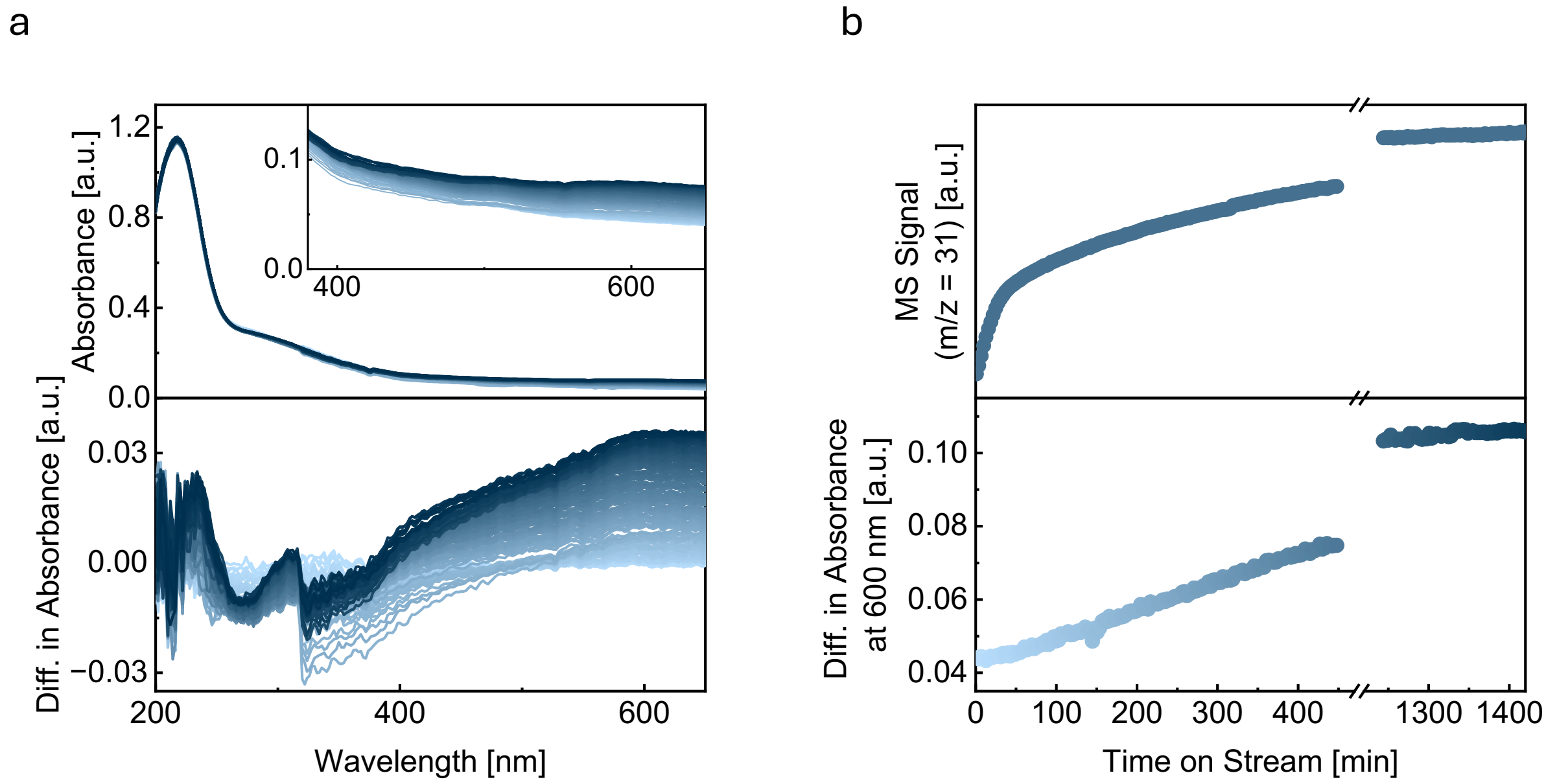

Figure S46: Operando UV-Vis Spectroscopy of 20ZnHfO$_x$. (a) Operando UV-Vis spectra of 20ZnHfO$_x$ and difference in absorbance with respect to the first UV-Vis spectrum at 20 bar, 320 °C, H_2:CO_2:N_2 = 3:1:1 and 24 L(g_{cat}h)$^{-1}$. (b) Evolution of the methanol signal in the off-gas analyzed by MS and difference in absorbance at a wavelength of 600 nm during the operando UV-Vis spectroscopy experiment.

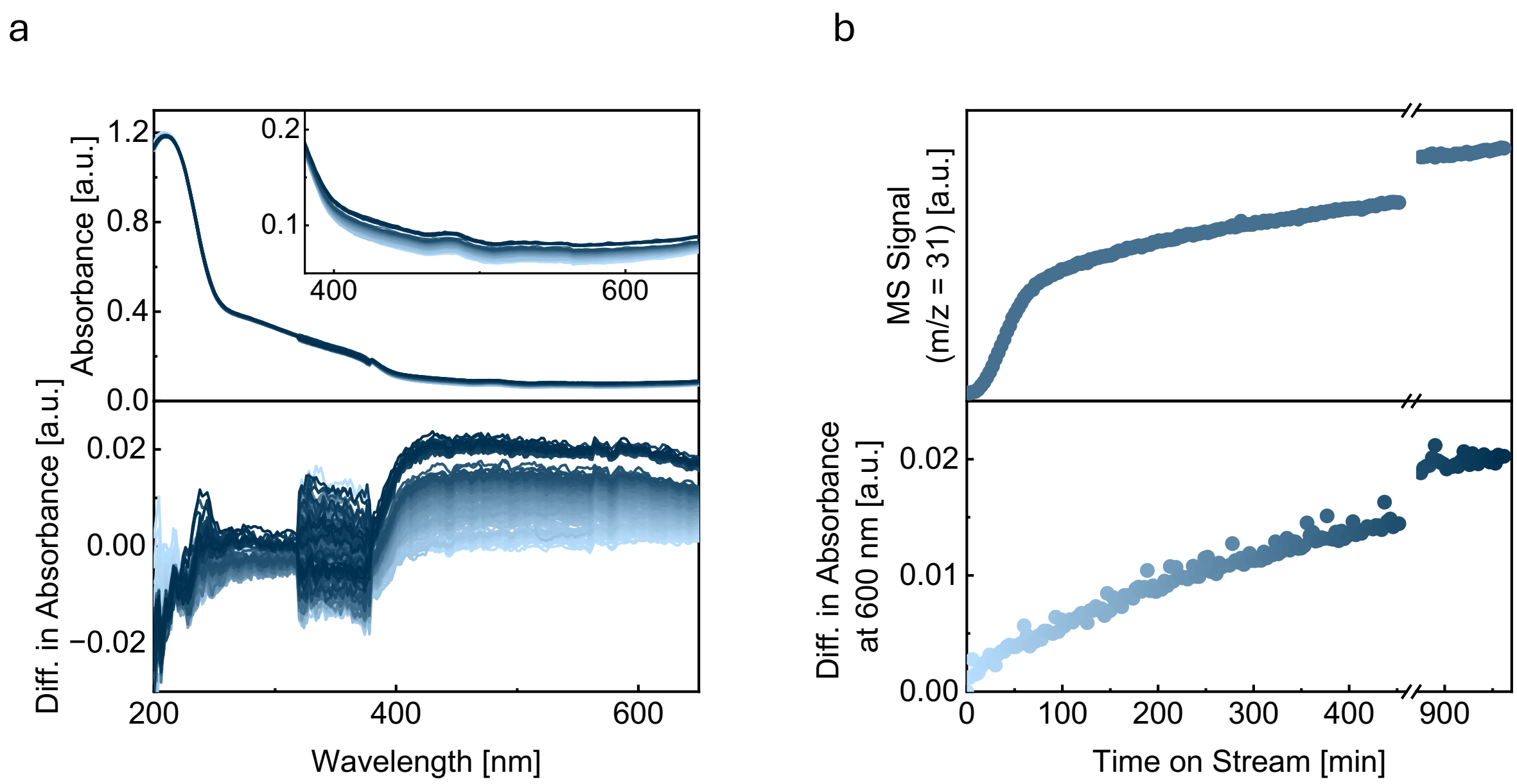

Figure S47: Operando UV-Vis Spectroscopy of 30ZnHfO*x*. (a) Operando UV-Vis spectra of 30ZnHfO*x* and difference in absorbance with respect to the first UV-Vis spectrum at 20 bar, 320 °C, H_2:CO_2:N_2 = 3:1:1 and 24 $L(g_{cat}h)^{-1}$. (b) Evolution of the methanol signal in the off-gas analyzed by MS and difference in absorbance at a wavelength of 600 nm during the operando UV-Vis spectroscopy experiment.

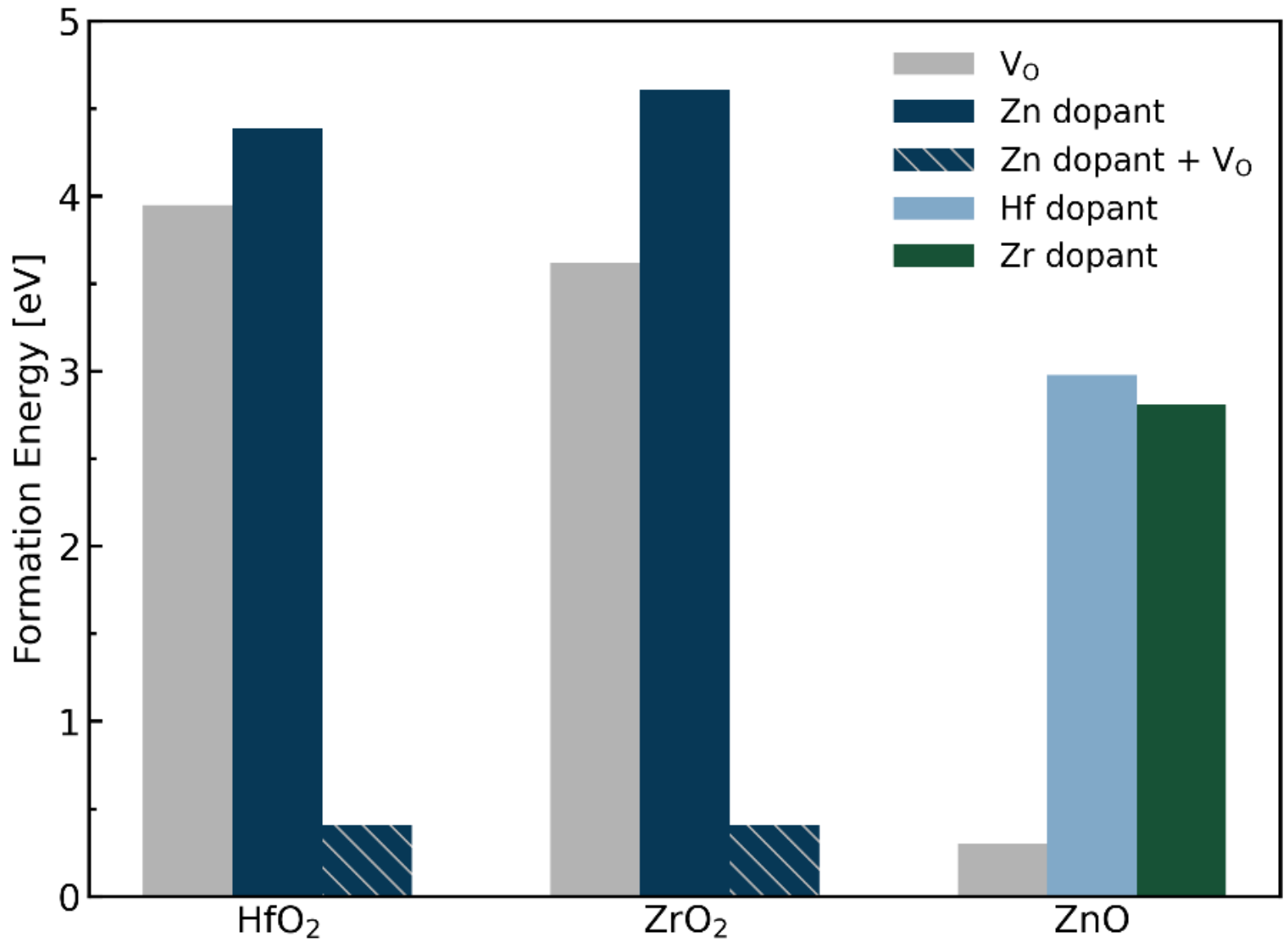

Figure S48: Comparison of the formation energies for creating an oxygen vacancy, a substitutional Zn dopant, and a combined Zn dopant–oxygen vacancy motif in HfO_2 and ZrO_2.Only oxygen vacancy formation and a substitutional dopant are considered in ZnO surfaces. Hf- and Zr-doped ZnO structures were used as approximate models to represent a high Zn-loading doped surface.

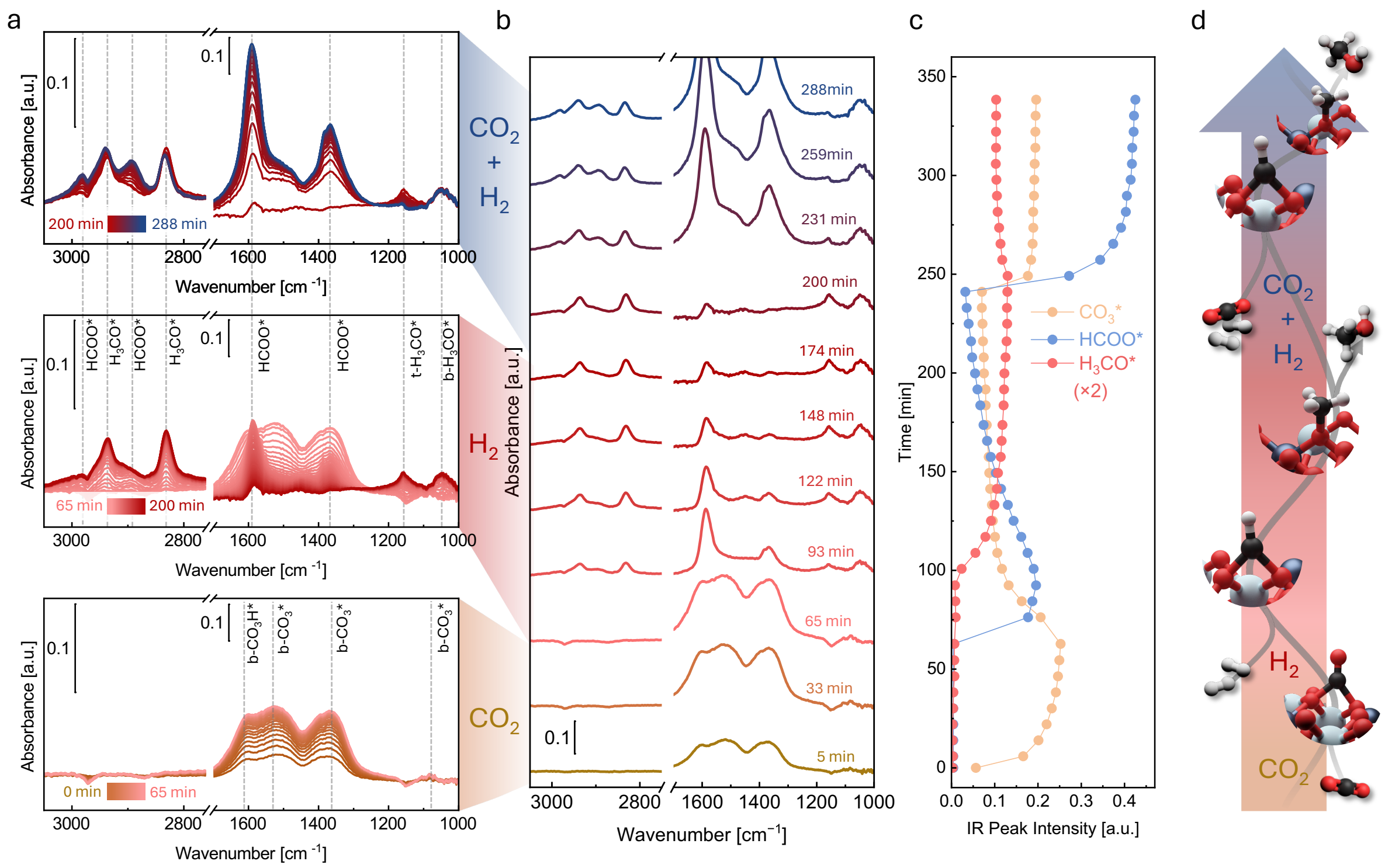

Figure S49: In-situ DRIFTS of 20ZnHfO$_x$. (a) Evolution of the IR spectra in CO_2, H_2, and a mixture of H_2 and CO_2 (ratio 3:1) containing atmospheres at 320 °C and 20 bar total pressure. (b) Selected spectra collected during the operando DRIFTS experiment. (c) Evolution of CO_3* (1532 cm^{-1}), HCOO* (1578 cm^{-1}), and H_3CO* (2827 cm^{-1}) species with time. (d) Illustration of the evolution of the gas phase and the corresponding surface species during the operando DRIFTS experiment.

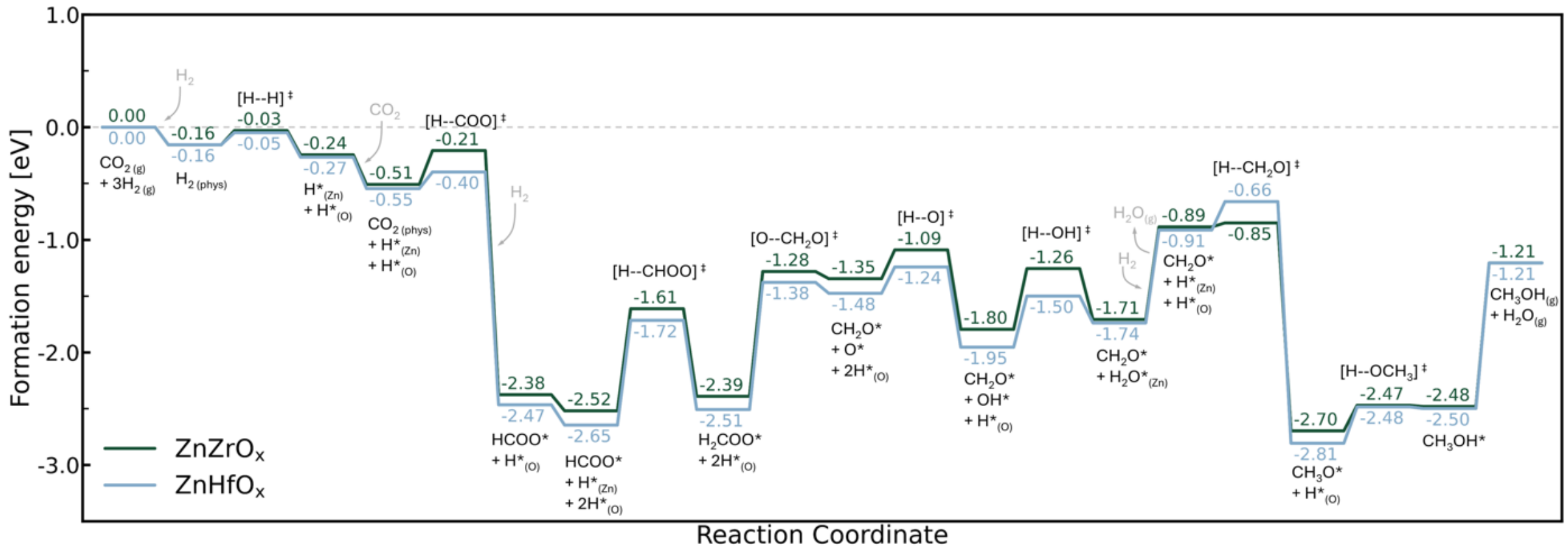

Figure S50: Relative stability of reaction intermediates. Formation energy for selected reaction intermediates on $ZnHfO_x$ and $ZnZrO_x$. The formation energies were calculated with respect to a clean slab and the total energies of gas-phase H_2, H_2O, and CO_2, as described in[30].

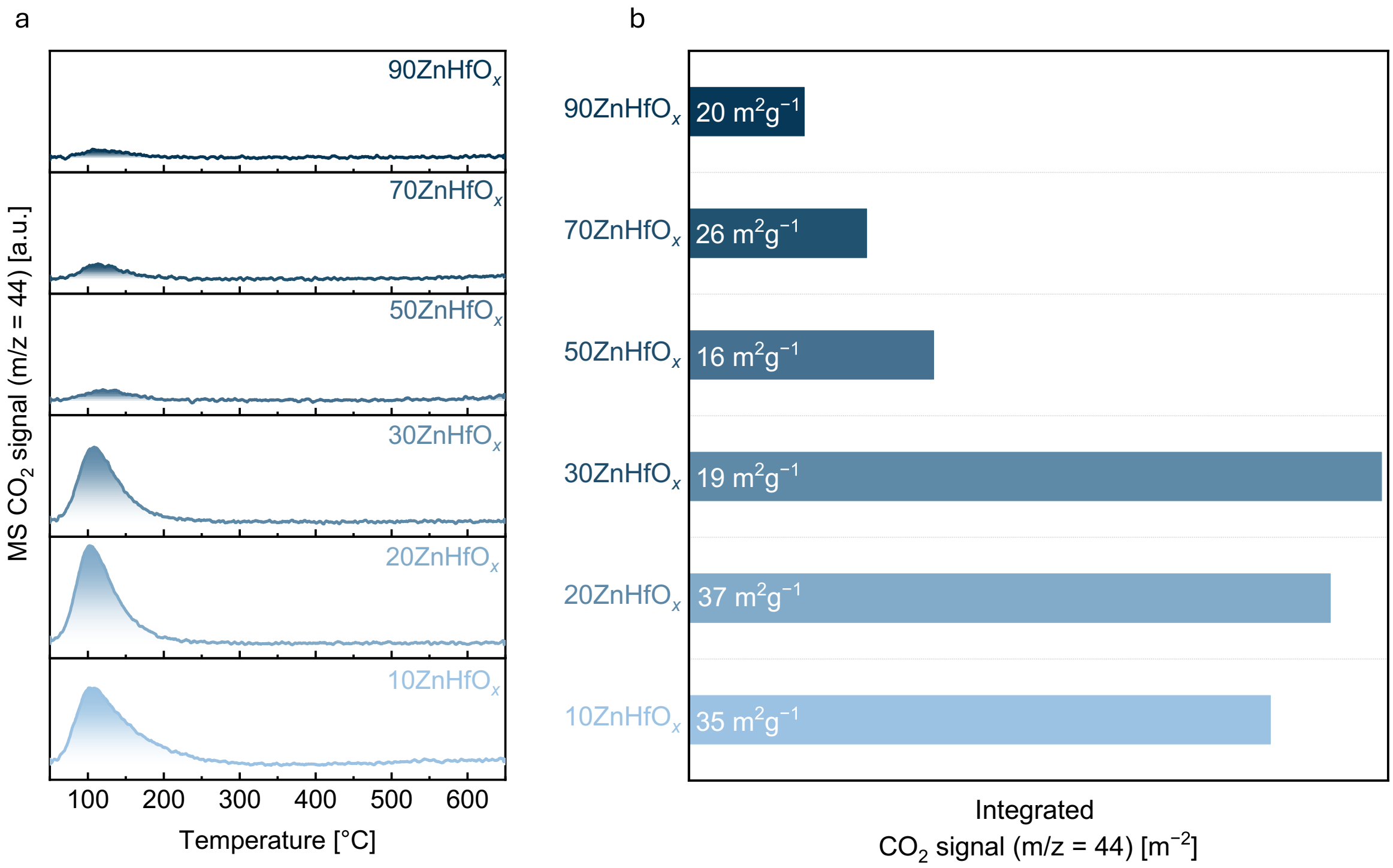

Figure S51: CO_2 TPD of $ZnHfO_x$ with Zn contents ranging from 10 – 90 mol%. (a) CO_2 TPD profiles of $ZnHfO_x$ with Zn contents ranging from 10 – 90 mol%. (b) Integrated CO_2 TPD curves of $ZnHfO_x$ with Zn contents ranging from 10 – 90 mol% normalized by surface area. The surface areas of the individual catalysts are displayed inside the bars.

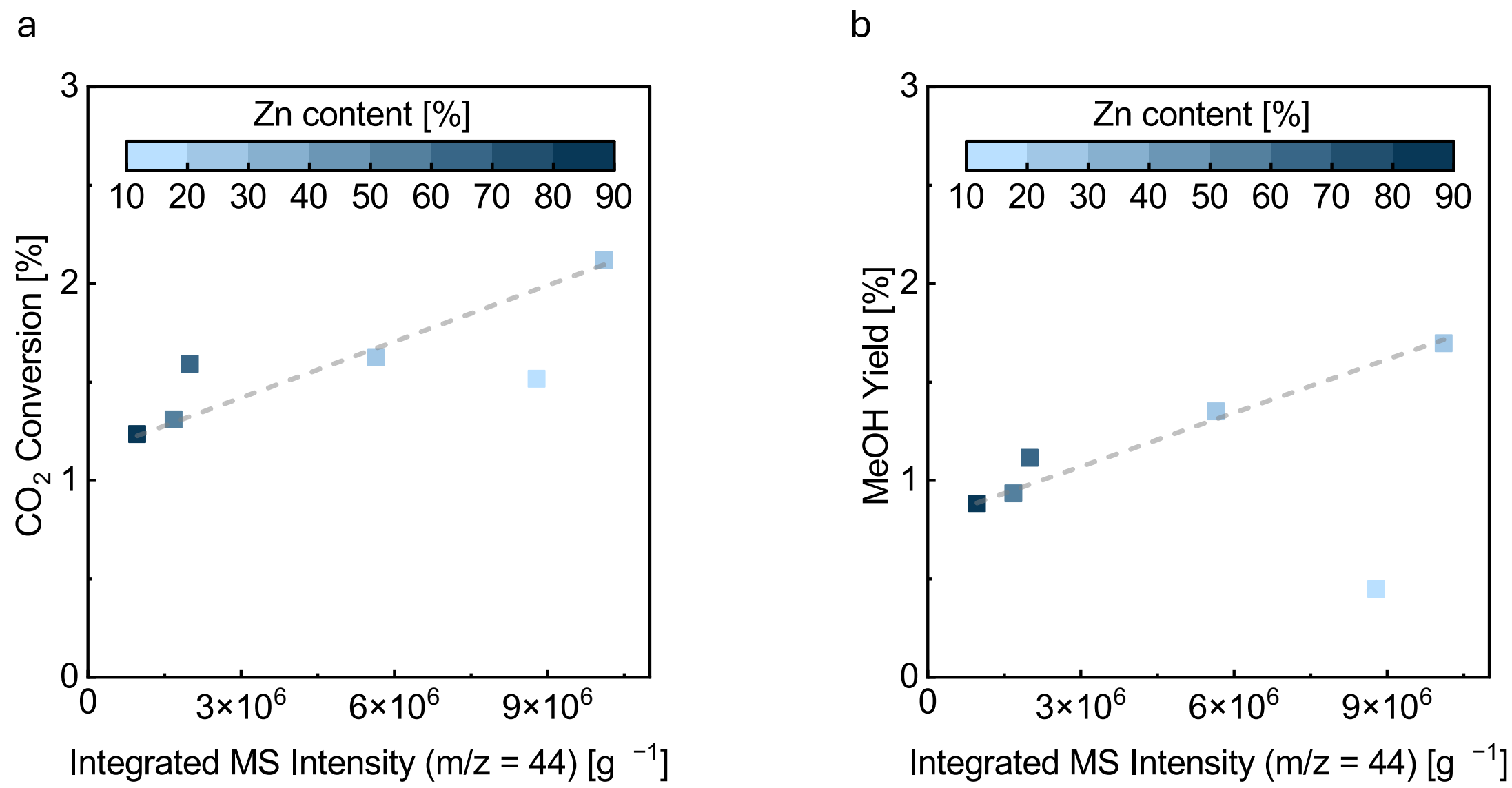

Figure S52: CO_2 TPD results vs catalytic performance of $ZnHfO_x$ with Zn contents ranging from 10 – 90 mol%. (a) CO_2 conversion vs amount of CO_2 desorbed during CO_2 TPD experiments of $ZnHfO_x$ with Zn contents ranging from 10 – 90 mol%. (b) Methanol yield vs amount of CO_2 desorbed during CO_2 TPD experiments of $ZnHfO_x$ with Zn contents ranging from 10 – 90 mol%.

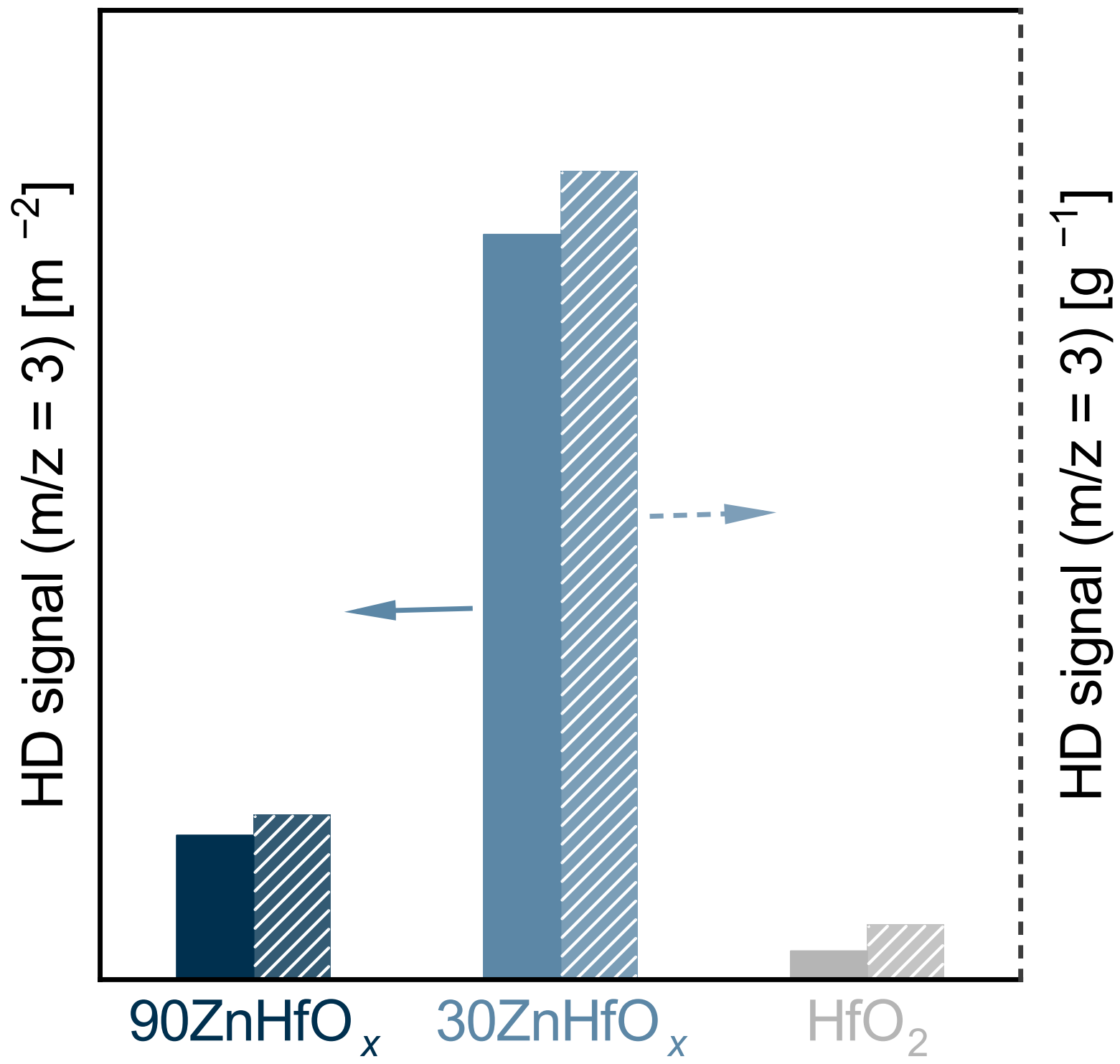

Figure S53: HD exchange experiments of $90ZnHfO_x$, $30ZnHfO_x$, and HfO_2. HD signal intensity normalized by surface area and mass for $90ZnHfO_x$, $30ZnHfO_x$, and HfO_2.

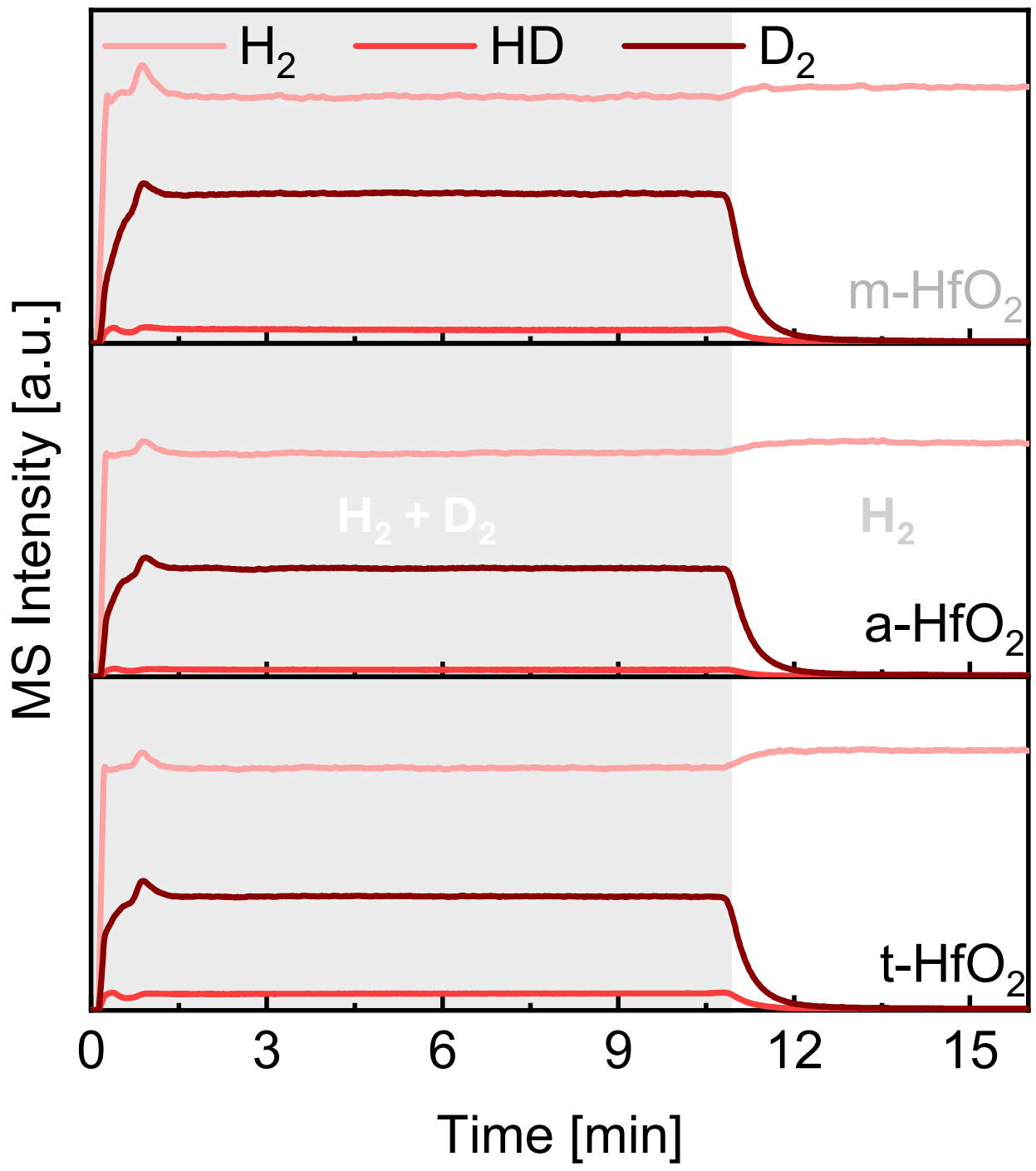

Figure S54: H_2-D_2 exchange experiments of HfO_2 polymorphs. MS signal (m/z = 2 for H_2, m/z = 3 for HD, and m/z = 4 for D_2) of an H_2-D_2 exchange experiment of monoclinic (m-HfO_2), amorphous (a-HfO_2), and tetragonal (t-HfO_2) HfO_2 at 320 °C and a H_2 concentration of 10 vol%, D_2 concentration of 5 vol% and a total flow rate of 20 mL min^{-1}.

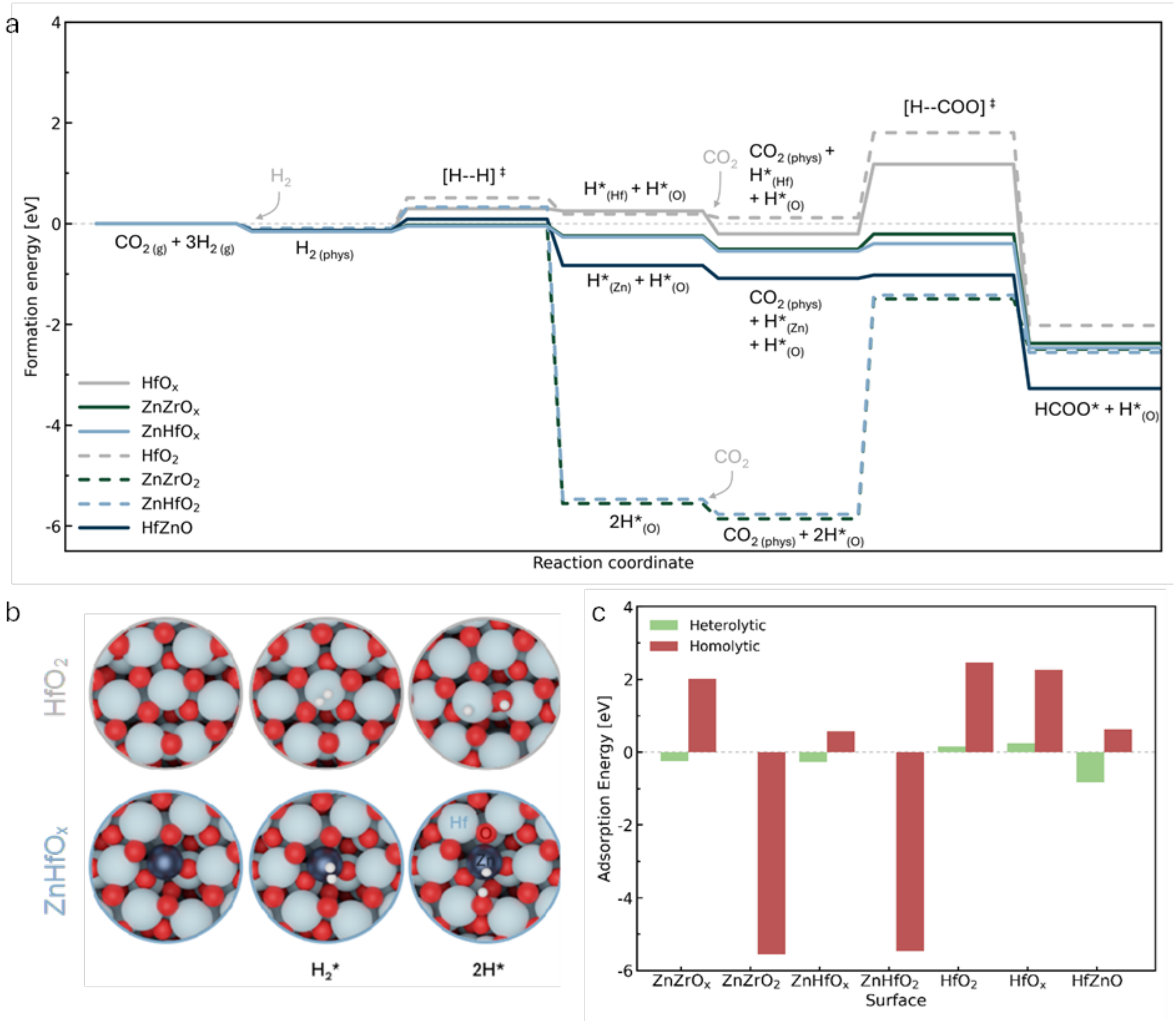

Figure S55: Relative stability and catalytic activity of various HfO_2 surfaces toward hydrogen adsorption and activation, followed by formate formation. ZrO_2 surfaces are included as reference systems. (a) Potential energy diagram for the heterolytic dissociation of H_2 and the subsequent formate formation on different HfO_2- and ZrO_2-based surfaces. Formation energies were calculated relative to the clean slab and the total energies of gas-phase H_2, H_2O, and CO_2, following the methodology described in[30]. (b) Representative surface snapshots of the optimized adsorption configurations of molecular hydrogen (H_2*) and dissociatively adsorbed hydrogen (2H*). Hf atoms are shown in light blue, Zn in dark blue, O in red, and H in white. (c) Relative adsorption energies of dissociatively adsorbed hydrogen species (2H*) resulting from different hydrogen activation mechanisms, namely heterolytic dissociation, which yields hydride and proton pairs (H^-/H^+), and homolytic dissociation. The bars corresponding to heterolytic dissociation of H_2 on $ZnHfO_2$ and $ZnZrO_2$ are absent because the heterolytically dissociated states cannot be retained during geometry relaxation.

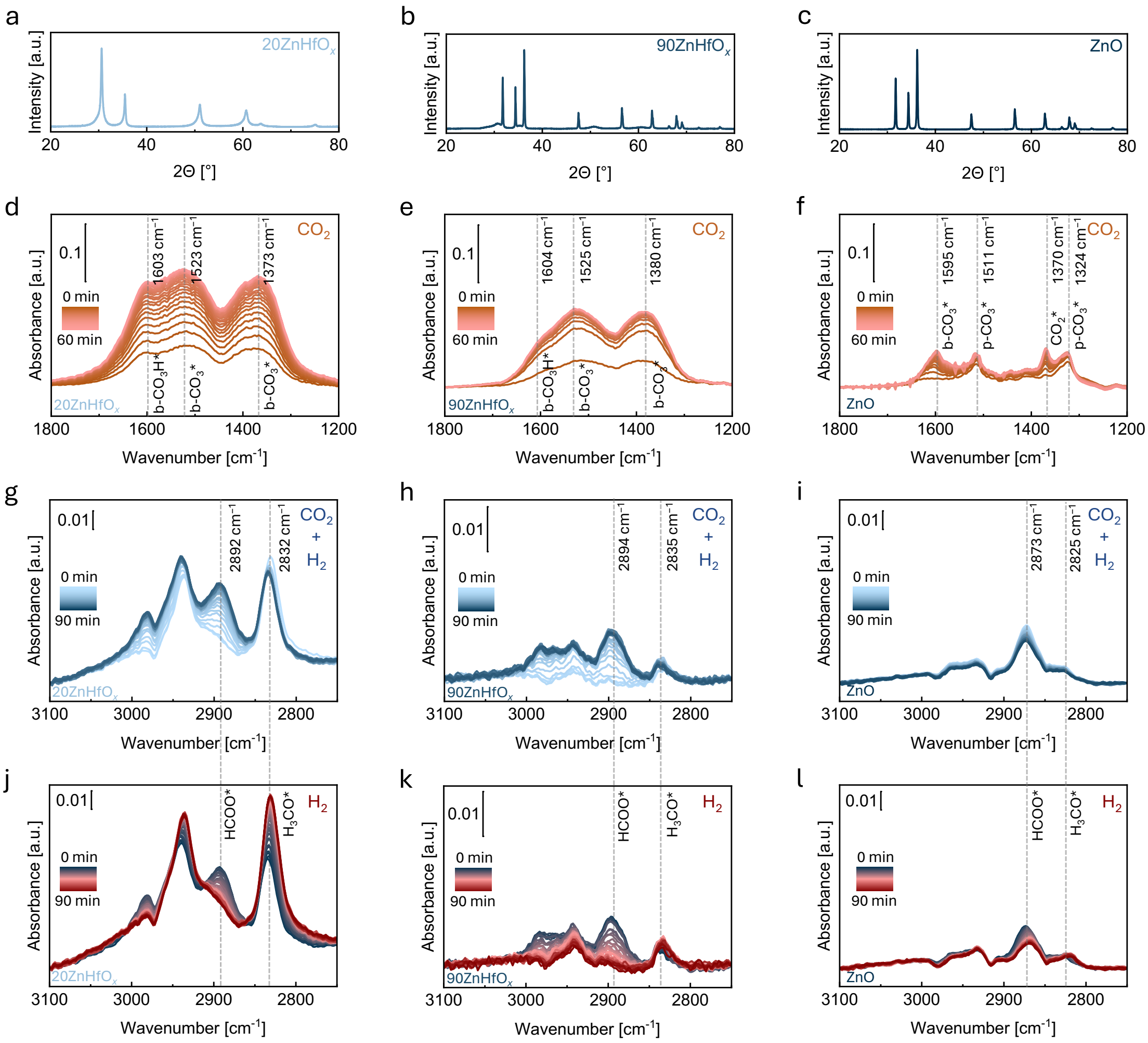

Figure S56: Comparison of reaction intermediates on 20ZnHfO$_x$, 90ZnHfO$_x$, and ZnO. XRD patterns of (a) 20ZnHfO$_x$, (b) 90ZnHfO$_x$, and (c) ZnO. Evolution of the IR spectra of (d) 20ZnHfO$_x$, (e) 90ZnHfO$_x$, and (f) ZnO in CO_2:N_2 = 1:4 at 320 °C and 20 bar total pressure. Evolution of the IR spectra of (g) 20ZnHfO$_x$, (h) 90ZnHfO$_x$, and (i) ZnO in reaction conditions (H_2:CO_2:N_2 = 3:1:1) at 320 °C and 20 bar total pressure. Evolution of the IR spectra of (j) 20ZnHfO$_x$, (k) 90ZnHfO$_x$, and (l) ZnO after switching from reaction conditions to H_2:N_2 = 4:1 at 320 °C and 20 bar total pressure.

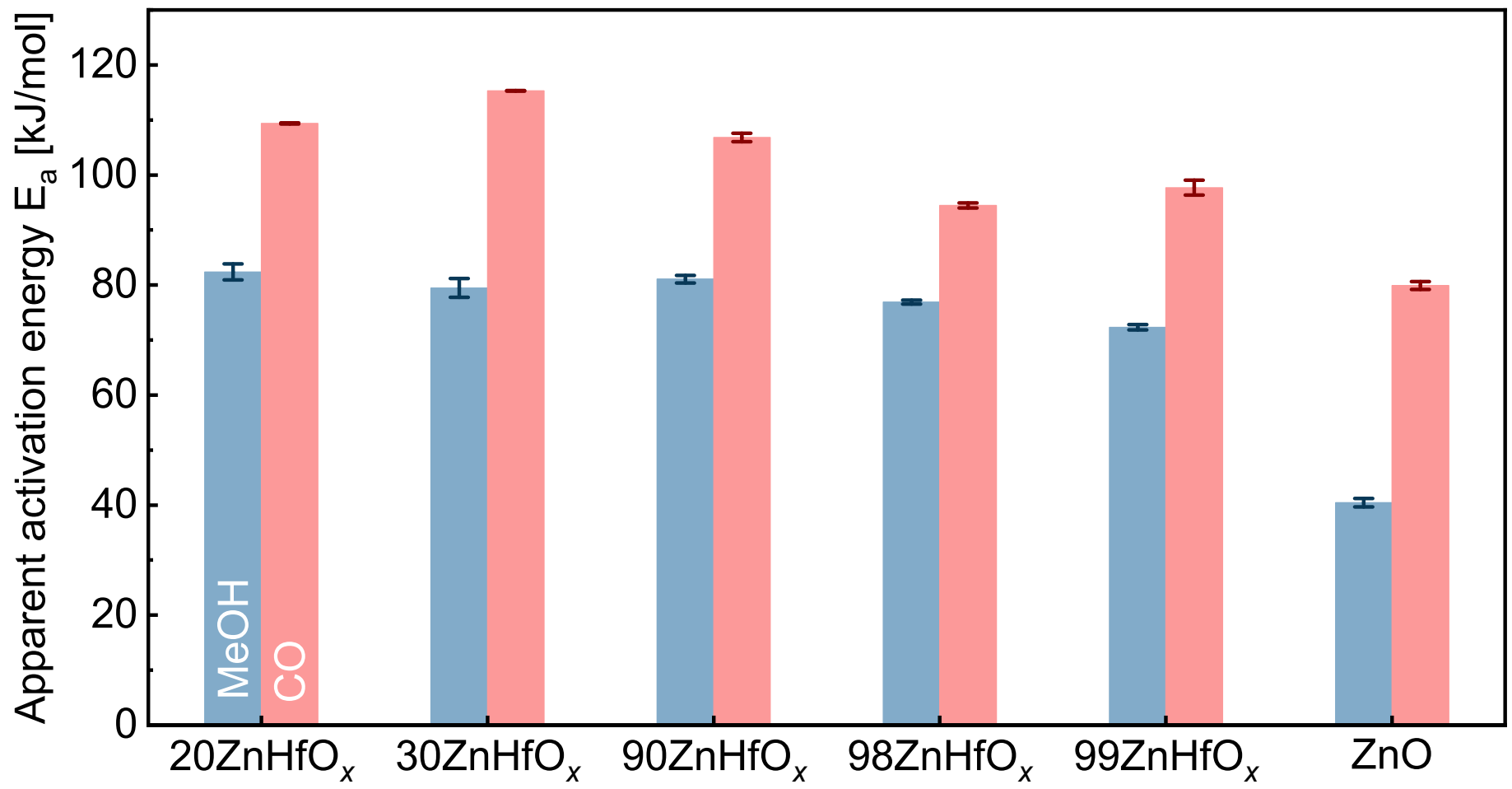

Figure S57: Comparison of the apparent activation energies. Comparison of apparent activation energies for methanol and CO formation of 20ZnHfO*x*, 30ZnHfO*x*, 90ZnHfO*x*, 98ZnHfO*x*, 99ZnHfO*x*, and ZnO. Error bars indicate the error of determining the apparent activation energies from an Arrhenius plot.

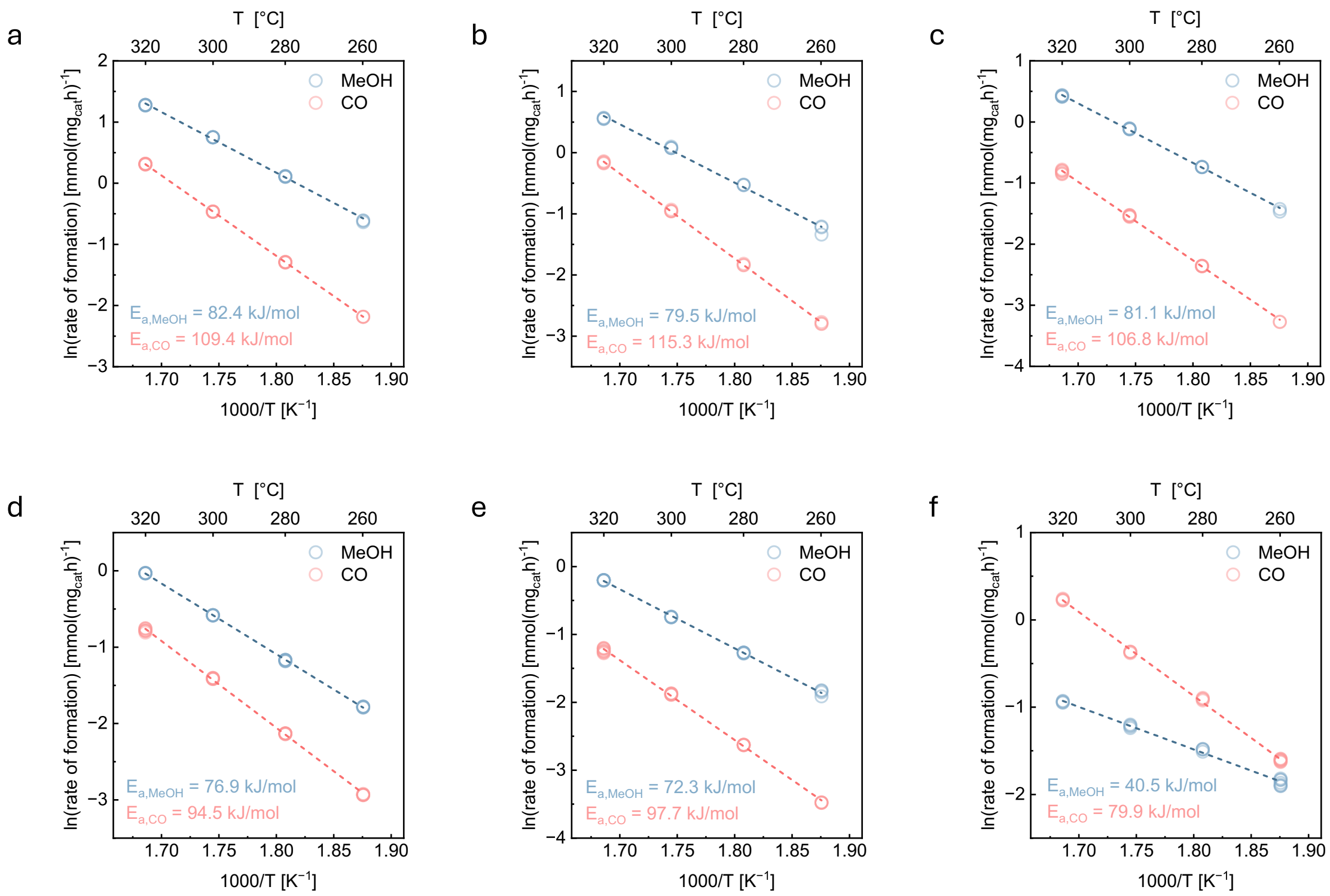

Figure S58: Arrhenius plots of $ZnHfO_x$ catalysts and ZnO. Arrhenius plots of (a) $20ZnHfO_x$, (b) $30ZnHfO_x$, (c) $90ZnHfO_x$, (d) $98ZnHfO_x$, (e) $99ZnHfO_x$, and (f) ZnO for CO and methanol formation.

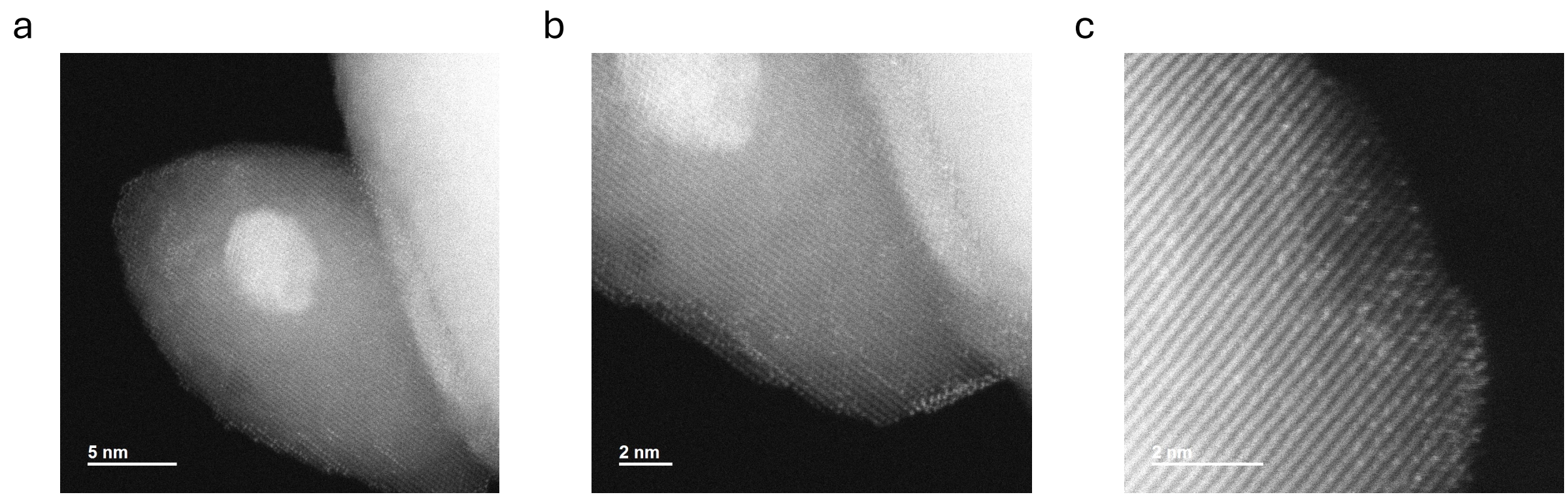

Figure S59: HAADF-STEM images of 98ZnHfO_x after reaction. (a) – (c) High resolution HAADF-STEM images of 98ZnHfO_x after catalytic testing at 20 bar and 320 °C in CO_2 and H_2. The bulk ZnO sufaces of 98ZnHfO_x after reaction reveal Hf/HfO_x single atoms/clusters demonstrating their high stability under harsh CO_2 hydrogenation conditions.

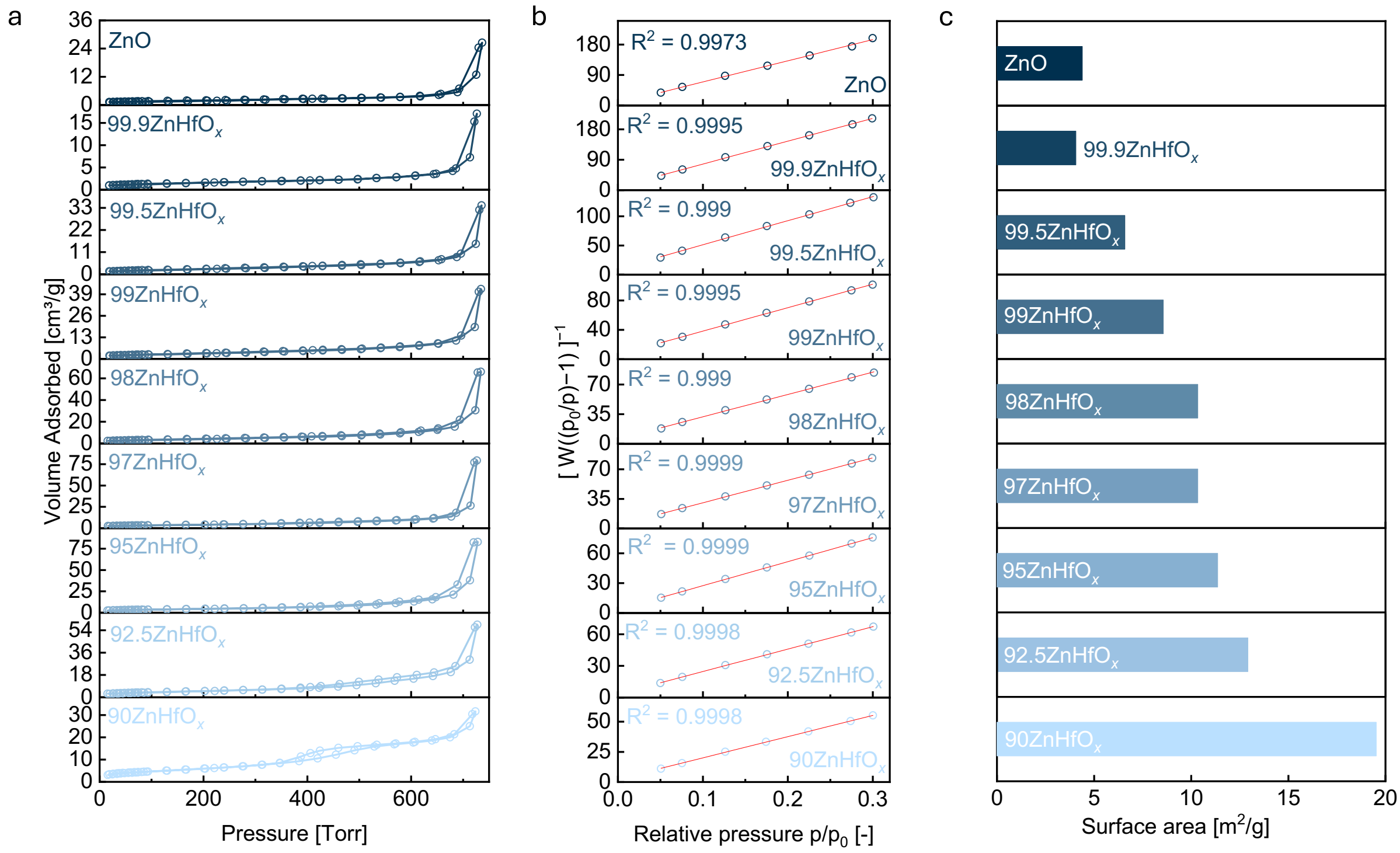

Figure S60: N_2 physisorption of $ZnHfO_x$ catalysts at very high Zn contents (90 – 99.9 mol%). (a) N_2 physisorption isotherms, (b) BET fits, and (c) calculated surface areas for $ZnHfO_x$ catalysts with Zn contents ranging from 90 – 99.9 mol%.

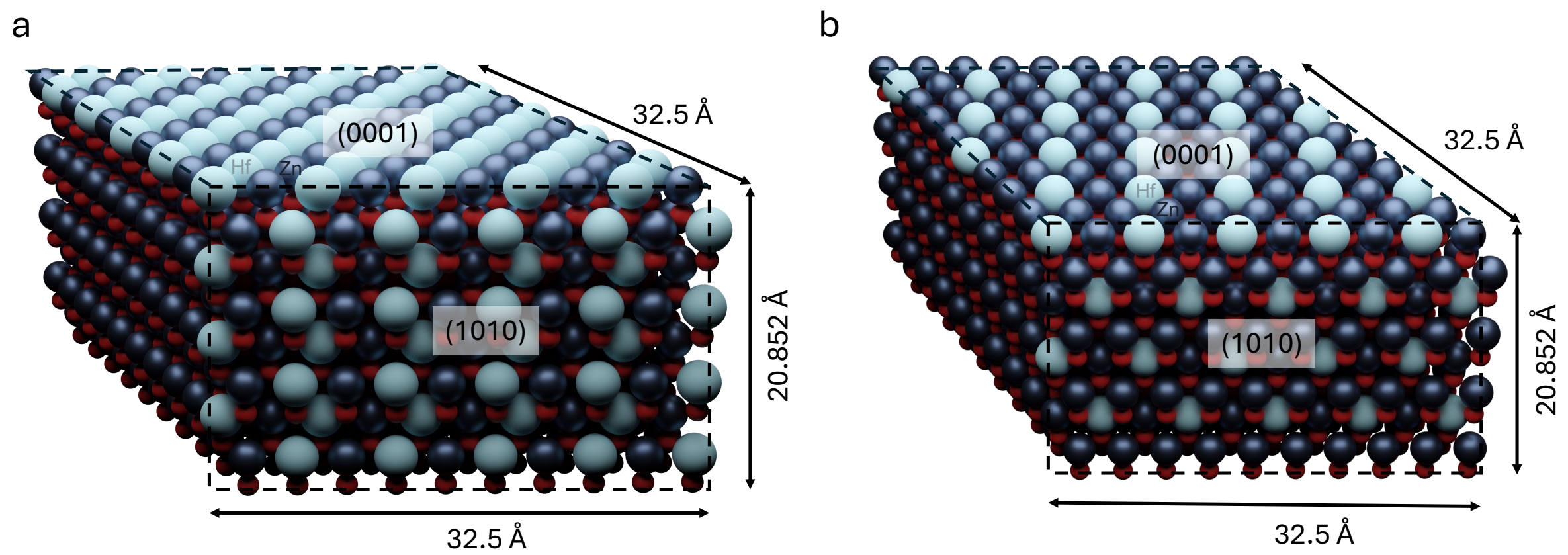

Figure S61: Models of $ZnHfO_x$ catalyst at very high Zn contents (> 99 mol%). 10 × 10 × 4 models of hexagonal ZnO with **a** 50 % and **b** 25 % of Zn atoms (dark blue) at the (0001) and (1010) facets replaced by Hf (atoms). Oxygen atoms are colored red.

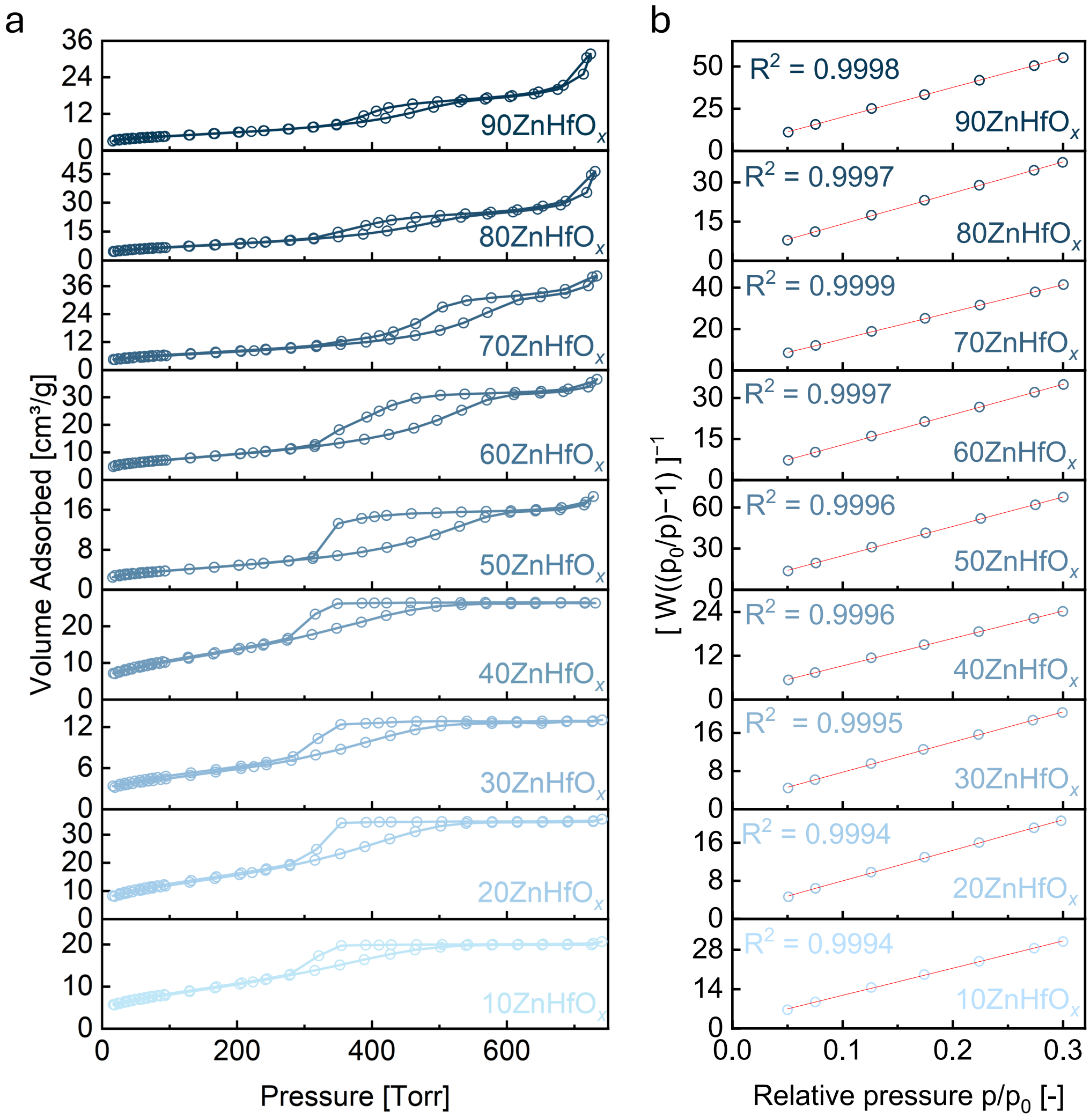

Figure S62: N_2 physisorption of as-prepared $ZnHfO_x$ catalysts with a Zn content ranging from 10 – 90 mol%. (a) N_2 physisorption isotherms of $ZnHfO_x$ catalysts. (b) BET fits to obtain the specific surface areas of the $ZnHfO_x$ samples.

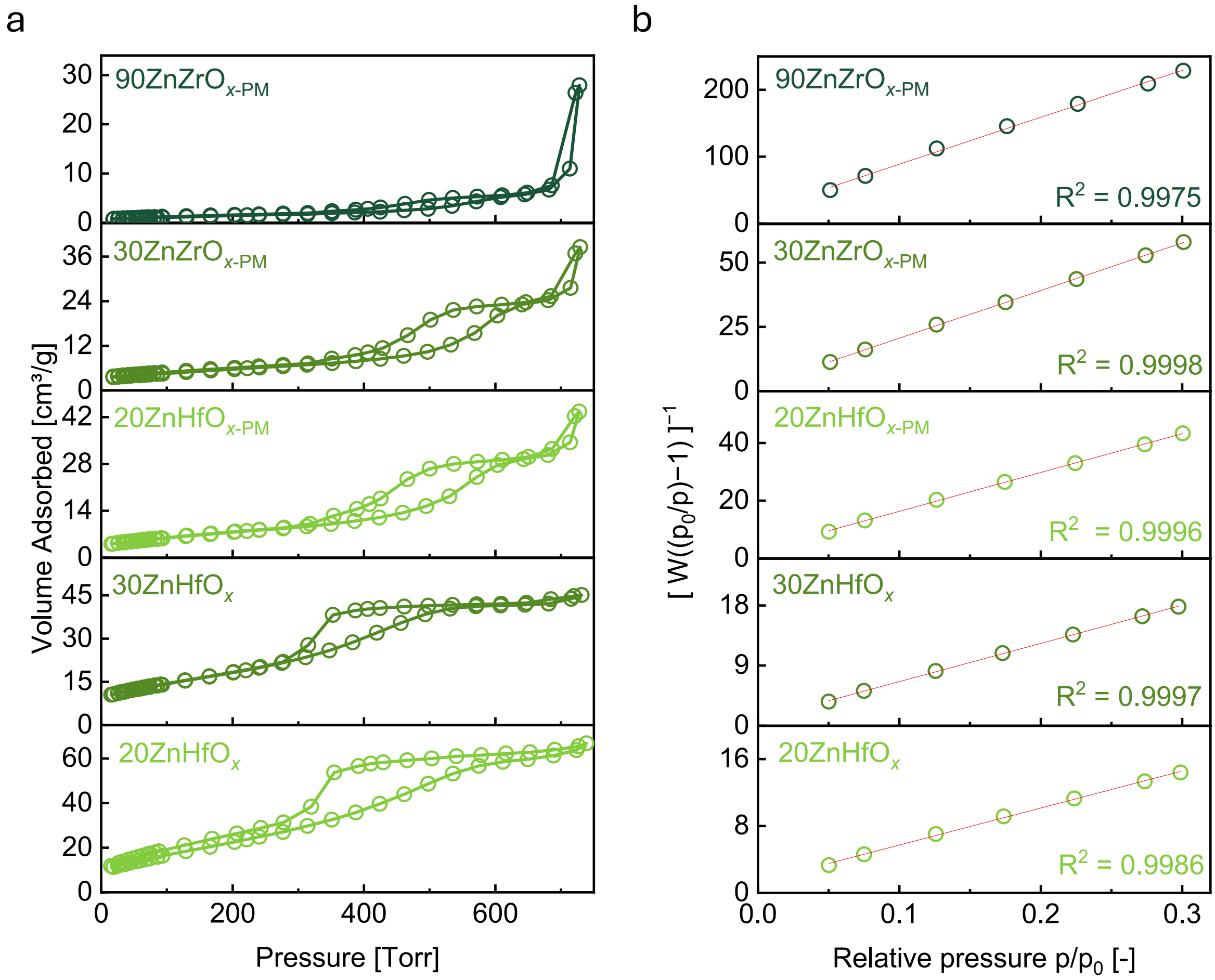

Figure S63: N_2 physisorption of as-prepared $ZnZrO_x$ and $ZnZrO_{x\text{-PM}}$ catalysts with Zn contents of 20, 30, and 90 mol%. (a) N_2 physisorption isotherms of $ZnZrO_x$ and $ZnZrO_{x\text{-PM}}$ catalysts. (b) BET fits to obtain the specific surface area of $ZnZrO_x$ and $ZnZrO_{x\text{-PM}}$ catalysts.

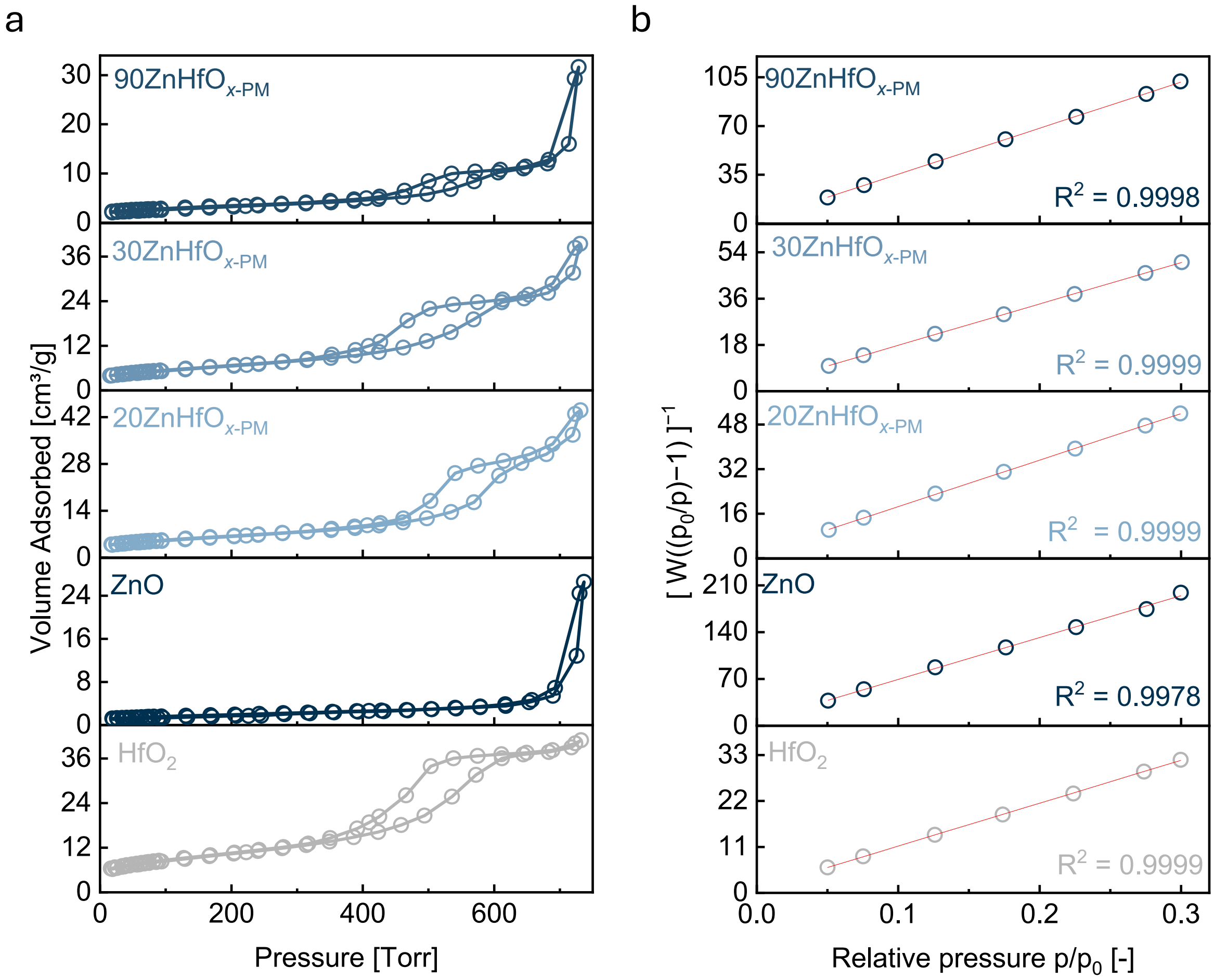

Figure S64: N_2 physisorption of as-prepared ZnHfO$_{x\text{-IWI}}$ and ZnHfO$_{x\text{-PM}}$ catalysts with Zn contents of 20, 30, and 90 mol%. (a) N_2 physisorption isotherms of ZnHfO$_{x\text{-PM}}$ catalysts as well as ZnO and HfO_2. (b) BET fits to obtain the specific surface area of ZnHfO$_{x\text{-PM}}$ catalysts catalysts as well as ZnO and HfO_2.

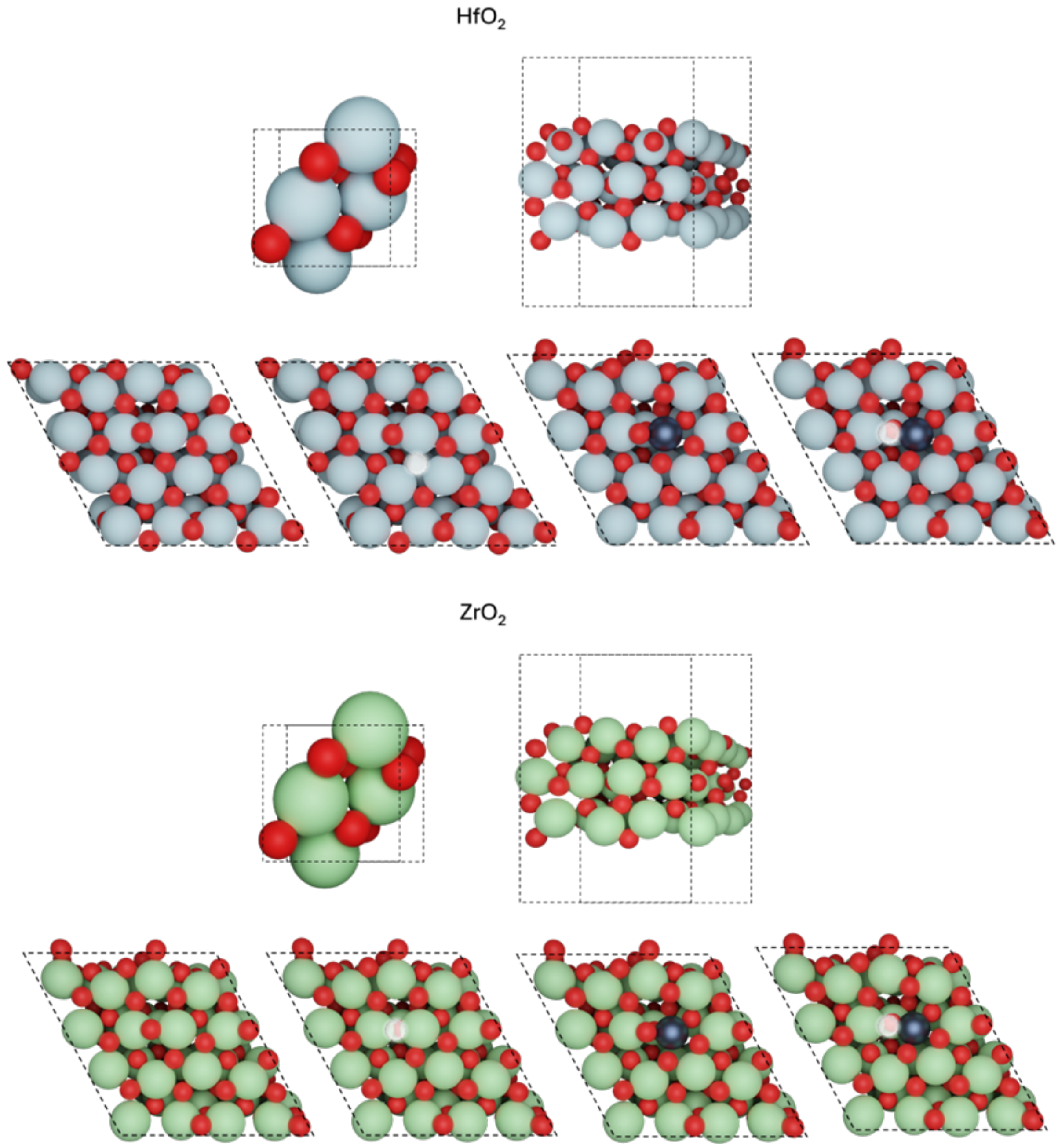

Figure S65: DFT slab models for Zn-doped and pristine ZrO_2 and HfO_2. Top images on each structures shows the bulk and side view of the slab models, while the image at the bottom illustrate the top view of the slab models. Hf atoms are shown in light blue, Zr in green, Zn in dark blue, O in red, and the oxygen vacancy is indicated by a transparent white sphere.

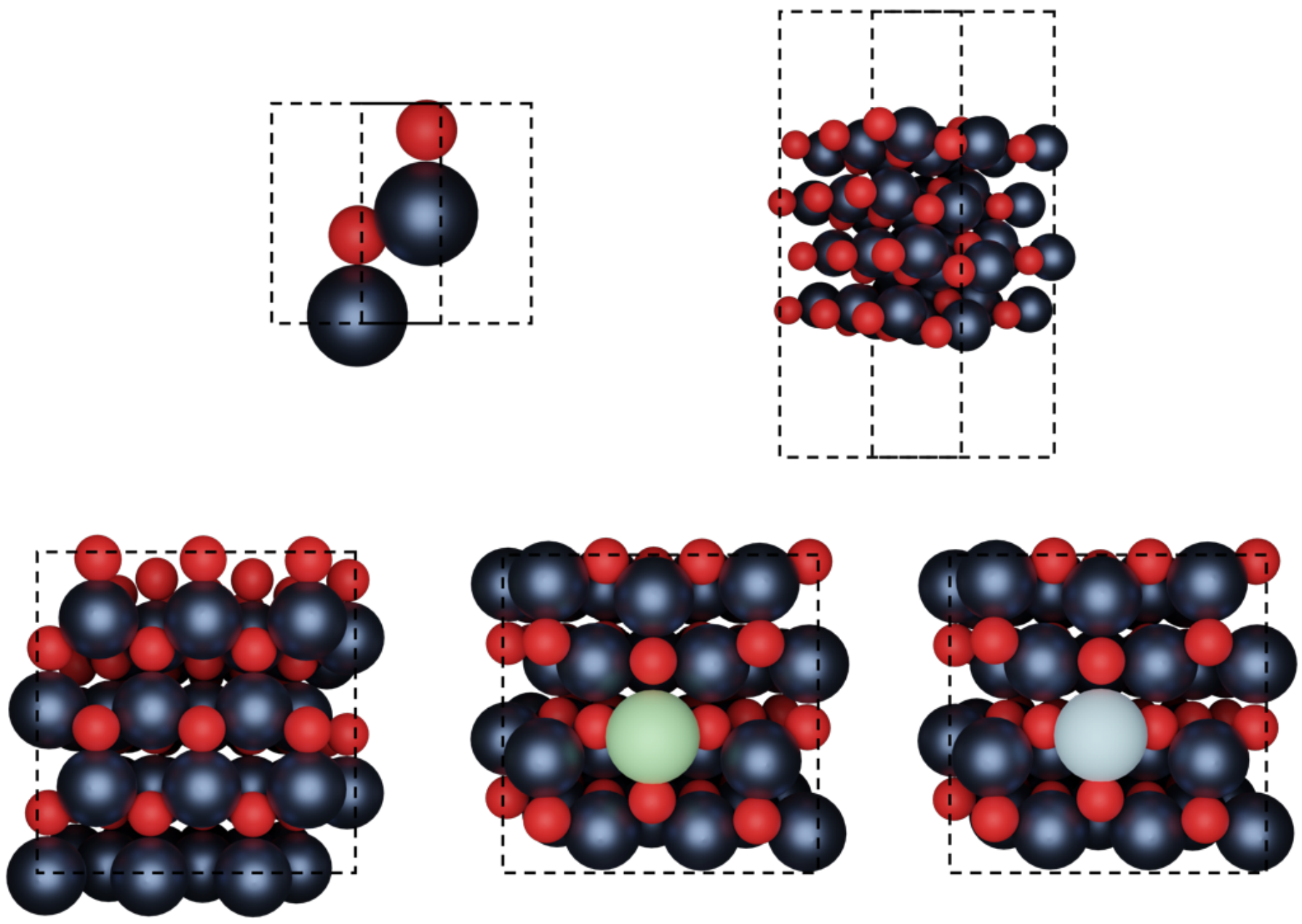

Figure S66: DFT slab models for pristine, Zr-doped, and Hf-doped ZnO. The top image shows bulk ZnO and the side view of the slab models. The bottom image illustrates the top view of the slab models. Hf atoms are shown in light blue, Zr in green, Zn in dark blue, and O in red.

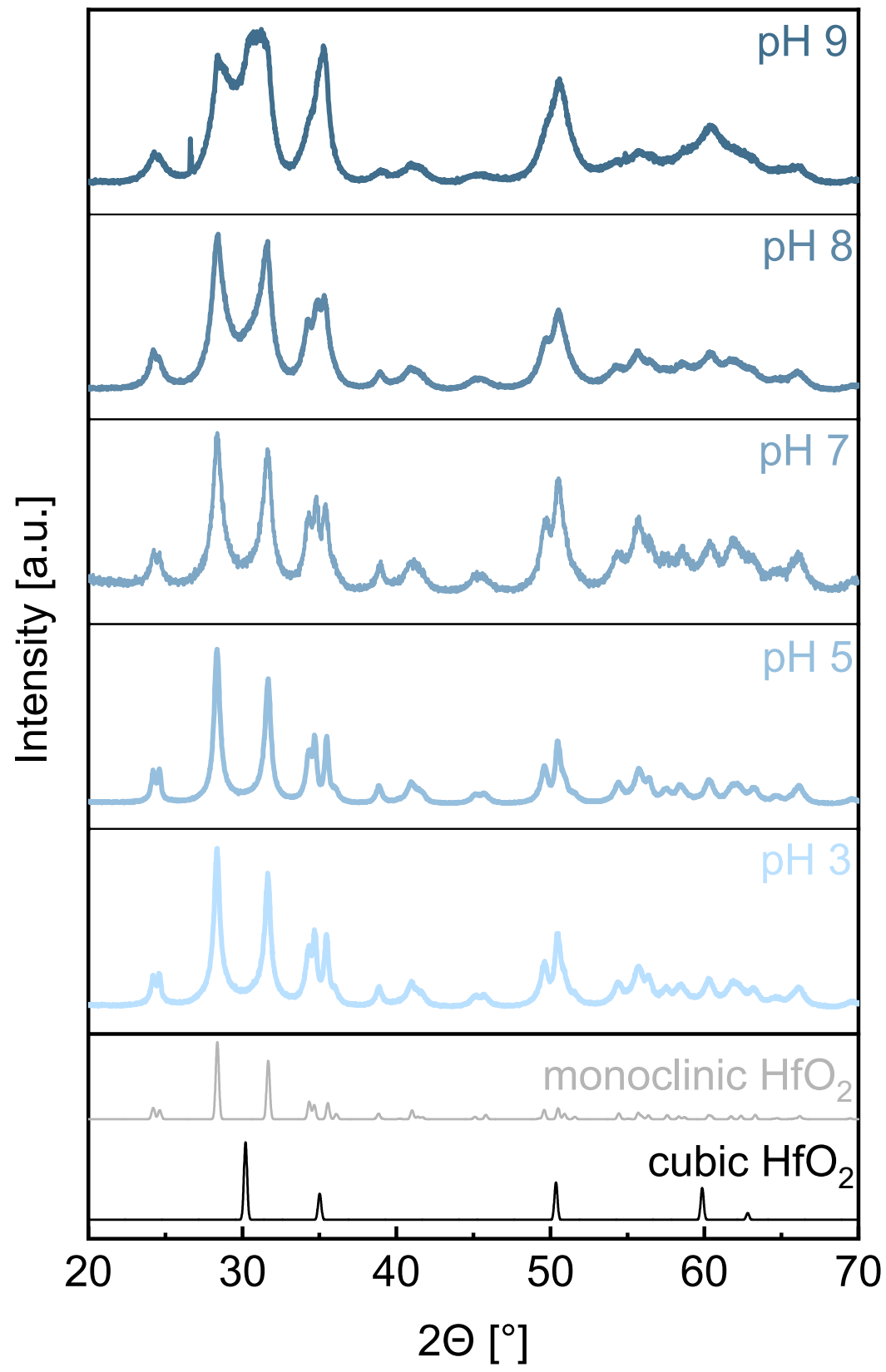

Figure S67: XRD patterns of as-prepared 10ZnHfO$_x$ precipitated at various pH levels. XRD patterns of 10ZnHfO$_x$ precipitated at various pH levels show a contribution of cubic/tetragonal reflections for pH levels > 7.

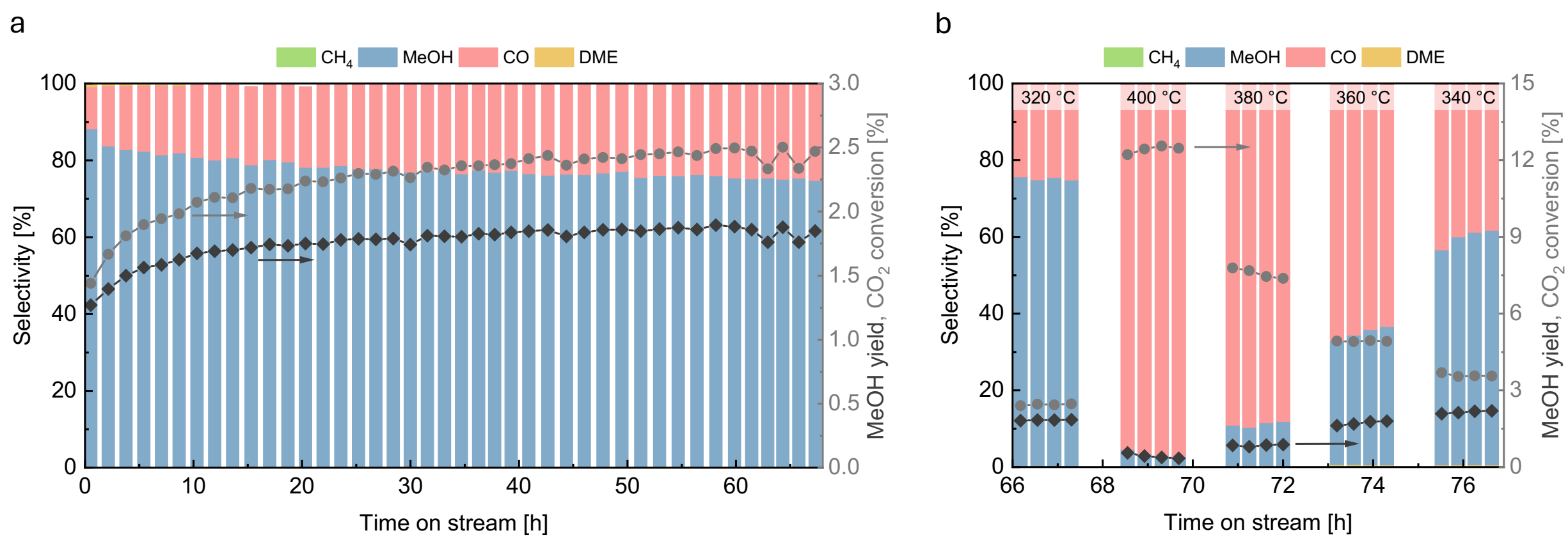

Figure S68: Long-term performance assessment of 20ZnHfO$_x$. (a) Long-term performance assessment of 20ZnHfO$_x$ at 20 bar, 320 °C, H_2:CO_2:N_2 = 3:1:1, and 24 L(g_{cat}h)$^{-1}$ for 68 h and (b) a consecutive temperature sweep from 400 to 340 °C at 20 bar, H_2:CO_2:N_2 = 3:1:1.

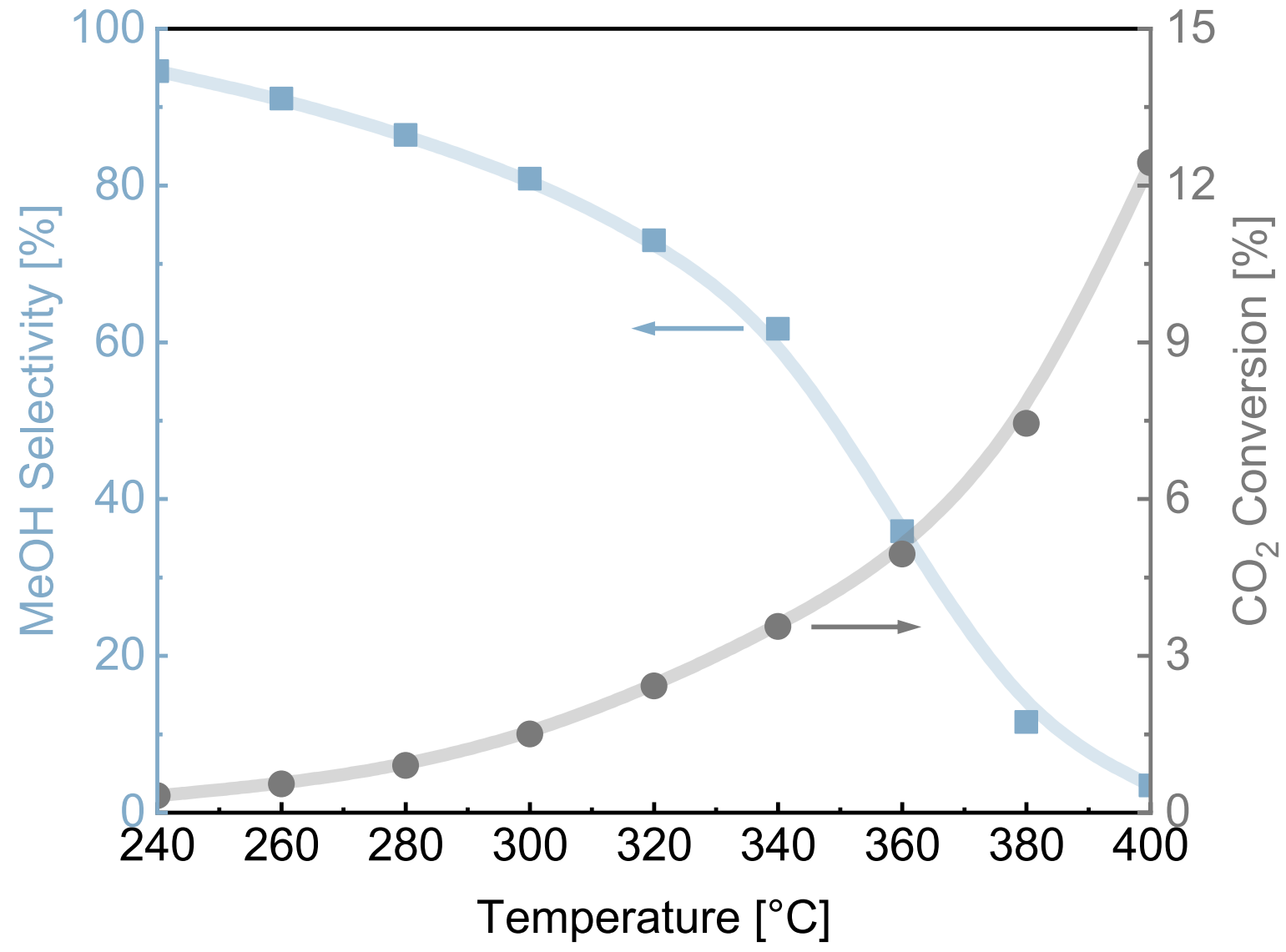

Figure S69: Temperature sweep of 20ZnHfO*x*. Temperature dependent evaluation of the catalytic performance of a fully activated 20ZnHfO*x* catalyst in a temperature range from 240 – 400 °C at 20 bar, H_2:CO_2:N_2 = 3:1:1, and 24 $L(g_{cat}h)^{-1}$.

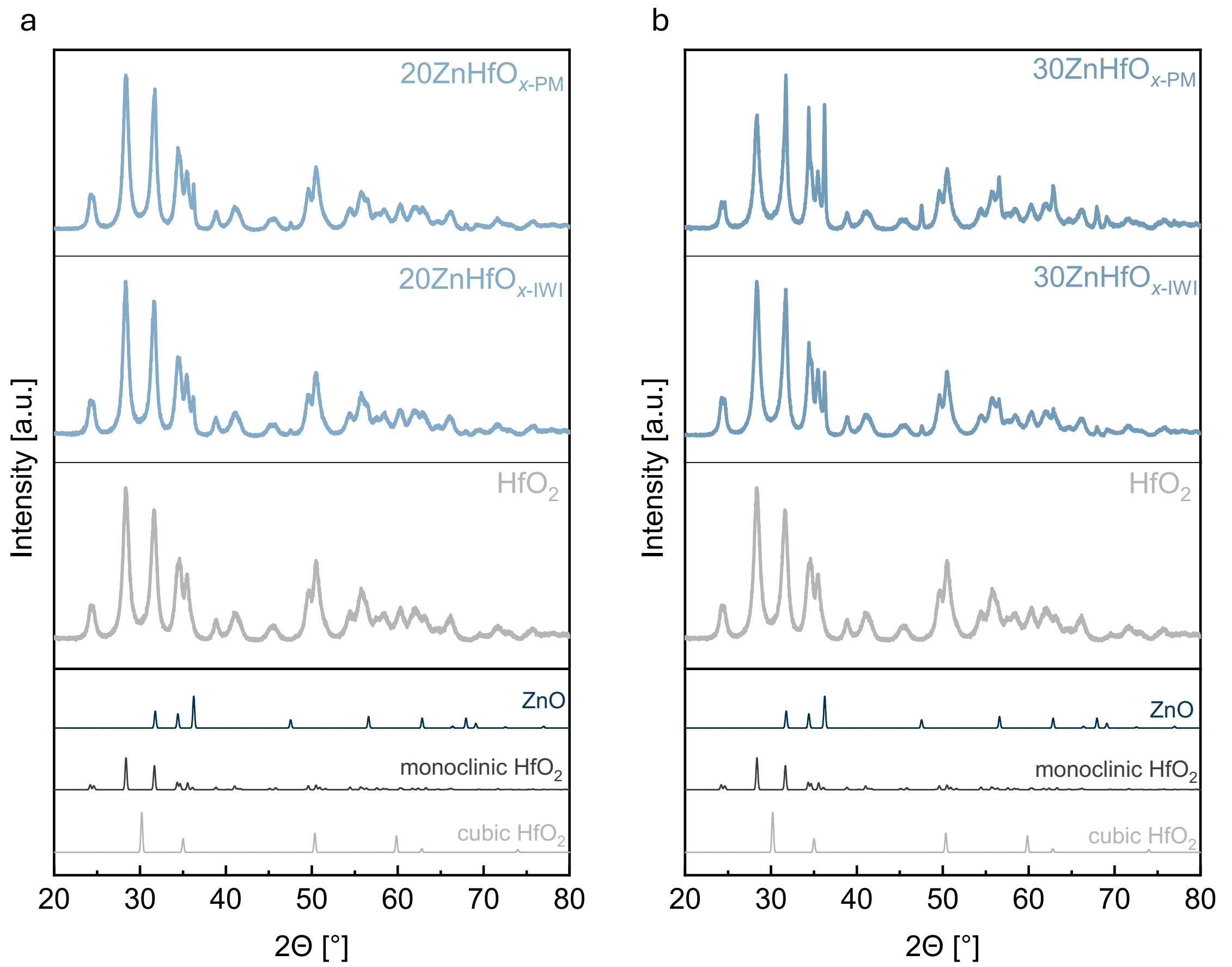

Figure S70: XRD patterns of as-prepared reference $ZnHfO_x$ catalysts. XRD patterns of (a) $20ZnHfO_x$ and (b) $30ZnHfO_x$ synthesized from incipient wetness impregnation (IWI) and physical mixing (PM), as well as the HfO_2 support material.

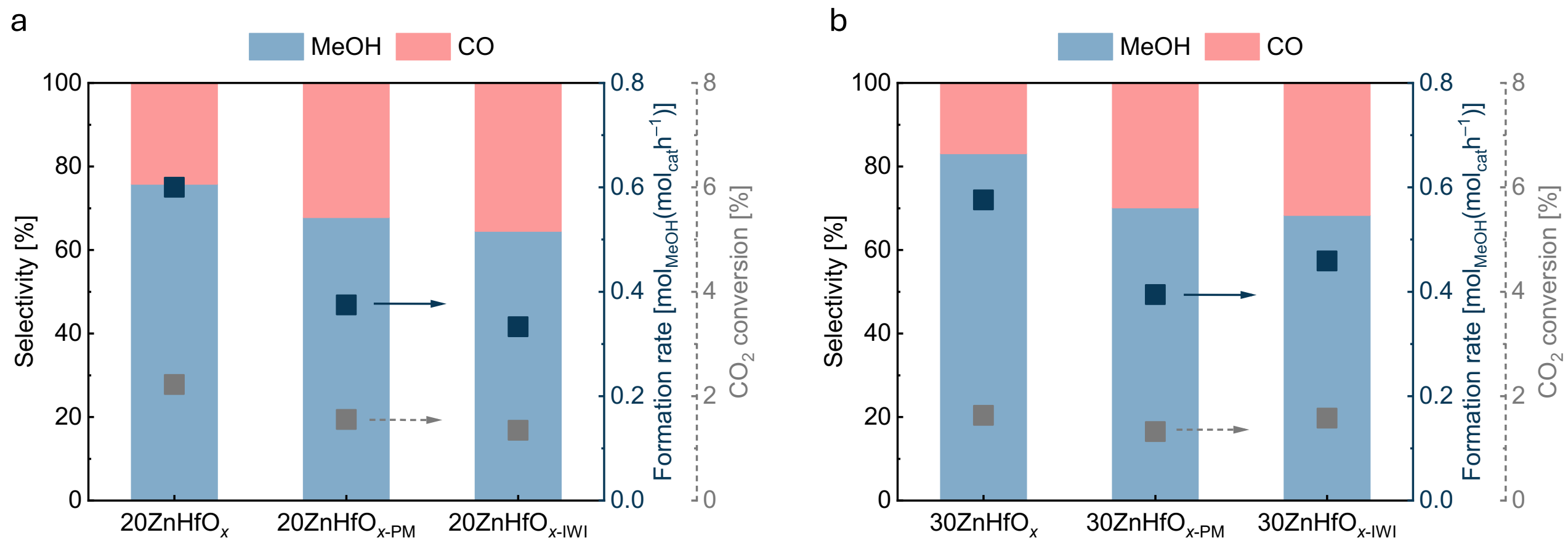

Figure S71: Catalytic performance of reference $ZnHfO_x$ catalysts. The catalytic performance of (a) $20ZnHfO_x$ compared to $20ZnHfO_{x\text{-PM}}$ and $20ZnHfO_{x\text{-IWI}}$ and (b) $30ZnHfO_x$ compared to $30ZnHfO_{x\text{-PM}}$ and $30ZnHfO_{x\text{-IWI}}$ at 20 bar, 320 °C, H_2:CO_2:N_2 = 3:1:1, and 24 $L(g_{cat}h)^{-1}$.

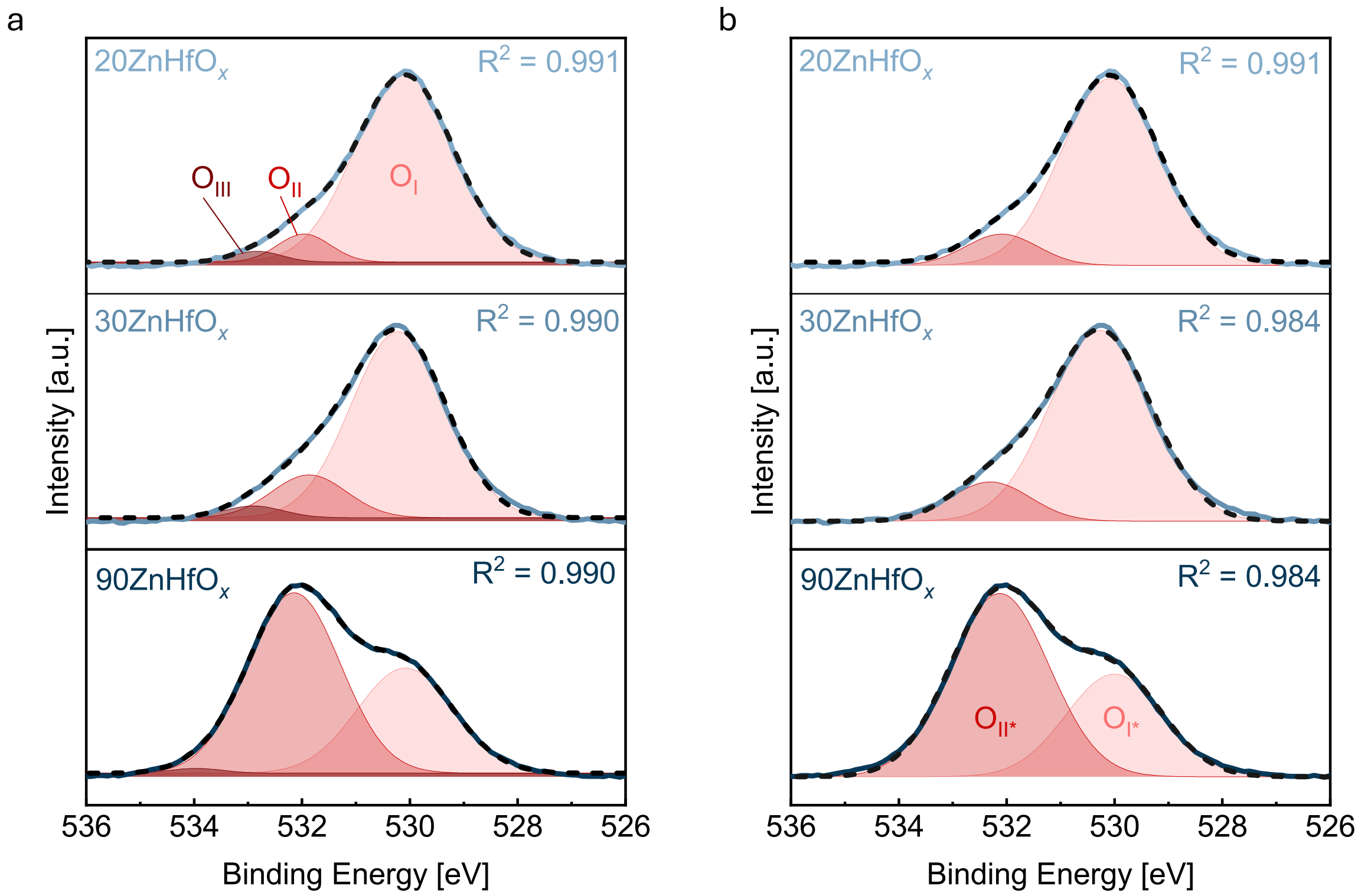

Figure S72: O 1s XPS of ZnHfO$_x$ after reaction. O 1s XPS spectra of 20ZnHfO$_x$, 30ZnHfO$_x$, and 90ZnHfO$_x$ after reaction fitted with (a) three Gaussians and (b) two Gaussians. The goodness of fit is indicated in each frame.

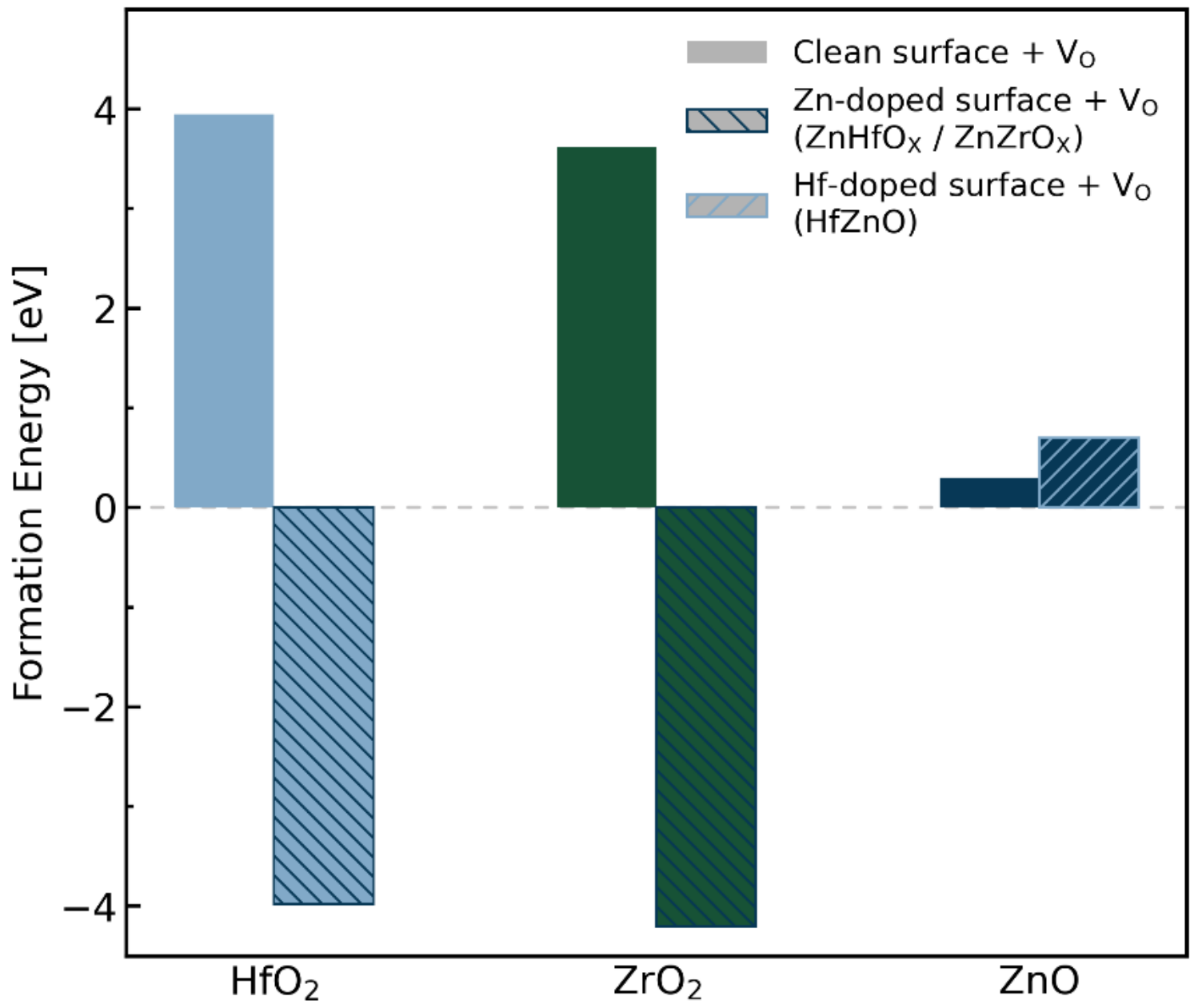

Figure S73: Calculated formation energies of oxygen vacancies on the topmost layer of HfO_2(−111), $ZnHfO_2$(−111), ZrO_2(−111), $ZnZrO_2$(−111), ZnO(1010), and HfZnO(1010). The formation energies were calculated relative to the energies of pristine or doped surfaces without an oxygen vacancy, with the energies of hydrogen and water in the gas phase.

Supplementary Tables

Table S1: Surface areas and pore volumes of $ZnHfO_x$ catalysts and reference materials. N_2 physisorption isotherms were used to determine the surface area with the BET method and the pore volume using the BJH method. CP – co-precipitation, IWI – incipient wetness impregnation, PM – physical mixture, P – precipitation.

Sample	Synthesis route	Calcination temperature [°C]	Surface Area [m^2g^{-1}]	Pore Volume [cm^3g^{-1}]
$10ZnHfO_x$	CP	580	35	0.03
$20ZnHfO_x$	CP	580	37	0.05
$30ZnHfO_x$	CP	580	19	0.02
$40ZnHfO_x$	CP	580	45	0.03
$50ZnHfO_x$	CP	580	16	0.04
$60ZnHfO_x$	CP	580	31	0.07
$70ZnHfO_x$	CP	580	26	0.07
$80ZnHfO_x$	CP	580	29	0.07
$90ZnHfO_x$	CP	580	20	0.05
$92.5ZnHfO_x$	CP	580	16	0.09
$95ZnHfO_x$	CP	580	14	0.13
$97ZnHfO_x$	CP	580	13	0.12
$98ZnHfO_x$	CP	580	13	0.1
$99ZnHfO_x$	CP	580	11	0.06
$99.5ZnHfO_x$	CP	580	8	0.03
$99.9ZnHfO_x$	CP	580	5	0.03
$20ZnZrO_x$	CP	500	70	0.102
$30ZnZrO_x$	CP	500	60	0.069
$20ZnHfO_{x\text{-IWI}}$	IWI	580	18	0.049
$30ZnHfO_{x\text{-IWI}}$	IWI	580	20	0.037
$20ZnHfO_{x\text{-PM}}$	PM	580	21	0.062
$30ZnHfO_{x\text{-PM}}$	PM	580	21	0.055
$90ZnHfO_{x\text{-PM}}$	PM	580	10	0.049

Sample	Synthesis route	Calcination temperature [°C]	Surface Area [m^2g^{-1}]	Pore Volume [cm^3g^{-1}]
$30ZnZrO_{x\text{-}PM}$	PM	580	19	0.064
$90ZnZrO_{x\text{-}PM}$	PM	580	5	0.045
ZnO	P	500	7	0.042
HfO_2	P	580	34	0.060

Table S2: Calculated and experimental values of lattice parameter of bulk HfO_2 and ZrO_2 structures.

Lattice parameter	DFT calculation	Experimental result
HfO_2		
a	5.11 Å	5.11 Å[31]
b	5.18 Å	5.17 Å
c	5.28 Å	5.22 Å
β	99.54°	99.30°
ZrO_2		
a	5.15 Å	5.15 Å[32]
b	5.24 Å	5.21 Å
c	5.32 Å	5.32 Å
β	99.41°	99.23°

Table S3: Adsorption energies of initial intermediates on the ZnO and HfZnO structures for CO_2 hydrogenation via the formate pathway.

Intermediate	Adsorption energy (eV)
ZnO	
H_2	−0.20
2H	−0.37
CO_2	−0.69
HCOO	−2.11
HfZnO	
H_2	−0.13
2H	−0.83
CO_2	−1.08
HCOO	−3.27

Supplementary References

[1] J. Wang, G. Li, Z. Li, C. Tang, Z. Feng, H. An, H. Liu, T. Liu, C. Li, "A highly selective and stable ZnO-ZrO_2 solid solution catalyst for CO_2 hydrogenation to methanol" *Sci. Adv.* **2017**, *3*, e1701290.

[2] P. Makuła, M. Pacia, W. Macyk, "How To Correctly Determine the Band Gap Energy of Modified Semiconductor Photocatalysts Based on UV-Vis Spectra" *J. Phys. Chem. Lett.* **2018**, *9*, 6814–6817.

[3] H. Du, "A nonlinear filtering algorithm for denoising HR(S)TEM micrographs" *Ultramicroscopy* **2015**, *151*, 62–67.

[4] D. Zanders, E. Ciftyurek, E. Subaşl, N. Huster, C. Bock, A. Kostka, D. Rogalla, K. Schierbaum, A. Devi, "PEALD of HfO_2 Thin Films: Precursor Tuning and a New Near-Ambient-Pressure XPS Approach to in Situ Examination of Thin-Film Surfaces Exposed to Reactive Gases" *ACS Appl. Mater. Interf.* **2019**, *11*, 28407–28422.

[5] C. F. Liu, X. G. Tang, X. Bin Guo, Q. X. Liu, Y. P. Jiang, Z. H. Tang, W. H. Li, "Photodiode characteristics of HfO_2 thin films prepared by magnetron sputtering" *Mater. Des.* **2020**, *188*.

[6] T. J. Frankcombe, Y. Liu, "Interpretation of Oxygen 1s X-ray Photoelectron Spectroscopy of ZnO" *Chem. Mater.* **2023**, *35*, 5468–5474.

[7] G. Kresse, J. Hafner, "Ab. initio molecular dynamics for liquid metals" *Phys. Rev. B* **1993**, *47*, 558–561.

[8] J. P. Perdew, K. Burke, M. Ernzerhof, "Generalized Gradient Approximation Made Simple" *Phys. Rev. Lett.* **1996**, *77*, 3865–3868.

[9] S. Grimme, J. Antony, S. Ehrlich, H. Krieg, "A consistent and accurate ab initio parametrization of density functional dispersion correction (DFT-D) for the 94 elements H-Pu" *J. Chem. Phys.* **2010**, *132*, 154104.

[10] S. Grimme, S. Ehrlich, L. Goerigk, "Effect of the damping function in dispersion corrected density functional theory" *J. Comput. Chem.* **2011**, *32*, 1456–1465.

[11] P. E. Blochl, "Projector augmented-wave method" *Phys. Rev. B* **1994**, *50*, 17953–17979.

[12] G. Kresse, D. Joubert, "From ultrasoft pseudopotentials to the projector augmented-wave method" *Phys. Rev. B* **1999**, *59*, 1758–1775.

[13] J. A. Garrido Torres, P. C. Jennings, M. H. Hansen, J. R. Boes, T. Bligaard, "Low-Scaling Algorithm for Nudged Elastic Band Calculations Using a Surrogate Machine Learning Model" *Phys. Rev. Lett.* **2019**, *122*, 156001.

[14] J. Wang, H. P. Li, R. Stevens, "Review Hafnia and hafnia-toughened ceramics" *J. Mater. Sci.* **1992**, *27*, 5397–5430.

[15] A. B. Mukhopadhyay, J. F. Sanz, C. B. Musgrave, "First-principles calculations of structural and electronic properties of monoclinic hafnia surfaces" *Phys. Rev. B, Condens. Matter* **2006**, *73*, 115330.

[16] C. Ricca, A. Ringuedé, M. Cassir, C. Adamo, F. Labat, "A comprehensive DFT investigation of bulk and low-index surfaces of ZrO_2 polymorphs" *J. Comput. Chem.* **2015**, *36*, 9–21.

[17] C. Wöll, "The chemistry and physics of zinc oxide surfaces" *Prog. Surf. Sci.* **2007**, *82*, 55–120.

[18] K. Taek Jung, A. T. Bell, "The effects of synthesis and pretreatment conditions on the bulk structure and surface properties of zirconia" *J. Mol. Catal. A Chem.* **2000**, *163*, 27–42.

[19] K. Pokrovski, K. T. Jung, A. T. Bell, “Investigation of CO and CO_2 adsorption on tetragonal and monoclinic zirconia” *Langmuir* **2001**, *17*, 4297–4303.

[20] X. Mao, Y. Zhang, Y. Xu, Y. Zhou, K. Zhuang, K. Shen, S. Ding, “The lattice oxygen determines the methanol selectivity in CO_2 hydrogenation over $ZnZrO_x$ catalysts” *Catal. Sci. Technol.* **2023**.

[21] Q. Ren, K. Yang, F. Liu, M. Yao, J. Ma, S. Geng, J. Cao, “Role of the structure and morphology of zirconia in ZnO/ZrO_2 catalyst for CO_2 hydrogenation to methanol” *Mol. Catal.* **2023**, *547*, 113280.

[22] M. T. Nikolajsen, J.-C. Grivel, A. Gaur, L. P. Hansen, L. Baumgarten, N. C. Schjødt, U. V. Mentzel, J.-D. Grunwaldt, J. Sehested, J. M. Christensen, M. Høj, “Surface ZnO on zirconia is highly active for high temperature methanol synthesis” *J. Catal.* **2024**, *431*, 115389.

[23] H. Idriss, **2021**, Elsevier B.V. preprint.

[24] C. D. Easton, D. J. Morgan, “Critical examination of the use of x-ray photoelectron spectroscopy (XPS) O 1s to characterize oxygen vacancies in catalytic materials and beyond” *J. Vac. Sci. Technol. A* **2025**, *43*.

[25] K. Lee, M. P. Dickieson, M. Jung, Y. Yang, N. Yan, “Structure Sensitivity of $ZnZrO_x$ Catalysts in CO_2 Hydrogenation to Methanol: Significance of Surface Oxygen Content and Synthesis Strategy” *ACS Catal.* **2024**, *14*, 3074–3089.

[26] L. Lin, G. Wang, F. Zhao, “CO_2 Hydrogenation to Methanol on ZnO/ZrO_2 Catalysts: Effects of Zirconia Phase” *ChemistrySelect* **2021**, *6*, 2119–2125.

[27] J. Saussey, C. Bovet, J.-C. Lavalley, “Infrared Study of CO_2 Adsorption on ZnO” *J. Chem. SOC., Faraday Trans. I* **1982**, *78*, 1457–1463.

[28] M. Buchholz, P. G. Weidler, F. Bebensee, A. Nefedov, C. Wöll, “Carbon dioxide adsorption on a ZnO(1010) substrate studied by infrared reflection absorption spectroscopy” *Phys. Chem. Chem. Phys.* **2014**, *16*, 1672–1678.

[29] S. Wang, L. Song, Z. Qu, “$Cu/ZnAl_2O_4$ catalysts prepared by ammonia evaporation method: Improving methanol selectivity in CO_2 hydrogenation via regulation of metal-support interaction” *Chem. Eng. J.* **2023**, *469*.

[30] P. Lozano-Reis, H. Prats, R. Sayós, F. Illas, “Limitations of free energy diagrams to predict the catalytic activity: The reverse water gas shift reaction catalyzed by Ni/TiC” *J. Catal.* **2023**, *425*, 203–211.

[31] A. B. Mukhopadhyay, J. F. Sanz, C. B. Musgrave, “First-principles calculations of structural and electronic properties of monoclinic hafnia surfaces” *Phys. Rev. B, Condens. Matter* **2006**, *73*, 115330.

[32] B. C. J Howard, R. J. Hill, B. E. Reichert, “Structures of the ZrO_2 Polymorphs at Room Temperature by High-Resolution Neutron Powder Diffraction” *Acta Cryst. B* **1984**, *44*, 116–120.
